# A Dynamic 6,000-Year Genetic History of Eurasia’s Eastern Steppe

**DOI:** 10.1016/j.cell.2020.10.015

**Published:** 2020-11-12

**Authors:** Choongwon Jeong, Ke Wang, Shevan Wilkin, William Timothy Treal Taylor, Bryan K. Miller, Jan H. Bemmann, Raphaela Stahl, Chelsea Chiovelli, Florian Knolle, Sodnom Ulziibayar, Dorjpurev Khatanbaatar, Diimaajav Erdenebaatar, Ulambayar Erdenebat, Ayudai Ochir, Ganbold Ankhsanaa, Chuluunkhuu Vanchigdash, Battuga Ochir, Chuluunbat Munkhbayar, Dashzeveg Tumen, Alexey Kovalev, Nikolay Kradin, Bilikto A. Bazarov, Denis A. Miyagashev, Prokopiy B. Konovalov, Elena Zhambaltarova, Alicia Ventresca Miller, Wolfgang Haak, Stephan Schiffels, Johannes Krause, Nicole Boivin, Myagmar Erdene, Jessica Hendy, Christina Warinner

**Affiliations:** 1 Department of Archaeogenetics, Max Planck Institute for the Science of Human History, Jena 07745, Germany; 2 School of Biological Sciences, Seoul National University, Seoul 08826, Republic of Korea; 3 Department of Archaeology, Max Planck Institute for the Science of Human History, Jena 07745, Germany; 4 Department of Anthropology, University of Colorado Boulder, Boulder, CO 80309, USA; 5 Museum of Anthropological Archaeology, University of Michigan, Ann Arbor, MI 48109, USA; 6 Department of Archaeology and Anthropology, Rheinische Friedrich-Wilhelms-Universität Bonn, Bonn 53113, Germany; 7 Institute of Archaeology, Mongolian Academy of Sciences, Ulaanbaatar 14200, Mongolia; 8 Mongolian University of Science and Technology, Ulaanbaatar 14191, Mongolia; 9 Department of Archaeology, Ulaanbaatar State University, Bayanzurkh district, Ulaanbaatar 13343, Mongolia; 10 Department of Anthropology and Archaeology, National University of Mongolia, Ulaanbaatar 14201, Mongolia; 11 International Institute for the Study of Nomadic Civilizations, Ulaanbaatar 14200, Mongolia; 12 National Centre for Cultural Heritage of Mongolia, Ulaanbaatar 14200, Mongolia; 13 Institute of History and Ethnology, Mongolian Academy of Sciences, Ulaanbaatar 14200, Mongolia; 14 University of Khovd, Khovd province, Khovd 84179, Mongolia; 15 Institute of Archaeology, Russian Academy of Sciences, Moscow 119991, Russia; 16 Institute of History, Archaeology and Ethnology, Far East Branch of the Russian Academy of Sciences, Vladivostok 690001, Russia; 17 Institute for Mongolian, Buddhist and Tibetan Studies, Siberian Branch of the Russian Academy of Sciences, Ulan-Ude 670047, Russia; 18 Department of Museology and Heritage, Faculty of Social and Cultural Activities, Heritage, and Tourism, Federal State Budgetary Educational Institution of Higher Education, East Siberian State Institute of Culture, Ulan-Ude 670031, Russia; 19 Department of Anthropology, University of Michigan, Ann Arbor, MI 48109, USA; 20 Faculty of Biological Sciences, Friedrich Schiller University, Jena 02134, Germany; 21 BioArCh, Department of Archaeology, University of York, York YO10 5NG, UK; 22 Department of Anthropology, Harvard University, Cambridge, MA 02138, USA

**Keywords:** human population history, ancient DNA, migration, nomadic pastoralists, Eastern Steppe, Mongolia, Xiongnu empire, Mongol empire

## Abstract

The Eastern Eurasian Steppe was home to historic empires of nomadic pastoralists, including the Xiongnu and the Mongols. However, little is known about the region’s population history. Here, we reveal its dynamic genetic history by analyzing new genome-wide data for 214 ancient individuals spanning 6,000 years. We identify a pastoralist expansion into Mongolia ca. 3000 BCE, and by the Late Bronze Age, Mongolian populations were biogeographically structured into three distinct groups, all practicing dairy pastoralism regardless of ancestry. The Xiongnu emerged from the mixing of these populations and those from surrounding regions. By comparison, the Mongols exhibit much higher eastern Eurasian ancestry, resembling present-day Mongolic-speaking populations. Our results illuminate the complex interplay between genetic, sociopolitical, and cultural changes on the Eastern Steppe.

## Highlights

• Genome-wide analysis of 214 ancient individuals from Mongolia and the Baikal region
• Three genetically distinct dairy pastoralist groups in Late Bronze Age Mongolia
• Xiongnu nomadic empire formed through mixing of distinct local and distant groups
• No selection on the lactase persistence alleles despite 5,000 years of dairy culture

## Graphical Abstract

**Figure:**
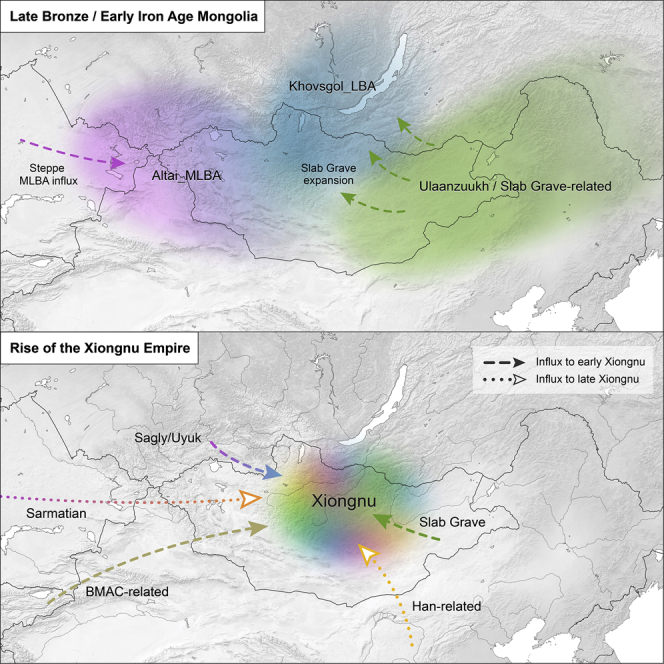

## Teaser

Ancient DNA from over 200 individuals inhabiting the Eastern Eurasian Steppe during the Bronze Age provides insights into the population history of the Mongols and Xiongnu.

## Introduction

**Figure 1:**
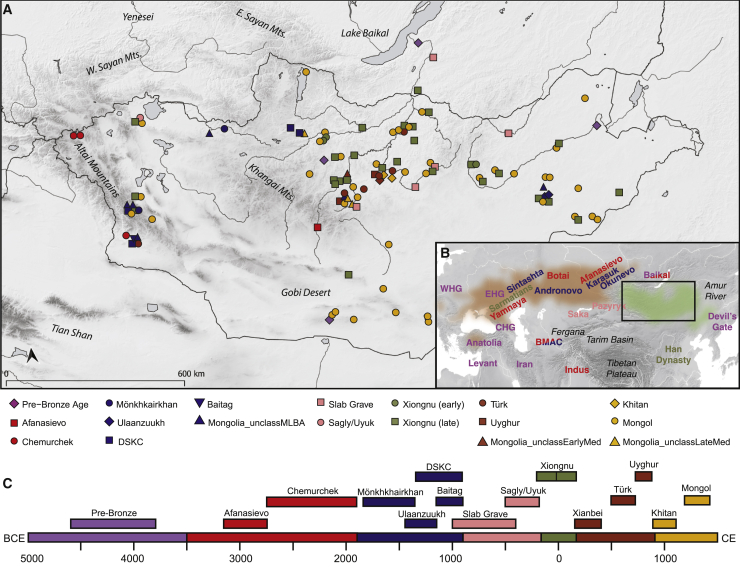
Overview of Ancient Populations and Time Periods (A) Distribution of sites with their associated culture and time period indicated by color: Pre-Bronze, purple; Early Bronze, red; Middle/Late Bronze, blue; Early Iron, pink; Xiongnu, green; Early Medieval, brown; Late Medieval, gold (see STAR Methods). See Figure S1A and Table S1B for site codes and labels. (B) Inset map of Eurasia indicating area of present study (box) and the locations of other ancient populations referenced in the text, colored by time period. The geographic extent of the Western/Central Steppe is indicated in light brown, and the Eastern Steppe is indicated in light green. (C) Timeline of major temporal periods and archaeological cultures in Mongolia. Site locations have been jittered to improve visibility of overlapping sites.

**Figure S1:**
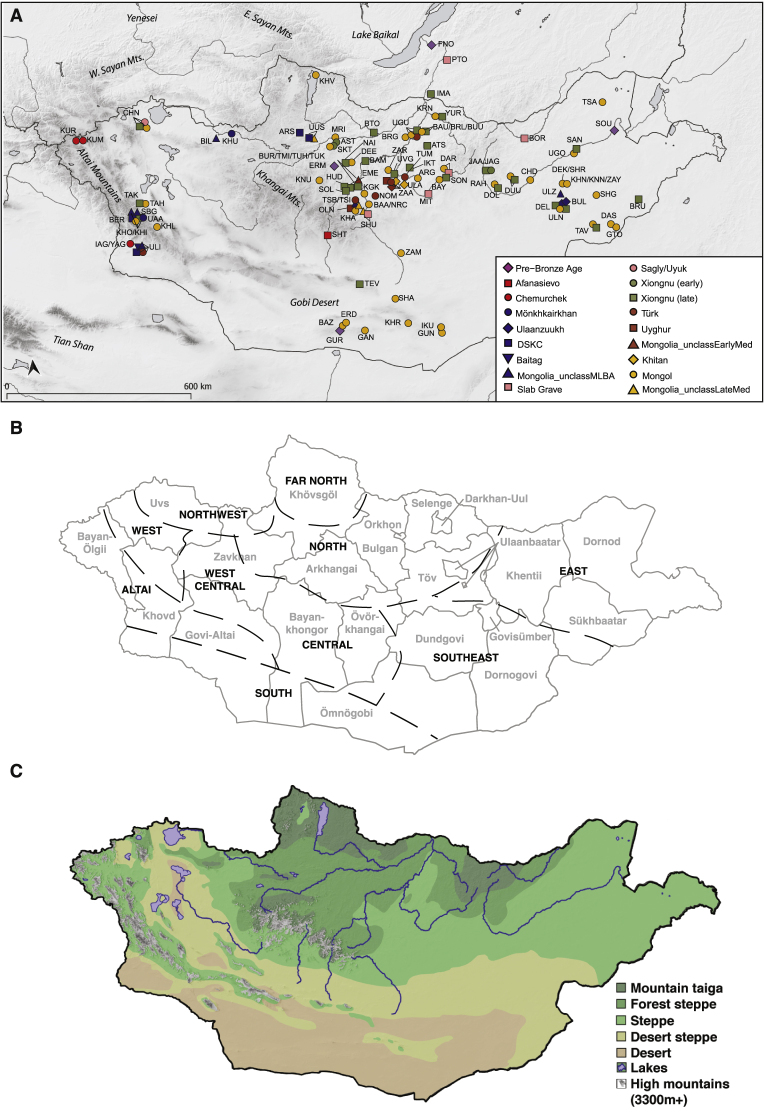
Archaeological Sites and Geographic and Ecological Features in Mongolia, Related to Figure 1 (A) Archaeological sites in Mongolia and neighboring regions analyzed in this study. (B) Mongolian regions and provinces (aimags). Provinces are indicated by gray lines and text. Regions are indicated by black dashed lines and text following the definitions of (Taylor et al., 2019). (C) Ecological zones of Mongolia. Map produced using QGIS software (v3.6) with ecological data from (Dorjgotiv, 2004).

Recent paleogenomic studies have revealed a dynamic population history on the Eurasian Steppe, with continental-scale migration events on the Western Steppe coinciding with Bronze Age transformations of Europe, the Near East, and the Caucasus (Allentoft et al., 2015; Damgaard et al., 2018a; 2018b; Haak et al., 2015; Mathieson et al., 2015; Wang et al., 2019). However, despite advances in understanding the genetic prehistory of the Western Steppe, the prehistoric population dynamics on the Eastern Steppe remain poorly understood (Damgaard et al., 2018a; Jeong et al., 2018; Rogers, 2016). The Eastern Steppe is a great expanse of grasslands, forest steppe, and desert steppe extending more than 2,500 km (Figure 1; Figure S1). While also covering parts of modern-day China and Russia, most of the Eastern Steppe falls within the national boundaries of present-day Mongolia. Recent paleogenomic studies suggest that the eastern Eurasian forest steppe zone was genetically structured during the Pre-Bronze and Early Bronze Age periods, with a strong west-east admixture cline of ancestry stretching from Botai in central Kazakhstan to Lake Baikal in southern Siberia to Devil’s Gate Cave in the Russian Far East (Damgaard et al., 2018a; Jeong et al., 2018; Sikora et al., 2019; Siska et al., 2017).

During the Bronze Age, the multi-phased introduction of pastoralism drastically changed lifeways and subsistence on the Eastern Steppe (Honeychurch, 2015; Kindstedt and Ser-Od, 2019). A recent large-scale paleoproteomic study has confirmed milk consumption in Mongolia prior to 2500 BCE by individuals affiliated with the Afanasievo (ca. 3000 BCE) and Chemurchek (2750–1900 BCE) cultures (Wilkin et al., 2020a). Although Afanasievo groups in the Upper Yenisei region have been genetically linked to the Yamnaya culture of the Pontic-Caspian steppe (ca. 3300–2200 BCE) (Allentoft et al., 2015; Morgunova and Khokhlova, 2013; Narasimhan et al., 2019), the origins of the Chemurchek have been controversial (Kovalev, 2014). Once introduced, ruminant dairying became widespread by the Middle/Late Bronze Age (MLBA, here defined as 1900–900 BCE), being practiced in the west and north at sites associated with Deer Stone-Khirigsuur Complex (DSKC) and in the east in association with the Ulaanzuukh culture (Jeong et al., 2018; Wilkin et al., 2020a). The relationships between DSKC and Ulaanzuukh groups are poorly understood, and little is known about other MLBA burial traditions in Mongolia, such as the Mönkhkhairkhan and Baitag. By the mid-first millennium BCE, the previous MLBA cultures were in decline, and Early Iron Age cultures emerged: the Slab Grave culture (ca. 1000–300 BCE) of eastern/southern Mongolia, whose burials sometimes incorporate uprooted materials from DSKC monuments (Fitzhugh, 2009; Honeychurch, 2015; Tsybiktarov, 2003; Volkov, 2002), and the Sagly/Uyuk culture (ca. 500–200 BCE) of the Sayan mountains to the northwest (also known as the Sagly-Bazhy culture, or Chandman culture in Mongolia), who had strong cultural ties to the Pazyryk (ca. 500–200 BCE) and Saka (ca. 900–200 BCE) cultures of the Altai and eastern Kazakhstan (Savinov, 2002; Tseveendorj, 2007).

From the late first millennium BCE onward, a series of hierarchical and centrally organized empires arose on the Eastern Steppe, notably the Xiongnu (209 BCE–98 CE), Türkic (552–742 CE), Uyghur (744–840 CE), and Khitan (916–1125 CE) empires. The Xiongnu empire was the first such polity in the steppe, whose drastic expansions into northern China, southern Siberia, and deep into Central Asia had a profound impact on the demographics and geopolitics of Eurasia. The Mongol empire, emerging in the thirteenth century CE, was the last and most expansive of these regimes, eventually controlling vast territories and trade routes stretching from China to the Mediterranean. However, due to a lack of large-scale genetic studies, the origins and relationships of the people who formed these states, including both the ruling elites and local commoners, remain obscure.

To clarify the population dynamics on the Eastern Steppe since prehistory, we generated and analyzed genome-wide genetic datasets for 214 individuals from 85 Mongolian and 3 Russian sites spanning approximately 6,000 years of time (ca. 4600 BCE to 1400 CE) (Tables S1, S2, and S3A). To this, we added recently published genomic data for 19 Bronze Age individuals from northern Mongolia (Jeong et al., 2018), as well as datasets from neighboring ancient populations in Russia and Kazakhstan (Damgaard et al., 2018a; 2018b; Narasimhan et al., 2019; Sikora et al., 2019; Unterländer et al., 2017) (Tables S3B and S3C), which we analyze together with worldwide modern reference populations (Table S3C). We also generated 30 new accelerator mass spectrometry dates, supplementing 74 previously published radiocarbon dates (Jeong et al., 2018; Taylor et al., 2019), for a total of 98 directly dated individuals (104 total dates) in this study (Table S4).

## Results

### Pre-Bronze Age Population Structure and the Arrival of Pastoralism

**Figure 2:**
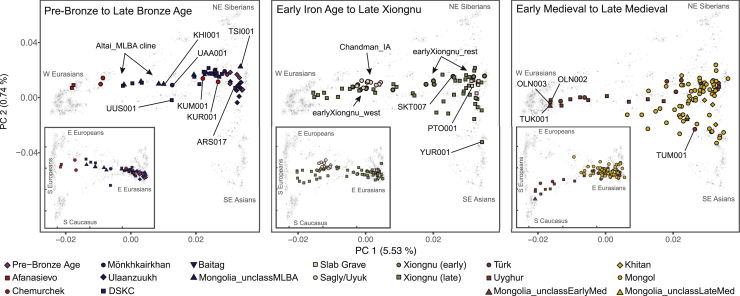
Genetic Structure of Mongolia through Time PCA of ancient individuals (n = 214) from three major periods projected onto contemporary Eurasians (gray symbols). Main panels display PC1 versus PC2; insets display PC1 versus PC3. Inset tick marks for PC1 correspond to those for the main panels; PC3 accounts for 0.35% of variation. See Figure S3B for population, sample, and axis labels, and Tables S1B, S1C, and S2A for further site and sample details.

**Figure 3:**
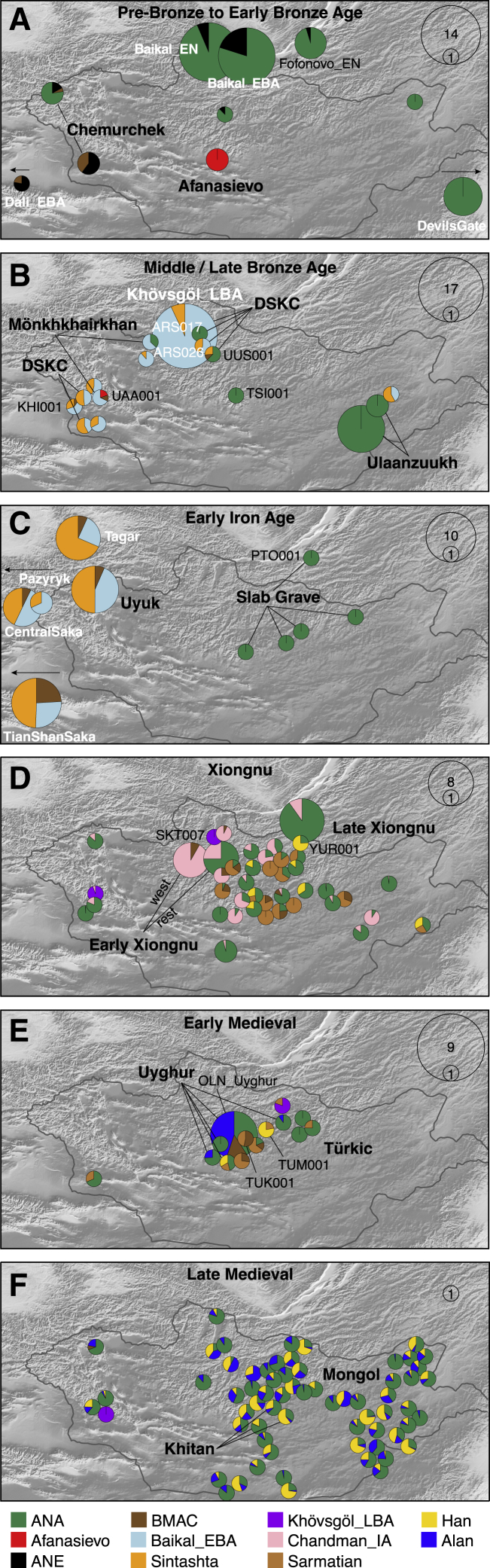
Genetic Changes in the Eastern Steppe across Time Characterized by qpAdm (A–F) Major time periods: (A) Pre-Bronze through Early Bronze Age, (B) Middle/Late Bronze Age, (C) Early Iron Age, (D) Xiongnu period, (E) Early Medieval, and (F) Late Medieval. Modeled ancestry proportions are indicated by sample size-scaled pie charts, with ancestry source populations shown below (see STAR Methods). The sample size range for each panel is indicated in the upper right. For (B) and (C), Baikal_EBA is modeled as light blue; in (D–F), Khövsgöl_LBA (purple) and the Sagly/Uyuk of Chandman_IA (pink) are modeled as new sources (Figure 4). Cultural groups are indicated by bold text. For (D–F), individuals are Late Xiongnu, Türkic, and Mongol, respectively, unless otherwise noted. Previously published reference populations are noted with white text; all others are from this study. Populations beyond the map borders are indicated by arrows. Burial locations have been jittered to improve visibility of overlapping individuals.

**Figure 4:**
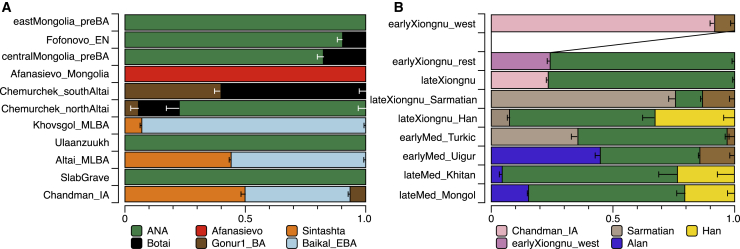
Genetic Ancestry Changes in Chronological Order across All Newly Reported Genetic Groups Well-fitted modeling results for grouped-based population genetics analyses for (A) prehistoric periods and (B) historic periods. The number of individuals in each genetic group is given in Table S3A. Raw ancestry proportions and standard error estimates are provided in Table S5. Horizontal bars represent ± 1 standard error (SE) estimated by qpAdm.

In this study, we analyzed six pre-Bronze Age hunter-gatherer individuals from three sites dating to the fifth and fourth millennia BCE: one from eastern Mongolia (SOU001, “eastMongolia_preBA,” 4686–4495 cal. BCE), one from central Mongolia (ERM003, “centralMongolia_preBA,” 3781–3639 cal. BCE), and four from the eastern Baikal region (“Fofonovo_EN”). By comparing these genomes to previously published ancient and modern data across Eurasia (Figure 2; Table S3C), we found that they are most closely related to contemporaneous hunter-gatherers from the western Baikal region (“Baikal_EN,” 5200–4200 BCE) and the Russian Far East (“DevilsCave_N,” ca. 5700 BCE), filling in the geographic gap in the distribution of this genetic profile (Figure 3A). We refer to this profile as “Ancient Northeast Asian” (ANA) to reflect its geographic distribution relative to another widespread mid-Holocene genetic profile known as “Ancient North Eurasian” (ANE), which is found among the Pleistocene hunter-gatherers of the Mal’ta (ca. 24500–24100 BP) and Afontova Gora (ca. 16900–16500 BP) sites in Siberia (Fu et al., 2016; Raghavan et al., 2015) and the horse-herders of Botai, Kazakhstan (ca. 3500–3300 BCE) (Damgaard et al., 2018a). In principal component analysis (PCA) (Figure 2), ancient ANA individuals fall close to the cluster of present-day Tungusic- and Nivkh-speaking populations in northeast Asia, indicating that their genetic profile is still present in indigenous populations of the Far East today (Figure S3A). EastMongolia_preBA is genetically indistinguishable from the ANA group DevilsCave_N (Figures 3A and 4A; Figure S4A; Table S5A), whereas Fofonovo_EN and the slightly later centralMongolia_preBA both derive a minority (12%–17%) of their ancestry from ANE-related (Botai-like) groups with the remainder of their ancestry (83%–87%) characterized as ANA (Figures 3A and 4A; Table S5A). Reanalyzing published data from the western Baikal early Neolithic Kitoi culture (Baikal_EN) and the early Bronze Age Glazkovo culture (Baikal_EBA) (Damgaard et al., 2018a), we find that they have similar ancestry profiles and a slight increase in ANE ancestry through time (from 6.4% to 20.1%) (Figure 3A).

**Figure S2:**
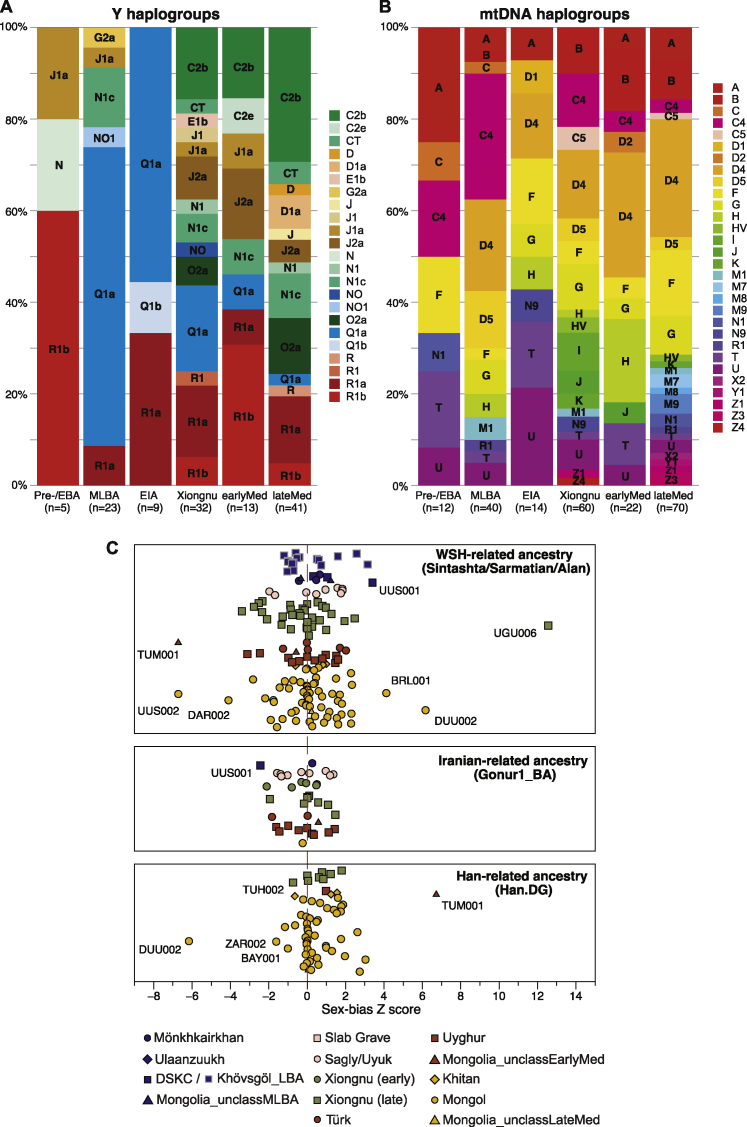
Uniparental Haplogroup Assignments by Group and Sex-Bias *Z* Scores, Related to Figure 5B and STAR Methods (A and B) Population structure from uniparentally inherited markers. (A) Distribution of Y haplogroups across each period. (B) Distribution of mitochondrial haplogroups across each period. (C) Sex-bias Z scores by evaluating the differences of WSH-/Iranian-/Han-related ancestry on the autosomes and the X chromosome. We calculated Z-score for each ancient individual who has genetic admixture with any of the three ancestries. Positive scores suggest more WSH-/Iranian-/Han-related ancestry on the autosomes, i.e., male-driven admixture.

**Figure S3:**
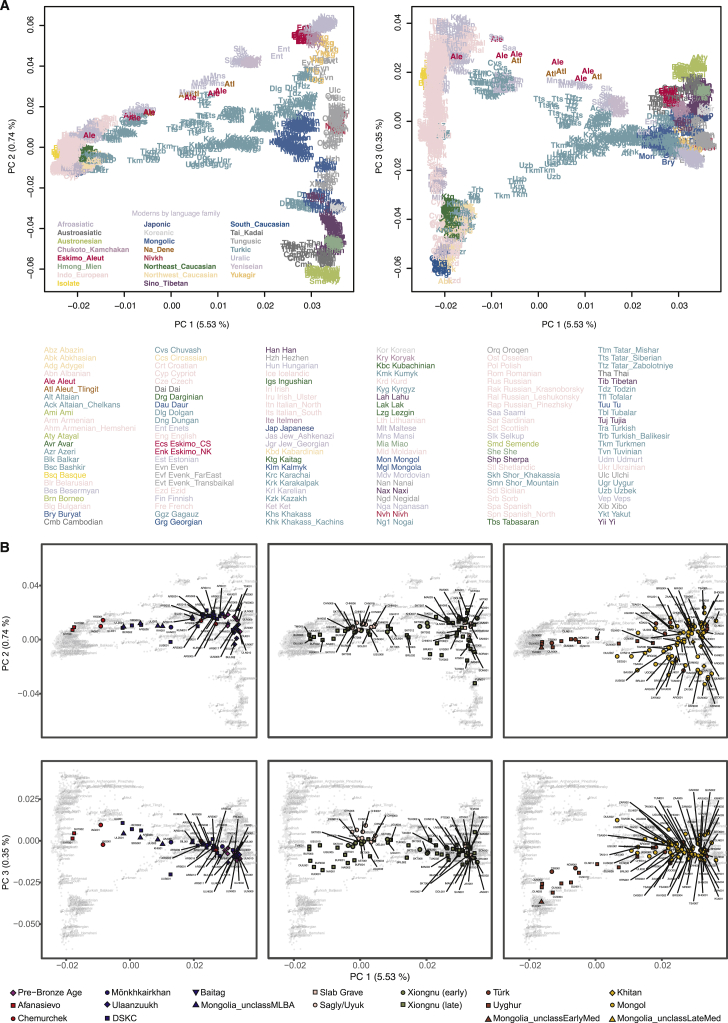
PCA of Present-Day Eurasian Populations and Genetic Structure of Mongolia through Time, Related to Figure 2 (A) PCA of present-day Eurasian populations used as the background for Figure 2 and Figure S3B. Here we show the population labels for the 2,077 Eurasian individuals used for calculating PCs and plotted as gray dots in Figure 2. Each three-letter code in the plot represents a single individual. Population IDs matching to the three-letter codes are listed at the bottom. (B) Genetic structure of Mongolia through time. Principal component analysis (PCA) of ancient individuals (n = 214) from three major periods projected onto contemporary Eurasians (gray symbols). Projection and axis variance corresponds to Figure 2. Population labels are positioned over the mean coordinate across individuals belonging to each population.

**Figure S4:**
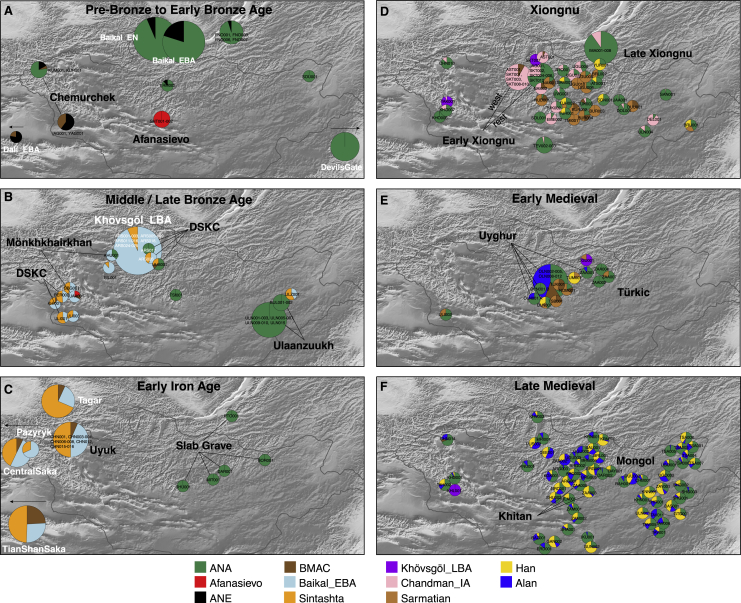
Genetic Changes in the Eastern Steppe across Time Characterized by qpAdm with All Individuals Indicated, Related to Figures 3 and 4 (A) Pre-Bronze through Early Bronze Age; (B) Middle/Late Bronze Age; (C) Early Iron Age; (D) Xiongnu period; (E) Early Medieval; (F) Late Medieval. Modeled ancestry proportions are indicated by sample size-scaled pie charts, with ancestry source populations shown below. Cultural groups are indicated by bold text. For panels (D–F), individuals are Late Xiongnu, Türkic, and Mongol, respectively, unless otherwise noted. Previously published reference populations are noted with white text; all others are from this study. Populations beyond the map borders are indicated by arrows. Burial locations have been jittered to improve visibility of overlapping individuals. Zoom in to see individual labels. Here we report results from admixture models that include all ancestry components required to explain historic late Medieval individuals as a group for unbiased cross comparison between individuals. Individual results with simpler admixture models can be found in Table S5J. See modeling details in Section 7.

**Figure S5:**
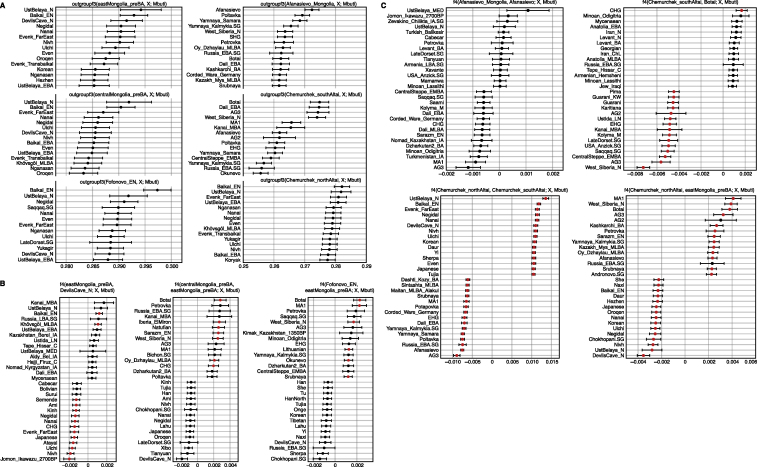
Outgroup *f3***-**Statistics and Cladality Testing using f4**-**Statistics, Related to Figures 3 and 4 (A) Outgroup *f3*-statistics for the pre-Bronze Age to Early Bronze Age groups in the Eastern Steppe. We show top 15 outgroup *f3*-statistics of the form *f3*(Target, world-wide; Mbuti) out of 345 ancient and present-day populations for the six target groups: eastMongolia_preBA, centralMongolia_preBA, Fofonovo_EN, Afanasievo_Mongolia, Chemurchek_southAltai and Chemurchek_northAltai. Horizontal bars represent ± 1 standard error (SE) calculated by 5 cM block jackknifing. (B) Testing cladality of the four ANA populations using *f4*-statistics. We show top and bottom 15 symmetric *f4*-statistics of the form *f4*(ANA1, ANA2; world-wide, Mbuti) out of 345 ancient and present-day populations for the four ANA-related target groups: eastMongolia_preBA, centralMongolia_preBA, Fofonovo_EN, DevilsCave_N. Horizontal bars represent ± 1 standard error (SE) calculated by 5 cM block jackknifing. *f4*-statistics with Z-score > 3 are highlighted in red. (C) Testing cladality of Afanasievo and Chemurchek using *f4*-statistics. We show top and bottom 15 symmetric *f4*-statistics for the three target groups Afanasievo_Mongolia, Chemurchek_southAltai and Chemurchek_northAltai, in the form *f4*(Afanasievo_Mongolia, Afanasievo; world-wide, Mbuti), *f4*(Chemurchek_southAltai, Botai; world-wide, Mbuti), *f4*(Chemurchek _northAltai, Chemurchek_southAltai; world-wide, Mbuti), and *f4*(Chemurchek _northAltai, eastMongolia_preBA; world-wide, Mbuti) out of 345 ancient and present-day populations. Horizontal bars represent ± 1 standard error (SE) calculated by 5 cM block jackknifing. *f4*-statistics with Z-score > 3 are highlighted in red.

Pastoralism in Mongolia is often assumed to have been introduced by the eastward expansion of Western Steppe cultures (e.g., Afanasievo) via either the Upper Yenisei and Sayan mountain region to the northwest of Mongolia or through the Altai mountains in the west (Janz et al., 2017). Although the majority of Afanasievo burials reported to date are located in the Altai mountains and Upper Yenisei regions, the Early Bronze Age (EBA) site of Shatar Chuluu in the southern Khangai Mountains of central Mongolia has yielded Afanasievo-style graves with proteomic evidence of ruminant milk consumption (Wilkin et al., 2020a) and a western Eurasian mitochondrial haplogroup (Rogers et al., 2020). Analyzing two of these individuals (Afanasievo_Mongolia, 3112–2917 cal. BCE), we find that their genetic profiles are indistinguishable from that of published Afanasievo individuals from the Yenisei region (Allentoft et al., 2015; Narasimhan et al., 2019) (Figure 2; Figure S5C; Table S5B), and thus these two Afanasievo individuals confirm that the EBA expansion of Western Steppe herders (WSH) extended a further 1,500 km eastward beyond the Altai into the heart of central Mongolia (Figure 3A).

**Figure S6:**
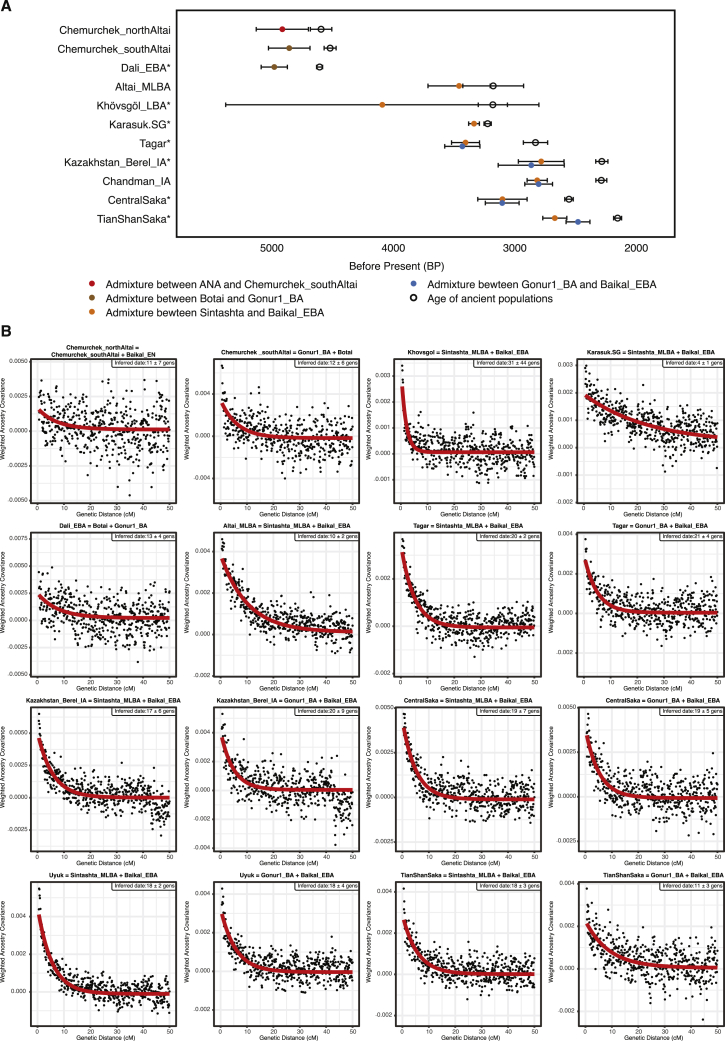
Dating Admixture in Prehistoric Individuals, Related to STAR Methods (A) Dating admixture in prehistoric individuals. We estimated admixture dates using the DATES program and converted it by adding the age of each ancient population (mean value of the center of the 95% confidence interval of calibrated ^14^C dates) and assuming 29 years per generation. Horizontal bars associated with the admixture dates (colored circles) are estimated by the square root of summing the variance of DATES estimate using leave-one-chromosome-out jackknifing method and the variance of the ^14^C date estimate, assuming that the two quantities are independent. Published groups are marked with an asterisk (^∗^). For the Chemurchek_northAltai, we used Baikal_EN as the representative of ANA ancestry for dating the admixture event, given the larger sample size of Baikal_EN. (B) Ancestry covariance in prehistoric individuals. We show the weighted ancestry covariance (y axis) calculated from DATES which is expected to decay exponentially along genetic distance (x axis) with a decay rate indicating the time since admixture, and fitted exponential curves (shown in red line). We start the fit at genetic distance at 0.45 centiMorgans, and estimate standard error by a weighted block jackknife removing one chromosome in each run.

The succeeding EBA Chemurchek culture (2750–1900 BCE), a ruminant dairying society (Wilkin et al., 2020a) whose mortuary features include stone slabs and anthropomorphic stelae, has also been purportedly linked to WSH migrations (Kovalev and Erdenebaatar, 2009). Chemurchek graves are found throughout the Altai and in the Dzungarian Basin in Xinjiang, China (Jia and Betts, 2010; Kovalev, 2014; 2015). We analyzed two Chemurchek individuals from the southern Altai site of Yagshiin Huduu and two individuals from the northern Altai sites of Khundii Gobi (KUM001) and Khuurai Gobi 2 (KUR001). Compared to Afanasievo_Mongolia, the Yagshiin Huduu individuals also show a high degree of Western ancestry but are displaced in PCA (Figure 2) and have a strong genetic affinity with ANE-related ancient individuals such as AfontovaGora3 (AG3), West_Siberia_N, and Botai (Figure 3A; Figures S5A and S5C). We find that the Yagshiin Huduu Chemurchek individuals (“Chemurchek_southAltai”) are genetically similar to Dali_EBA (Figure 3A), a contemporaneous individual from eastern Kazakhstan (Narasimhan et al., 2019). The genetic profiles of both the Yagshiin Huduu and Dali_EBA individuals are well fitted by two-way admixture models with Botai (60%–78%) and groups with ancient Iranian-related ancestry, such as Gonur1_BA from Gonur Tepe, a key EBA site of the Bactria-Margiana Archaeological Complex (BMAC) (22%–40%; Figure 3A; Table S5B). Although minor genetic contributions from the Afanasievo-related groups cannot be excluded, Iranian-related ancestry is required for all fitting models, and this admixture is estimated to have occurred 12 ± 6 generations earlier (∼336 ± 168 years; Figure S6) when modeled using DATES (Narasimhan et al., 2019). However, because all proxy source populations used in this modeling are quite distant in either time or space from the EBA Altai, the proximate populations contributing to the Chemurchek cannot yet be precisely identified. In the northern Altai, the two Chemurchek individuals (“Chemurchek_northAltai”) have mostly ANA-derived ancestry (∼80%), with the remainder resembling that of the southern Altai Chemurchek individuals (Figures 3A and 4A; Table S5B). As such, we observe genetic heterogeneity among Chemurchek individuals by geographic location.

Although based on a small number of genomes, we find that neither the Afanasievo nor the Chemurchek left enduring genetic traces into the subsequent MLBA. This is strikingly different than in Europe, where migrating EBA steppe herders had a transformative and lasting genetic impact on local populations (Allentoft et al., 2015; Haak et al., 2015; Mathieson et al., 2018). In the Eastern Steppe, the transient genetic impact of the EBA herders stands in sharp contrast to their strong and enduring cultural and economic impact given that the cultural features that EBA pastoralists first introduced, such as mortuary mound building and dairy pastoralism, continue to the present day.

### Bronze Age Emergence of a Tripartite Genetic Structure

Previously, we reported a shared genetic profile among EBA western Baikal hunter-gatherers (Baikal_EBA) and Late Bronze Age (LBA) pastoralists in northern Mongolia (Khövsgöl_LBA) (Jeong et al., 2018). This genetic profile, composed of major and minor ANA and ANE ancestry components, respectively, is also shared with the earlier eastern Baikal (Fofonovo_EN) and Mongolian (centralMongolia_preBA) groups analyzed in this study (Figures 3A, 3B, and 4A), suggesting a regional persistence of this genetic profile for nearly three millennia. Centered in northern Mongolia, this genetic profile is distinct from that of other Bronze Age groups. Overall, we find three distinct and geographically structured gene pools in LBA Mongolia, with the Khövsgöl_LBA population representing one of them (Figures 3B and 4A). The other two, which we refer to as “Altai_MLBA” and “Ulaanzuukh_SlabGrave,” are described below.

During the MLBA (1900–900 BCE), as grasslands expanded in response to climate change, new pastoralist cultures expanded out of inner-montane regions and across the Eastern Steppe (Kindstedt and Ser-Od, 2019). This period is also notable for the first regional evidence of horse milking (ca. 1200 BCE; Wilkin et al., 2020a), which is today exclusively associated with alcohol (*airag*) production (Bat-Oyun et al., 2015), and a dramatic intensification of horse use, including the emergence of mounted horseback riding, which would have substantially extended the accessibility of remote regions of the steppe. In the Altai-Sayan region, dairy pastoralists associated with DSKC and other unclassified MLBA burial types (Altai_MLBA, n = 7) show clear genetic evidence of admixture between a Khövsgöl_LBA-related ancestry and a Sintashta-related WSH ancestry (Figure 3B; Figure S4B). Overall, they form an “Altai_MLBA” cline on PCA between Western Steppe groups and the Baikal_EBA/Khövsgöl_LBA cluster (Figure 2), with their position varying on PC1 according to their level of Western ancestry (Table S5C).

This is the first appearance on the Eastern Steppe of a Sintashta-like ancestry (frequently referred to as “steppe_MLBA” in previous studies), which is distinct from prior Western ancestries present in the Afanasievo and Chemurchek populations and instead shows a close affinity to European Corded-Ware populations and later Andronovo-associated groups, such as the Sintashta (Allentoft et al., 2015). In Khovd province, individuals belonging to DSKC and unclassified MLBA groups (BER002 and SBG001, respectively) have a similar genetic profile that is best modeled as an equal mixture of Khövsgöl_LBA and Sintashta (Figure 3B; Table S5C). This genetic profile matches that previously described for a genetic outlier in northern Mongolia that deviated from the Khövsgöl_LBA cluster in a previous study (ARS026; Jeong et al., 2018). An additional four Altai_MLBA individuals belonging to DSKC (ULI001) and unclassified MLBA groups (BIL001, ULI003, ULZ001) also fit this admixture model with varying admixture proportions (Table S5C). Taken together, the Altai_MLBA cline reveals the ongoing mixture of two source populations: a Sintashta/Andronovo-related WSH population and a local population represented by Khövsgöl_LBA. The admixture is estimated to have occurred only 10 ± 2 generations (∼290 years) before the individuals analyzed in this study, a finding consistent with their heterogeneous ancestry proportions (Figure S6). Because the Sintashta culture (ca. 2200–1700 BCE) is associated with novel transportation technologies, such as horse-drawn chariots (Anthony, 2010), the appearance of this ancestry profile on the Eastern Steppe suggests that heightened mobility capabilities played an important role in linking diverse populations across the Eurasian Steppe (Honeychurch, 2015).

Three MLBA individuals in our dataset present genetic profiles that cannot be fully explained by the Altai_MLBA cline. These three, two Altai individuals (UAA001 and KHI001) and UUS001 from Khövsgöl province, are better modeled with a small contribution from Gonur1_BA as a third ancestry source (Table S5C). Taken together, although cultural differences may have existed among the major MLBA mortuary traditions of the Altai and northern Mongolia (Mönkhkhairkhan, DSKC, and unclassified MLBA), they do not form distinct genetic groups.

The populations making up the heterogeneous Altai_MLBA cline left descendants in the Altai-Sayan region, who we later identify at the Sagly/Uyuk site of Chandman Mountain (“Chandman_IA,” ca. 400–200 BCE) in northwestern Mongolia during the Early Iron Age (EIA). Nine Chandman_IA individuals form a tight cluster on PCA at the end of the previous Altai_MLBA cline away from Khövsgöl_LBA cluster (Figure 2). During the EIA, the Sagly/Uyuk were pastoralists and millet agropastoralists largely centered in the Upper Yenisei region of present-day Tuva. Together with the Pazyryk of the Altai and the Saka of eastern Kazakhstan, they formed part of a broader Scythian cultural phenomenon that stretched across the Western Steppe, Tarim Basin, and Upper Yenesei (Parzinger, 2006).

We find that EIA Scythian populations systematically deviate from the earlier Altai_MLBA cline, requiring a third ancestral component (Figures 3C and 4A; FigureS4C). The appearance of this ancestry, related to populations of Central Asia (Caucasus/Iranian Plateau/Transoxiana regions) including BMAC (Narasimhan et al., 2019), is clearly detected in the Iron Age groups such as Central Saka, TianShan Saka, Tagar (Damgaard et al., 2018b), and Chandman_IA, while absent in the earlier DSKC and Karasuk groups (Tables S5C–S5E). This third component makes up 6%–24% of the ancestry in these Iron Age groups, and the date of admixture in Chandman_IA is estimated at ∼18 ± 4 generations earlier, ca. 750 BCE, which postdates the collapse of the BMAC ca. 1600 BCE and slightly predates the formation of the Persian Achaemenid empire ca. 550 BCE (Figure S6). We suggest that this Iranian-related genetic influx was mediated by increased contact and mixture with agropastoralist populations in the region of Transoxiana (Turan) and Fergana during the LBA to EIA transition. The widespread emergence of horseback riding during the late second and early first millennium BCE (Drews, 2004), and the increasing sophistication of horse transport thereafter, likely contributed to increased population contact and the dissemination of this Iranian-related ancestry onto the steppe. Our results do not exclude additional spheres of contact, such as increased mobility along the Inner Asian Mountain Corridor, which could have also introduced this ancestry into the Altai via Xinjiang starting in the Bronze Age (Frachetti, 2012).

In contrast to the MLBA and EIA cultures of the Altai and northern Mongolia, different burial traditions are found in the eastern and southern regions of Mongolia (Honeychurch, 2015), notably the LBA Ulaanzuukh (1450–1150 BCE) and EIA Slab Grave (1000–300 BCE) cultures. In contrast to other contemporaneous Eastern Steppe populations, we find that individuals associated with these burial types show a clear northeastern Asian (ANA-related) genetic profile lacking both ANE and WSH admixture (Figures 2, 3C, and 4). Both groups were ruminant pastoralists, and the EIA Slab Grave culture also milked horses (Wilkin et al., 2020a). The genetic profiles of Ulaanzuukh and Slab Grave individuals are genetically indistinguishable (Figure 2; Table S5C), consistent with the archaeological hypothesis that the Slab Grave tradition emerged out of the Ulaanzuukh (Honeychurch, 2015; Khatanbaatar, 2019). Both groups are also indistinguishable from the earlier eastMongolia_preBA individual dating to ca. 4600 BCE, suggesting a long-term (>4,000-year) stability of this prehistoric eastern Mongolian gene pool (Table S5C). In subsequent analyses, we merged Ulaanzuukh and Slab Grave into a single genetic group (“Ulaanzuukh_SlabGrave”). The Ulaanzuukh_SlabGrave genetic cluster is the likely source of the previously described DSKC eastern outlier from Khövsgöl province (ARS017) (Jeong et al., 2018), as well as a culturally unclassified individual (TSI001) from central Mongolia who dates to the LBA-EIA transition (Figures 2, 3B, and 3C; Table S5C). In addition, the Mönkhkhairkhan individual KHU001 from northwest Mongolia has a non-negligible amount of Ulaanzuukh_SlabGrave ancestry in addition to his otherwise Baikal_EBA ancestry (Figure S4B; Table S5C). While these three individuals attest to occasional long-distance contacts between northwestern and eastern Mongolia during the LBA, we find no evidence of Ulaanzuukh_SlabGrave ancestry in the Altai, and the overall frequency of the Ulaanzuukh_SlabGrave genetic profile outside of eastern and southern Mongolia during the MLBA is very low. During the EIA, the Slab Grave culture expanded northward, sometimes disrupting and uprooting former DSKC graves in their path (Fitzhugh, 2009; Honeychurch, 2015; Tsybiktarov, 2003; Volkov, 2002), and it ultimately reached as far north as the eastern Baikal region, which is reflected in the genetic profile of the Slab Grave individual PTO001 in this study (Figure 3C). Overall, our findings reveal a strong east-west genetic division among Bronze Age Eastern Steppe populations through the end of the Early Iron Age. Further sampling from central and southern Mongolia will help refine the spatial distribution of these ancestry profiles, as well as the representativeness of our current findings.

### The Xiongnu Empire, the Rise of the First Imperial Steppe Polity

Arising from the prehistoric populations of the Eastern Steppe, large-scale polities began to develop during the late first millennium BCE. The Xiongnu was the first historically documented empire founded by pastoralists, and its establishment is considered a watershed event in the sociopolitical history of the Eastern Steppe (Brosseder and Miller, 2011; Honeychurch, 2015). The Xiongnu held political dominance in East and Central Asia from the third century BCE through the first century CE. The cultural, linguistic, and genetic makeup of the people who constituted the Xiongnu empire has been of great interest, as has their relationship to other contemporaneous and subsequent nomadic groups on the Eastern Steppe. Here, we report genome-wide data for 60 Xiongnu-era individuals from across Mongolia and dating from ca. 200 BCE to 100 CE, thus spanning the entire period of the Xiongnu empire. Although most individuals date to the late Xiongnu period (after 50 BCE), 13 individuals predate 100 BCE and include 12 individuals from the northern early Xiongnu frontier sites of Salkhityn Am (SKT) and Atsyn Gol (AST) and one individual from the early Xiongnu site of Jargalantyn Am (JAG) in eastern Mongolia.

We observe two distinct demographic processes that contributed to the formation of the early Xiongnu. First, half of the early individuals (n = 6) form a genetic cluster (earlyXiongnu_west) resembling that of Chandman_IA of the preceding Sagly/Uyuk culture from the Altai-Sayan region (Figure 2). They derive 92% of their ancestry from Chandman_IA with the remainder attributed to additional Iranian-related ancestry, which we model using BMAC as a proxy (Figures 3D and 4D; Table S5F). This suggests that the low-level Iranian-related gene flow identified among the Chandman_IA Sagly/Uyuk during the EIA likely continued during the second half of the first millennium BCE, spreading across western and northern Mongolia. Second, six individuals (“earlyXiongnu_rest”) fall intermediate between the earlyXiongnu_west and Ulaanzuukh_SlabGrave clusters; four carry varying degrees of earlyXiongnu_west (39%–75%) and Ulaanzuukh_SlabGrave (25%–61%) related ancestry, and two (SKT004, JAG001) are indistinguishable from the Ulaanzuukh_SlabGrave cluster (Figure 3D; Tables S5F and S5G). This genetic cline linking the earlyXiongnu_west and Ulaanzuukh_SlabGrave gene pools signifies the unification of two deeply diverged and distinct lineages on the Eastern Steppe—between the descendants of the DSKC, Mönkhkhairkhan, and Sagly/Uyuk cultures in the west and the descendants of the Ulaanzuukh and Slab Grave cultures in the east. Overall, the low-level influx of Iranian-related gene flow continuing from the previous Sagly/Uyuk culture and the sudden appearance of a novel east-west mixture uniting the gene pools of the Eastern Steppe are the two defining demographic processes associated with the rise of the Xiongnu.

Among late Xiongnu individuals, we find even higher genetic heterogeneity (Figure 2), and their distribution on PC indicates that the two demographic processes evident among the early Xiongnu continued into the late Xiongnu period, but with the addition of new waves and complex directions of gene flow. Of the 47 late Xiongnu individuals, half (n = 26) can be adequately modeled by the same admixture processes seen among the early Xiongnu: 22 as a mixture of Chandman_IA+Ulaanzuukh_SlabGrave, 2 (NAI002, TUK002) as a mixture of either Chandman_IA+BMAC or Chandman_IA+Ulaanzuukh_SlabGrave+BMAC, and 2 (TUK003, TAK001) as a mixture of either earlyXiongnu_west+Ulaanzuukh_SlabGrave or earlyXiongnu_west+Khovsgol_LBA (Figures 3D and 4D; Table S5G). A further two individuals (TEV002, BUR001) also likely derive their ancestry from the early Xiongnu gene pool, although the p value of their models is slightly lower than the 0.05 threshold (Table S5G). However, a further 11 late Xiongnu with the highest proportions of western Eurasian affinity along PC1 cannot be modeled using BMAC or any other ancient Iranian-related population. Instead, they fall on a cluster of ancient Sarmatians from various locations in the Western and Central Steppe (Figure 2).

Admixture modeling confirms the presence of a Sarmatian-related gene pool among the late Xiongnu: three individuals (UGU010, TMI001, BUR003) are indistinguishable from Sarmatian, two individuals (DUU001, BUR002) are admixed between Sarmatian and BMAC, three individuals (UGU005, UGU006, BRL002) are admixed between Sarmatian and Ulaanzuukh_SlabGrave, and three individuals (NAI001, BUR004, HUD001) require Sarmatian, BMAC, and Ulaanzuukh_SlabGrave (Figure 3D; Figure S4D; Table S5G). In addition, eight individuals with the highest eastern Eurasian affinity along PC1 are distinct from both the Ulaanzuukh_SlabGrave and Khövsgöl_LBA genetic profiles, showing affinity along PC2 toward present-day people from East Asia further to the south (Figure 2). Six of these individuals (EME002, ATS001, BAM001, SON001, TUH001, YUR001) are adequately modeled as a mixture of Ulaanzuukh_SlabGrave and Han (Tables S5F and S5G), and YUR001 in particular exhibits a close genetic similarity to two previously published Han empire soldiers (Damgaard et al., 2018b), whose genetic profile we refer to as “Han_2000BP” (Table S5G). The remaining two individuals (BRU001, TUH002) are similar but also require the addition of Sarmatian ancestry (Table S5G). The late Xiongnu are thus characterized by two additional demographic processes that distinguish them from the early Xiongnu: gene flow from a new Sarmatian-related Western ancestry source and intensified interaction and mixture with people of the contemporaneous Han empire of China. A previous study of the Egyin Gol Xiongnu necropolis reported mitochondrial haplogroups of both western and eastern Eurasian origins (Keyser-Tracqui et al., 2003), and this accords with our findings of the west-east admixture from genome-wide data. Together, these results match well with historical records documenting the political influence that the Xiongnu exercised over their neighbors, including the Silk Road kingdoms of Central Asia and Han Dynasty China, as well as purported migrations both in and out of Mongolia (Miller, 2014). Overall, the Xiongnu period can be characterized as one of expansive and extensive gene flow that began by uniting the gene pools of western and eastern Mongolia and ended by uniting the gene pools of western and eastern Asia.

### Fluctuating Genetic Heterogeneity in the Post-Xiongnu Polities

After the collapse of the Xiongnu empire ca. 100 CE, a succession of nomadic pastoralist regimes rose and fell over the next several centuries across the politically fragmented Eastern Steppe: Xianbei (ca. 100–250 CE), Rouran (ca. 300–550 CE), Türkic (552–742 CE), and Uyghur (744–840 CE). Although our sample representation for the Early Medieval period is uneven, consisting of 1 unclassified individual dating to the Xianbei or Rouran period (TUK001), 8 individuals from Türkic mortuary contexts, and 13 individuals from Uyghur cemeteries, it is clear that these individuals have genetic profiles that differ from the preceding Xiongnu period, suggesting new sources of gene flow into Mongolia at this time that displace them along PC3 (Figure 2). Individual TUK001 (250–383 cal. CE), whose burial was an intrusion into an earlier Xiongnu cemetery, has the highest western Eurasian affinity. This ancestry is distinct from that of the Sarmatians and closer to ancient populations with BMAC/Iranian-related ancestry (Figure 2). Among the individuals with the highest eastern Eurasian affinity, two Türkic-period individuals and one Uyghur-period individual (ZAA004, ZAA002, OLN001.B) are indistinguishable from the Ulaanzuukh_SlabGrave cluster. Another individual (TUM001), who was recovered from the tomb ramp of an elite Türkic-era emissary of the Tang Dynasty, has a high proportion of Han-related ancestry (78%; Figures 3E and 4B; Figure S4E; Table S5H). This male, buried with two dogs, was likely a Chinese attendant sacrificed to guard the tomb entrance (Ochir et al., 2013). The remaining 17 Türkic and Uyghur individuals show intermediate genetic profiles (Figure 3E).

The high genetic heterogeneity of the Early Medieval period is vividly exemplified by 12 individuals from the Uyghur period cemetery of Olon Dov (OLN; Figure 2) in the vicinity of the Uyghur capital of Ordu-Baliq. Six of these individuals came from a single tomb (grave 19), of whom only two are related (OLN002 and OLN003, second-degree; Table S2D); the absence of closer kinship ties raises questions about the function of such tombs and the social relationships of those buried within them. Most Uyghur-period individuals exhibit a high but variable degree of west Eurasian ancestry—best modeled as a mixture of Alans, a historic nomadic pastoral group likely descended from the Sarmatians and contemporaries of the Huns (Bachrach, 1973), and an Iranian-related (BMAC-related) ancestry—together with Ulaanzuukh_SlabGrave (ANA-related) ancestry (Figure 3E). The admixture dates estimated for the ancient Türkic and Uyghur individuals in this study correspond to ca. 500 CE: 8 ± 2 generations before the Türkic individuals and 12 ± 2 generations before the Uyghur individuals (represented by ZAA001 and Olon Dov individuals).

### Rise of the Mongol Empire

After the fall of the Uyghur empire in the mid-ninth century, the Khitans of northeast China established the powerful Liao Dynasty in 916 CE. The Khitans controlled large areas of the Eastern Steppe and are recorded to have relocated people within their conquered territories (Kradin and Ivliev, 2008), but few Khitan period cemeteries are known within Mongolia. Our study includes three Khitan individuals (ZAA003, ZAA005, ULA001) from Bulgan province, all of whom have a strongly eastern Eurasian genetic profile (Figure 2), with <10% west Eurasian ancestry (Figures 3F and 4B; Table S5I). This may reflect the northeastern Asian origin of the Mongolic-speaking Khitan, but a larger sample size is required to adequately characterize the genetic profile of Khitan populations within Mongolia. In 1125 CE, the Khitan empire fell to the Jurchen’s Jin Dynasty, which was then conquered in turn by the Mongols in 1234 CE.

At its greatest extent, the Mongol empire (1206–1368 CE) spanned nearly two-thirds of the Eurasian continent. It was the world’s largest contiguous land empire, and the cosmopolitan entity comprised diverse populations that flowed into the steppe heartland. We analyzed 62 Mongol-era individuals whose burials are consistent with those of low-level, local elites. No royal or regional elite burials were included, and neither were individuals from the cosmopolitan capital of Karakorum. Although we find that Mongol-era individuals were diverse, they exhibit a much lower genetic heterogeneity than the Xiongnu-era individuals (Figure 2), and they almost entirely lack the residual ANE-related ancestry (in the form of Chandman_IA and Khövsgöl_LBA) that had been present among the Xiongnu and earlier northern/western MLBA cultures. On average, Mongol-period individuals have a much higher eastern Eurasian affinity than previous empires, and this period marks the beginning of the formation of the modern Mongolian gene pool. We find that most historic Mongols are well-fitted by a three-way admixture model with the following ancestry proxies: Ulaanzuukh_SlabGrave, Han, and Alans. Consistent with their PCA location (Figure 2), Mongol-era individuals as a group can be modeled with only 15%–18% Western Steppe ancestry (Alan or Sarmatian) but require 55%–64% Ulaanzuukh_SlabGrave and 21%–27% of Han-related ancestry (Table S5I). Applying the same model to each individual separately, this three-source model adequately explains 56 out of 61 ancient Mongols (based on p value at threshold of 0.05), as well as one unclassified Late Medieval individual dating to around the beginning of the Mongol empire (SHU002) (Table S5J).

**Figure S7:**
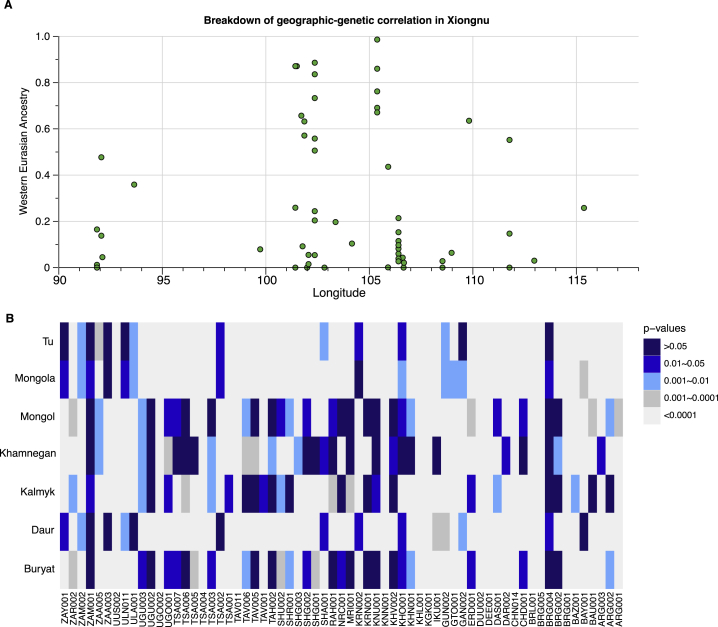
Breakdown of Geography and Genetics among Xiongnu and Comparison of Mongol Period and Present-Day Populations, Related to Figure 3 and STAR Methods (A) Breakdown of the geographic-genetic correlation in Xiongnu. We show the proportions of West Eurasian ancestry on all individuals/groups from Xiongnu era (y axis) versus the longitude of archaeological site they come from (x axis). The raw numbers of individual estimates can be found in Table S5G for models using Sarmatian as the western Eurasian source. Unlike MLBA/EIA individuals (Figure 3), Xiongnu individuals from more western sites do not have higher proportion of western Eurasian ancestry than those from eastern sites. (B) Comparing genetic homogeneity between ancient Mongol individuals and seven present-day Mongolic-speaking populations using qpWave. We report the *p-value* for every individual-based qpWave {ancient Mongol individual; Mongolic group} using seven modern Mongolic-speaking populations: Buryat, Daur, Kalmyk, Khamnegan, Mongol, Mongola, and Tu in the Human Origins dataset. When the *p-value* from qpWave is > 0.05, it suggests that the ancient individual on the y axis is genetically indistinguishable from the modern Mongolic-speaking population shown on the x axis. Smaller *p-value*s indicate that the ancient individual is significantly different from the modern group.

Since the fall of the Mongol empire in 1368 CE, the genetic profile of the Mongolian populations has not substantially changed. The genetic structure established during the Mongol empire continues to characterize present-day Mongolic-speaking populations living in both Mongolia and Russia. We examined the genetic cladality between the historic Mongols and seven present-day Mongolic-speaking groups (Mongols, Kalmyk, Buryat, Khamnegan, Daur, Tu, and Mongola) using an individual-based qpWave analysis. Within the resolution of current data, 34 of 61 historic Mongols are genetically cladal with at least one modern Mongolic-speaking population (Figure S7B). The Mongol empire had a profound impact on restructuring the political and genetic landscape of the Eastern Steppe, and these effects endured long after the decline of the empire and are still evident in Mongolia today.

### Functional and Gendered Aspects of Recurrent Admixture in the Eastern Steppe

**Figure 5:**
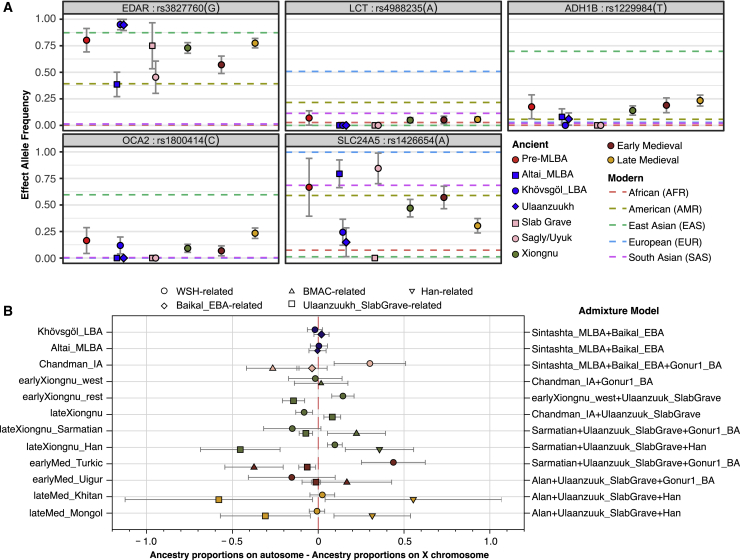
Functional Allele Frequencies and Sex-Biased Patterns of Genetic Admixture (A) Allele frequencies of five phenotypic SNP changes through time. For the effective allele, we show maximum likelihood frequency estimates and one standard error bar for each ancient group. The pre-MLBA category corresponds to the sum of all ancient groups before Mönkhkhairkhan. Xiongnu, Early Medieval, and Late Medieval correspond to the sum of all ancient groups in each period correspondingly. Horizontal dashed lines show allele frequency information from the 1000 Genomes Project’s five super populations. (B) Sex-biased patterns of genetic admixture by period and population. We calculated *Z* scores for every ancient individual who has genetic admixture with WSH-/Iranian-/Han-related ancestry. Positive scores suggest more WSH-/Iranian-/Han-related ancestry on the autosomes, i.e., male-driven admixture. See Figure S2C for individual *Z* scores.

To investigate the functional aspects of recurrent admixture on the Eastern Steppe, we estimated the population allele frequency of five SNPs associated with functional or evolutionary aspects of lactose digestion (*LCT/MCM6*), dental morphology (*EDAR*), pigmentation (*OCA2*, *SLC24A5*), and alcohol metabolism (*ADH1B*) (Figure 5A). First, we find that despite a pastoralist lifestyle with widespread direct evidence for milk consumption (Jeong et al., 2018; Wilkin et al., 2020a), the MLBA and EIA individuals of the Eastern Steppe did not have any derived mutations conferring lactase persistence. Individuals from subsequent periods did have the derived mutation that is today widespread in Europe (rs4988235) but at negligibly low frequency (∼5%) and with no increase in frequency over time (Figure 5A). This is somewhat remarkable given that, in addition to other dairy products, some contemporary Mongolian herders consume up to 4–10 L of *airag* (fermented mare’s milk, ∼2.5% lactose) per day during the summer months (Bat-Oyun et al., 2015), resulting in a daily intake of 100–250 g of lactose sugar. Petroglyph depictions of *airag* production date back to the EIA in the Yenisei Basin (Dėvlet, 1976), and accounts of the historic Mongols record abundant and frequent consumption of *airag*, as well as a wide range of additional liquid and solid ruminant dairy products (Bayarsaikhan, 2016; Onon, 2005), which has been additionally confirmed by ancient proteomic evidence (Jeong et al., 2018; Wilkin et al., 2020a). How Mongolians have been able to digest such large quantities of lactose for millennia in the absence of lactase persistence is unknown, but it may be related to their reportedly unusual gut microbiome structure, which today is highly enriched in lactose-digesting *Bifidobacterium* spp. (Liu et al., 2016).

Genetic markers that underwent regional selective sweeps show allele frequency changes that correlate with changes in the genome-wide ancestry profile (Figure 5A). For example, rs3827760 in *EDAR* (ectodysplasin A receptor) and rs1426654 in *SLC24A5* (solute carrier family 24 member 5) are well-known targets of positive selection in East Asians and western Eurasians, respectively (Sabeti et al., 2007). Our MLBA and EIA populations show a strong population differentiation in the allele frequencies of these two SNPs: rs3827760 frequency is much higher in groups with higher eastern Eurasian affinity (Khovsgol_LBA, Ulaanzuukh_SlabGrave), whereas rs1426654 is higher in Altai_MLBA and Chandman_IA (Table S2E). We find that two SNPs that have undergone more recent positive selection (Donnelly et al., 2012; Li et al., 2011) in East Asians, rs1229984 in *ADH1B* (aldehyde dehydrogenase 1B) and rs1800414 in *OCA2* (oculocutaneous albinism II), were absent or in extremely low frequency during the MLBA and EIA, when the eastern Eurasian ancestry was primarily ANA-related, but increased in frequency over time as the proportion of East Asian ancestry increased through interactions with imperial China and other groups (Table S2E).

Finally, we investigated gendered dimensions of the population history of the Eastern Steppe. Sex-biased patterns of genetic admixture can be informative about gendered aspects of migration, social kinship, and family structure. We observe a clear signal of male-biased WSH admixture among the EIA Sagly/Uyuk and during the Türkic period (i.e., more positive *Z* scores; Figure 5B), which also corresponds to the decline in the Y chromosome lineage Q1a and the concomitant rise of the western Eurasian lineages such as R and J (Figure S2A). During the later Khitan and Mongol empires, we observe a prominent male bias for East Asian-related ancestry (Figure S2C), which can also be seen from the rise in frequency of Y chromosome lineage O2a (Figure S2A). The Xiongnu period exhibits the most complex pattern of male-biased admixture, whereby different genetic subsets of the population exhibit evidence of different sources of male-biased admixture (Figure S2C).

Among the Xiongnu, we also detect 10 genetic relative pairs, including a father-daughter pair buried in the same grave (JAG001 and JAA001) at Jargalantyn Am, as well as a mother-son pair (IMA002 and IMA005) at Il’movaya Pad, a brother-sister pair (TMI001 and BUR003) at Tamiryn Ulaan Khoshuu, and a brother-brother pair (SKT002 and SKT006) at Salkhityn Am (Table S2D). Of the remaining six pairs, three are female-female relative pairs buried within the same site, suggesting the presence of extended female kinship within Xiongnu groups. First-degree relatives within a single site have also been reported in a previous study on the Egyin Gol Xiongnu necropolis based on the autosomal short tandem repeat (STR) data (Keyser-Tracqui et al., 2003). These relationships, when combined with mortuary features, offer the first clues to local lineage and kinship structures within the Xiongnu empire, which are otherwise poorly understood.

## Discussion

The population history of the Eastern Steppe is one marked by the repeated mixing of diverse eastern and western Eurasian gene pools. However, rather than simple waves of migration, demographic events on the Eastern Steppe have been complex and variable. Generating more than 200 genome-wide ancient datasets, we have presented the first genetic evidence of this dynamic population history, from ca. 4600 BCE through the end of the Mongol empire. We found that the Eastern Steppe was populated by hunter-gatherers of ANA and ANE ancestry during the mid-Holocene and then shifted to a dairy pastoralist economy during the Bronze Age. Migrating Yamnaya/Afanasievo steppe herders, equipped with carts and domestic livestock (Kovalev and Erdenebaatar, 2009), appear to have first introduced ruminant dairy pastoralism ca. 3000 BCE (Wilkin et al., 2020a) but surprisingly had little lasting genetic impact, unlike in Europe (Allentoft et al., 2015; Haak et al., 2015; Mathieson et al., 2015). By the MLBA, ruminant dairy pastoralism had been adopted by populations throughout the Eastern Steppe (Wilkin et al., 2020a), regardless of ancestry, and this subsistence has continued, with the additions of horse milking in the LBA and camel milking in the Mongol period (Wilkin et al., 2020a), to the present day (Bat-Oyun et al., 2015; Kindstedt and Ser-Od, 2019). Puzzlingly, however, there is no evidence of selection for lactase persistence over this 5,000-year history, despite the repeated introduction of this genetic trait by subsequent migrations of groups from the west. This suggests a different trajectory of lactose adaptation in Asia that to date remains unexplained.

During the MLBA, we observed the formation of a tripartite genetic structure on the Eastern Steppe, characterized by the continuation of pre-Bronze Age ANA ancestry in the east and a cline of genetic variation between pre-Bronze Age ANA-ANE ancestry in the north and increasing proportions of a new Sintashta-related WSH ancestry in the west. The Sintashta, a western forest steppe culture with genetic links to the European Corded Ware cultures (Mathieson et al., 2015), were masters of bronze metallurgy and chariotry (Anthony, 2010), and the appearance of this ancestry on the Eastern Steppe may be linked to the introduction of new (especially horse-related) technologies. DSKC sites in particular show widespread evidence for horse use in transport and perhaps even riding (Taylor et al., 2015), and genetic analysis has demonstrated a close link between these animals and the Sintashta chariot horses (Fages et al., 2019). The strong east-west genetic division among Bronze Age Eastern Steppe populations at this time was maintained for more than a millennium and through the end of the EIA, when the first clear evidence for widespread horseback riding appears (Drews, 2004) and the heightened mobility of some groups, notably the eastern Slab Grave culture (Honeychurch, 2015), began to disrupt this structure. Eventually, the three major ancestries met and mixed, and this was contemporaneous with the emergence of the Xiongnu empire. The Xiongnu are characterized by extreme levels of genetic heterogeneity and increased diversity as new and additional ancestries from China, Central Asia, and the Western Steppe (Sarmatian-related) rapidly entered the gene pool.

Genetic data for the subsequent Early Medieval period are relatively sparse and uneven, and few Xianbei or Rouran sites have yet been identified during the 400-year gap between the Xiongnu and Türkic periods. We observed high genetic heterogeneity and diversity during the Türkic and Uyghur periods, and following the collapse of the Uyghur empire, we documented a final major genetic shift during the late medieval period toward greater eastern Eurasian ancestry, which is consistent with historically documented expansions of Tungusic- (Jurchen) and Mongolic- (Khitan and Mongol) speaking groups from the northeast into the Eastern Steppe (Biran, 2012). We also observed that this East Asian-related ancestry was brought into the Late Medieval populations more by male than female ancestors. By the end of the Mongol period, the genetic makeup of the Eastern Steppe had dramatically changed, retaining little of the ANE ancestry that had been a prominent feature during its prehistory. Today, ANE ancestry survives in appreciable amounts only in isolated Siberian groups and among the indigenous peoples of the Americas (Jeong et al., 2019). The genetic profile of the historic Mongols is still reflected among contemporary Mongolians, suggesting a relative stability of this gene pool over the last ∼700 years.

Having documented key periods of genetic shifts in the Eastern steppe, future work may be able to explore whether these shifts are also linked to cultural and technological innovations and how these innovations may have influenced the political landscape. Integrating these findings with research on changes in horse technology and herding practices, as well as shifts in livestock traits and breeds, may prove particularly illuminating. This study represents the first large-scale paleogenomic investigation of the Eastern Eurasian Steppe, and it sheds light on the remarkably complex and dynamic genetic diversity of the region. Despite this progress, there is still a great need for further genetic research in central and eastern Eurasia, and particularly in northeastern China, the Tarim Basin, and the eastern Kazakh steppe, in order to fully reveal the population history of the Eurasian Steppe and its pivotal role in world prehistory.

## STAR★Methods

### Key Resources Table

**Table:** 

REAGENT or RESOURCE	SOURCE	IDENTIFIER
**Biological Samples**		

Human archaeological skeletal material	This study	ARG001(AT-765)
Human archaeological skeletal material	This study	ARG002(AT-764)
Human archaeological skeletal material	This study	ARG003(AT-763)
Human archaeological skeletal material	This study	AST001(AT-841)
Human archaeological skeletal material	This study	ATS001(AT-459)
Human archaeological skeletal material	This study	BAM001(AT-752)
Human archaeological skeletal material	This study	BAU001(AT-409)
Human archaeological skeletal material	This study	BAY001(AT-304)
Human archaeological skeletal material	This study	BAZ001(AT-846)
Human archaeological skeletal material	This study	BER002(AT-905)
Human archaeological skeletal material	This study	BIL001(AT-340)
Human archaeological skeletal material	This study	BOR001(AT-707)
Human archaeological skeletal material	This study	BRG001(AT-650)
Human archaeological skeletal material	This study	BRG002(AT-651)
Human archaeological skeletal material	This study	BRG004(AT-655)
Human archaeological skeletal material	This study	BRG005(AT-653)
Human archaeological skeletal material	This study	BRL001(AT-296)
Human archaeological skeletal material	This study	BRL002(AT-294)
Human archaeological skeletal material	This study	BRU001(AT-154)
Human archaeological skeletal material	This study	BTO001(AT-435)
Human archaeological skeletal material	This study	BUL001(AT-923)
Human archaeological skeletal material	This study	BUL002(AT-922)
Human archaeological skeletal material	This study	BUR001(AT-589)
Human archaeological skeletal material	This study	BUR002(AT-536)
Human archaeological skeletal material	This study	BUR003(AT-535)
Human archaeological skeletal material	This study	BUR004(AT-537)
Human archaeological skeletal material	This study	CHD001(AT-173)
Human archaeological skeletal material	This study	CHN001(AT-121)
Human archaeological skeletal material	This study	CHN003(AT-141)
Human archaeological skeletal material	This study	CHN004(AT-105)
Human archaeological skeletal material	This study	CHN006(AT-109)
Human archaeological skeletal material	This study	CHN007(AT-128)
Human archaeological skeletal material	This study	CHN008(AT-138)
Human archaeological skeletal material	This study	CHN010(AT-119)
Human archaeological skeletal material	This study	CHN012(AT-98)
Human archaeological skeletal material	This study	CHN014(AT-125)
Human archaeological skeletal material	This study	CHN015(AT-115)
Human archaeological skeletal material	This study	CHN016(AT-208)
Human archaeological skeletal material	This study	DAR001(AT-766)
Human archaeological skeletal material	This study	DAR002(AT-767)
Human archaeological skeletal material	This study	DAS001(AT-391)
Human archaeological skeletal material	This study	DEE001(AT-389)
Human archaeological skeletal material	This study	DEK001/SHR001(AT-755)
Human archaeological skeletal material	This study	DEL001(AT-530)
Human archaeological skeletal material	This study	DOL001(AT-370)
Human archaeological skeletal material	This study	DUU001(AT-605)
Human archaeological skeletal material	This study	DUU002(AT-407)
Human archaeological skeletal material	This study	EME002(AT-708)
Human archaeological skeletal material	This study	ERD001(AT-831)
Human archaeological skeletal material	This study	ERM001/ERM002/ERM003(DA-KG-1909-001)
Human archaeological skeletal material	This study	FNO001(2008, pogrebenie 3)
Human archaeological skeletal material	This study	FNO003(2008, pogrebenie 4, skeleton 2)
Human archaeological skeletal material	This study	FNO006(2007, pogrebenie 1, formerly pogrebenie 18, main individual)
Human archaeological skeletal material	This study	FNO007(1996, pogrebenie 11, kostyak 2)
Human archaeological skeletal material	This study	GAN002(AT-835)
Human archaeological skeletal material	This study	GTO001(AT-624)
Human archaeological skeletal material	This study	GUN002(AT-780)
Human archaeological skeletal material	This study	HUD001(AT-290)
Human archaeological skeletal material	This study	IAG001(AT-590B)
Human archaeological skeletal material	This study	IKU001(AT-772)
Human archaeological skeletal material	This study	IMA001(2006 Mogila 76)
Human archaeological skeletal material	This study	IMA002(2005 Mogila 75)
Human archaeological skeletal material	This study	IMA003(2005 Mogila 73)
Human archaeological skeletal material	This study	IMA004(2003 Mogila 70)
Human archaeological skeletal material	This study	IMA005(2007 Mogila 78)
Human archaeological skeletal material	This study	IMA006(2007 Mogila 77)
Human archaeological skeletal material	This study	IMA007(2007 Mogila 79)
Human archaeological skeletal material	This study	IMA008(2004 Mogila 71)
Human archaeological skeletal material	This study	JAA001(AT-910)
Human archaeological skeletal material	This study	JAG001(AT-878)
Human archaeological skeletal material	This study	KGK001(AT-900)
Human archaeological skeletal material	This study	KHI001(AT-398)
Human archaeological skeletal material	This study	KHL001(AT-363)
Human archaeological skeletal material	This study	KHN001/KHN002(AT-758; AT-759)
Human archaeological skeletal material	This study	KHO001(AT-354)
Human archaeological skeletal material	This study	KHO006(AT-361B)
Human archaeological skeletal material	This study	KHO007(AT-361A)
Human archaeological skeletal material	This study	KHU001(AT-861)
Human archaeological skeletal material	This study	KHV002(AT-811)
Human archaeological skeletal material	This study	KNN001(AT-754)
Human archaeological skeletal material	This study	KNU001(AT-352)
Human archaeological skeletal material	This study	KRN001(AT-643)
Human archaeological skeletal material	This study	KRN002(AT-644)
Human archaeological skeletal material	This study	KUM001(AT-628)
Human archaeological skeletal material	This study	KUR001(AT-635)
Human archaeological skeletal material	This study	MIT001(AT-975)
Human archaeological skeletal material	This study	MRI001(AT-800)
Human archaeological skeletal material	This study	NAI001(AT-149)
Human archaeological skeletal material	This study	NAI002/NAI003(AT-152)
Human archaeological skeletal material	This study	NOM001(AT-917)
Human archaeological skeletal material	This study	NRC001(AT-393)
Human archaeological skeletal material	This study	OLN001.A(AT-871)
Human archaeological skeletal material	This study	OLN001.B(AT-871)
Human archaeological skeletal material	This study	OLN002(AT-891)
Human archaeological skeletal material	This study	OLN003(AT-892)
Human archaeological skeletal material	This study	OLN004(AT-969)
Human archaeological skeletal material	This study	OLN005(AT-973)
Human archaeological skeletal material	This study	OLN007(AT-972)
Human archaeological skeletal material	This study	OLN008(AT-873)
Human archaeological skeletal material	This study	OLN009(AT-896)
Human archaeological skeletal material	This study	OLN010(AT-893)
Human archaeological skeletal material	This study	OLN011(AT-897)
Human archaeological skeletal material	This study	OLN012(AT-894)
Human archaeological skeletal material	This study	PTO001(Plitochnaya Mogila 4)
Human archaeological skeletal material	This study	RAH001(AT-532)
Human archaeological skeletal material	This study	SAN001(AT-575)
Human archaeological skeletal material	This study	SBG001(AT-960)
Human archaeological skeletal material	This study	SHA001(AT-594)
Human archaeological skeletal material	This study	SHG001(AT-701)
Human archaeological skeletal material	This study	SHG002(AT-699)
Human archaeological skeletal material	This study	SHG003(AT-703)
Human archaeological skeletal material	This study	SHT001(AT-26)
Human archaeological skeletal material	This study	SHT002(AT-25)
Human archaeological skeletal material	This study	SHU001(AT-233)
Human archaeological skeletal material	This study	SHU002(AT-232B)
Human archaeological skeletal material	This study	SKT001(CA-4-1)
Human archaeological skeletal material	This study	SKT002(CA-19)
Human archaeological skeletal material	This study	SKT003(CA-13-1)
Human archaeological skeletal material	This study	SKT004(CA-24)
Human archaeological skeletal material	This study	SKT005(CA-8)
Human archaeological skeletal material	This study	SKT006(CA-17)
Human archaeological skeletal material	This study	SKT007(CA-3-1)
Human archaeological skeletal material	This study	SKT008(CA-28)
Human archaeological skeletal material	This study	SKT009(CA-9-1)
Human archaeological skeletal material	This study	SKT010(CA-7)
Human archaeological skeletal material	This study	SKT012(CA-29)
Human archaeological skeletal material	This study	SOL001(AT-274)
Human archaeological skeletal material	This study	SON001(AT-150)
Human archaeological skeletal material	This study	SOU001(AT-501)
Human archaeological skeletal material	This study	TAH002(AT-360)
Human archaeological skeletal material	This study	TAK001(AT-401A)
Human archaeological skeletal material	This study	TAV001(AT-625/688)
Human archaeological skeletal material	This study	TAV005(AT-670/695)
Human archaeological skeletal material	This study	TAV006(AT-623)
Human archaeological skeletal material	This study	TAV011(AT-671/687)
Human archaeological skeletal material	This study	TEV002(AT-33)
Human archaeological skeletal material	This study	TEV003(AT-145)
Human archaeological skeletal material	This study	TMI001(AT-751)
Human archaeological skeletal material	This study	TSA001(AT-784)
Human archaeological skeletal material	This study	TSA002(AT-816)
Human archaeological skeletal material	This study	TSA003(AT-783)
Human archaeological skeletal material	This study	TSA004(AT-782)
Human archaeological skeletal material	This study	TSA005(AT-815)
Human archaeological skeletal material	This study	TSA006(AT-814)
Human archaeological skeletal material	This study	TSA007(AT-786)
Human archaeological skeletal material	This study	TSB001(AT-804)
Human archaeological skeletal material	This study	TSI001(AT-802)
Human archaeological skeletal material	This study	TUH001(AT-543)
Human archaeological skeletal material	This study	TUH002(AT-542)
Human archaeological skeletal material	This study	TUK001/TAV008(AT-729;AT-728)
Human archaeological skeletal material	This study	TUK002(AT-757)
Human archaeological skeletal material	This study	TUK003(AT-684)
Human archaeological skeletal material	This study	TUM001(AT-913)
Human archaeological skeletal material	This study	UAA001(AT-614)
Human archaeological skeletal material	This study	UGO001(AT-588)
Human archaeological skeletal material	This study	UGO002(AT-581)
Human archaeological skeletal material	This study	UGU001(AT-749)
Human archaeological skeletal material	This study	UGU002(AT-549)
Human archaeological skeletal material	This study	UGU003(AT-570)
Human archaeological skeletal material	This study	UGU004(AT-805)
Human archaeological skeletal material	This study	UGU005(AT-747)
Human archaeological skeletal material	This study	UGU006(AT-692)
Human archaeological skeletal material	This study	UGU010(AT-690)
Human archaeological skeletal material	This study	UGU011(AT-748)
Human archaeological skeletal material	This study	ULA001(AT-840)
Human archaeological skeletal material	This study	ULI001(AT-676)
Human archaeological skeletal material	This study	ULI002(AT-675)
Human archaeological skeletal material	This study	ULI003(AT-680)
Human archaeological skeletal material	This study	ULN001(AT-823)
Human archaeological skeletal material	This study	ULN002(AT-920)
Human archaeological skeletal material	This study	ULN003(AT-921)
Human archaeological skeletal material	This study	ULN004(AT-885)
Human archaeological skeletal material	This study	ULN005(AT-769)
Human archaeological skeletal material	This study	ULN006(AT-962)
Human archaeological skeletal material	This study	ULN007(AT-883)
Human archaeological skeletal material	This study	ULN009(AT-884)
Human archaeological skeletal material	This study	ULN010(AT-964)
Human archaeological skeletal material	This study	ULN011(AT-882)
Human archaeological skeletal material	This study	ULN015(AT-824)
Human archaeological skeletal material	This study	ULZ001(AT-674)
Human archaeological skeletal material	This study	UUS001(AT-613)
Human archaeological skeletal material	This study	UUS002(AT-610)
Human archaeological skeletal material	This study	UVG001(AT-338)
Human archaeological skeletal material	This study	YAG001(AT-590A)
Human archaeological skeletal material	This study	YUR001(AT-649)
Human archaeological skeletal material	This study	ZAA001(AT-954)
Human archaeological skeletal material	This study	ZAA002(AT-957)
Human archaeological skeletal material	This study	ZAA003(AT-953)
Human archaeological skeletal material	This study	ZAA004(AT-959)
Human archaeological skeletal material	This study	ZAA005(AT-956)
Human archaeological skeletal material	This study	ZAA007(AT-958)
Human archaeological skeletal material	This study	ZAM001(AT-390)
Human archaeological skeletal material	This study	ZAM002(AT-711)
Human archaeological skeletal material	This study	ZAR002(AT-271)
Human archaeological skeletal material	This study	ZAY001(AT-768)

**Chemicals, Peptides, and Recombinant Proteins**		

USER™ Enzyme, recombinant	NEB	M5508

**Critical Commercial Assays**		

HiSeq® 3000/4000 SR Cluster Kit	Illumina	PE-410-1001
HiSeq® 3000/4000 PE Cluster Kit	Illumina	GD-410-1001
HiSeq® 3000/4000 SBS Kit (50 cycles)	Illumina	FC-410-1001
HiSeq® 3000/4000 SBS Kit (150 cycles)	Illumina	FC-410-1002

**Deposited Data**		

Raw and analyzed data	This study	ENA: PRJEB35748
Haploid genotype data for 1240K panel (Edmond Data Repository of the Max Planck Society)	This study	https://edmond.mpdl.mpg.de/imeji/collection/2ZJSw35ZTTa18jEo

**Software and Algorithms**		

EAGER v1.92.55	(Peltzer et al., 2016)	https://github.com/apeltzer/EAGER-GUI
AdapterRemoval v2.2.20	(Schubert et al., 2016)	https://github.com/MikkelSchubert/adapterremoval
BWA v0.7.12	(Li and Durbin, 2009)	http://bio-bwa.sourceforge.net
dedup v0.12.2	(Peltzer et al., 2016)	https://github.com/apeltzer/DeDup
bamUtils v.1.0.13	(Jun et al., 2015)	https://github.com/statgen/bamUtil
samtools mpileup	(Li and Durbin, 2009)	http://www.htslib.org/doc/samtools.html
pilupCaller v1.2.2	(https://github.com/stschiff/sequenceTools)	https://github.com/stschiff/sequenceTools
mapDamage v2.0.6	(Jónsson et al., 2013)	https://github.com/MikkelSchubert/mapDamage
Schmutzi	(Renaud et al., 2015)	https://github.com/grenaud/schmutzi
circularmapper v1.1	(Peltzer et al., 2016)	https://github.com/apeltzer/CircularMapper
ANGSD v0.910	(Korneliussen et al., 2014)	http://www.popgen.dk/angsd/index.php/ANGSD
HaploGrep 2 v2.1.19	(Weissensteiner et al., 2016)	https://haplogrep.i-med.ac.at/category/haplogrep2/
yHaplo	(Poznik, 2016)	https://github.com/alexhbnr/yhaplo
Eigensoft v7.2.1	(Patterson et al., 2006)	https://github.com/DReichLab/EIG
DATES	(Narasimhan et al., 2019)	https://github.com/priyamoorjani/DATES
admixtools v5.1	(Patterson et al., 2012)	https://github.com/DReichLab/AdmixTools

### Resource Availability

#### Lead Contact

Further information and requests for resources should be directed to and will be fulfilled by the Lead Contact, Christina Warinner (warinner@fas.harvard.edu).

#### Materials Availability

This study did not generate new unique reagents.

#### Data and Code Availability

The accession number for all newly reported sequencing data reported in this paper are available from the European Nucleotide Archive: PRJEB35748. 1240K genotype data are available on the Edmond Max Planck Data Repository under the link: https://edmond.mpdl.mpg.de/imeji/collection/2ZJSw35ZTTa18jEo.

### Experimental Model and Subject Details

Here we present new genome-wide data for 213 ancient individuals from Mongolia and 13 individuals from Buryatia, Russia, which we analyze together with 21 previously published ancient Mongolian individuals (Jeong et al., 2018), for a total of 247 individuals. Human remains analyzed in this study were reviewed and approved by the Mongolian Ministry of Culture and the Mongolian Ministry of Education, Culture, Science, and Sport under reference numbers A0122772 MN DE 0 8124, A0109258 MN DE 7 643, and A0117901 MN DE 9 4314, and declaration number 12-2091008-20E00225. All new Mongolian individuals, except ERM, were sampled from the physical anthropology collections at the National University of Mongolia and the Institute for Archaeology and Ethnology in Ulaanbaatar, Mongolia. ERM001/002/003 was provided by Jan Bemmann. Russian samples were collected from the Institute for Mongolian, Buddhist, and Tibetan Research as well as the Buryat Scientific Center, Russian Academy of Sciences (RAS).

Together, this ancient Eastern Steppe dataset of 247 individuals originates from 89 archaeological sites (Figure 1; Figure S1A; Table S1A) and spans approximately 6,000 years of time (Tables S1A, S1B, and S2C). High quality genetic data was successfully generated for 214 individuals and was used for population genetic analysis (Table S2A). Subsistence information inferred from proteomic analysis of dental calculus has been recently published for a subset of these individuals (n = 32; Wilkin et al., 2020a), and stable isotope analysis of bone collagen and enamel (n = 137) is also in progress (Wilkin et al., 2020b); together, these data allow direct comparison between the biological ancestry of specific archaeological cultures and their diets, particularly with respect to their dairy and millet consumption. Below, we provide an overview of the geography and ecology of the archaeological sites in this study, as well as their temporal and cultural context.

#### Geography and ecology of Mongolia

Mongolia is located in Inner Asia between Russia and China, and it encompasses most of the Eurasian Eastern Steppe (Figure 1; Figure S1A). Mongolia has 21 aimags (provinces) and can be divided into ten geographic regions (Figure S1B) with distinct ecological (Figure S1C) and cultural features (Taylor et al., 2019). For example, far north Mongolia borders Siberia and includes both high mountain and mountain-taiga ecological zones, and it is the only aimag where reindeer pastoralism is practiced. North Mongolia is dominated by forest-steppe, but also contains mountain-taiga and steppe zones; cattle and yak pastoralism is particularly productive here, and Bulgan province is renowned for its horse pastoralism. The Altai region represents an extension of the Altai mountains from Russia into Mongolia and consists of a patchwork of environments including high mountains, valleys, and lakes, and ranging from forest steppe to desert steppe as the region stretches from north to south; pastoral economy in the Altai is mixed and differs by local environmental conditions. South Mongolia is dominated by the Gobi Desert, and it borders central and southeast Mongolia, which are largely characterized by desert-steppe; camel pastoralism is found throughout these regions. East Mongolia is a large expansive steppe zone that stretches to northeastern China. Today, mining is important in eastern Mongolia, as well as cattle, sheep, goat, and horse pastoralism.

#### Overview of Mongolian archaeology

Mongolian prehistory extends back more than 40,000 years, with documented sites ranging from the Upper Paleolithic to the present day. During nearly all of this time, lifeways in Mongolia have been nomadic, either supported by hunting, fishing and gathering or by pastoralism. The short-term and ephemeral nature of nomadic camp sites makes them difficult to identify on the landscape, and wind deflation has further reduced the visibility and preservation of many domestic sites. Only during the Bronze Age, with the sudden appearance of stone mounds and other burial features, do sites become more conspicuous and the archaeology better attested. As such, knowledge of Mongolian prehistory is strongly biased toward the past five millennia. The archaeology of Mongolia can be divided into 7 main periods: (1) pre-Bronze Age, prior to 3500 BCE; (2) Early Bronze Age, 3500-1900 BCE; (3) Middle/Late Bronze Age, 1900-900 BCE; (4) Early Iron Age, 900-300 BCE; (5) Xiongnu, 200 BCE to 100 CE; (6) Early Medieval, 100-850 CE, and (7) Late Medieval, 850-1650 CE. A brief summary of each period, as well as details for the sites included in this study, are provided below, in Figure S1, and in Table S1.

##### Pre-Bronze Age (prior to 3500 BCE)

The early archaeological record of Mongolia is poorly understood, particularly with respect to human remains and burials. While occasional finds provide direct evidence of anatomically modern humans in Mongolia as far back as the Early Upper Paleolithic (Devièse et al., 2019; Zwyns et al., 2019), only a small handful of intentionally buried skeletons have been recovered prior to the end of the 4th millennium BCE. Although early and middle Holocene-era (10,000-3500 BCE) features and burials have been referred to as “Mesolithic,” “Neolithic,” or “Eneolithic” (Hanks, 2010), there is no direct evidence for domestic animals or a food-producing economy at any of these localities, although pottery was in wide use by the mid-Holocene (Janz et al., 2017). Pre-Bronze Age burials in eastern Mongolia are characterized by an absence of surficial construction features, while in northern Mongolia pre-Bronze burials typically consist of small stone cairns. The burial goods of this period include artifacts made from stone, mother-of-pearl, and animal bones, such as deer and marmot (Eregzen, 2016). Two individuals in this study date to this pre-Bronze Age period in Mongolia. The first, dating to ca. 4600 BCE, is from Kherlengiin Ereg (SOU), located on the south bank of the Kherlen River near Choibalsan city at the extreme eastern end of Mongolia in Dornod province (Dorj, 1969). It was found in a disturbed context, and the original burial position could not be reconstructed. However, other burials from this period and region are typically crouched (https://edmond.mpdl.mpg.de/imeji/collection/2ZJSw35ZTTa18jEo). The second, dating to ca. 3700 BCE, is from Erdenemandal (ERM) in the Arkhangai province of north Mongolia (https://edmond.mpdl.mpg.de/imeji/collection/2ZJSw35ZTTa18jEo). It lacked stone construction features and consisted of a simple crouched pit burial beneath a shallow earthen mound. The burial was recovered at a depth of 2.3 m and the grave mound appears to be part of a larger cemetery.

In addition to these two pre-Bronze burials from Mongolia, we also analyzed four pre-Bronze individuals from the site of Fofonovo (FNO) near Lake Baikal in Buryatia, Russia (Lbova et al., 2008). All were buried in simple pit burials partially flexed (only their legs bent) as individuals, or sometimes several persons together. People at Fofonovo, like many others around Lake Baikal, were interred with many burial goods, including an array of bone and stone beads, neck pieces, chipped stone blades and points, bone harpoons, and pottery. Among these items were fragments or worked ornaments from wild boar, sable, and hawk.

Although pre-Bronze Age material from Mongolia is sparse, recent excavations in neighboring regions provide important context. In southern Russia, excavations by the Baikal Archaeological Project (BAP) at three sites (Lokomotiv, Shamanka II, and Ust’-Ida I) have enabled characterization of the Lake Baikal Neolithic Kitoi (5200-4200 BCE) and Isakovo (4000-3000 BCE) mortuary traditions, including genome sequencing of 14 of these hunter-gatherers (Damgaard et al., 2018a) (Baikal_EN; also characterized in later studies as East Siberian Hunter Gatherers, ESHG) (Narasimhan et al., 2019). The genomes of six hunter-gatherers dating to ca. 5700 BCE are also available from the site of Devil’s Gate (Sikora et al., 2019; Siska et al., 2017), a Neolithic cave site on the border between Russia and Korea. These individuals, separated by 2500 km, share a similar ancestry to each other and to modern Tungusic speakers in the lower Amur Basin, who we refer to as Ancient Northeast Asians (ANA).

##### Early Bronze Age (ca. 3500-1900 BCE)

Two major cultural phenomena associated with monumental mortuary architecture have been described in Mongolia during the Early Bronze Age (EBA): Afanasievo and Chemurchek. Both exhibit features linking them to ruminant pastoralism and to cultures further west.

*Afanasievo (3150-2750 BCE).* Beginning ca. 3150 BCE and persisting until ca. 2750 BCE (Taylor et al., 2019), although perhaps as late as 2600 BCE, stone burials belonging to the Afanasievo culture type have been recovered from the Khangai Mountains in central Mongolia and the Altai Mountains of western Mongolia. These burials contain the earliest direct evidence for domestic livestock (sheep/goat and cattle) in Mongolia. Afanasievo burials in Mongolia typically consist of circular flat stones bounded by upright stones, which overlay an internal burial pit containing an extended individual with flexed legs. Such burials are similar to Afanasievo kurgans in the Russian Altai (Vadetskaya et al., 2014), which contain burials of supine individuals with flexed legs and heads typically facing east. The burial mounds at Khuurai Gobi 1 and Ulaankhus (Bayan-Ulgii province, western Mongolia; not sampled in this study) exhibit typical Afanasievo architectural features (https://edmond.mpdl.mpg.de/imeji/collection/2ZJSw35ZTTa18jEo).

In addition to domestic animal remains, Afanasievo burial mounds contain egg-shaped pottery vessels, and sometimes include metal artifacts (from copper, gold, and silver) and apparent deconstructed cart objects (Kovalev and Erdenebaatar, 2009). Recent analysis of proteins in human dental calculus from Afanasievo burials directly demonstrates the utilization of ruminant dairy products and the presence of domestic animals in the Afanasievo economy (Wilkin et al., 2020a).

We analyzed individuals from one Afanasievo site in this study: Shatar Chuluu (SHT). Located in Byankhongor province on the south slope of the Khangai mountains, Shatatar Chuulu is the easternmost known Afanasievo cemetery in Eurasia. Three of the site’s burial mounds have been excavated, and each consisted of a flat platform of round stones bounded by large boulders. The burials were arranged in pits beneath the mounds, and the bodies were laid out in a supine position with flexed knees and heads facing to the west. Despite the fact that few burial goods were found, the overall architectural design of the mounds combined with isolated fragments of typical Afanasievo vessels make it possible to attribute these mounds to the Afanasievo archaeological culture.

*Chemurchek (2750-1900 BCE).* The Chemurchek archaeological culture (also called Hemtseg, Qiemu’erqieke, Shamirshak), spans the period between 2750 BCE-1900 BCE (Taylor et al., 2019). These features are found in western Mongolia and adjoining regions of bordering countries, including the Dzungarian Basin of Xinjiang and eastern Kazakhstan (Honeychurch, 2017; Kovalev and Erdenebaatar, 2009). Chemurchek mortuary architecture is characterized by collective burials in large stone cists surrounded by stone and earthen cairns overlapping one another or by large rectangular stone fences up to 50 m in length. The Chemurcheck burial at Kheviin Am (Khovd province, western Mongolia; not sampled in this study) exhibits typical Chemurchek features (https://edmond.mpdl.mpg.de/imeji/collection/2ZJSw35ZTTa18jEo). Similar to Afanasievo burials, individuals found in Chemurchek tombs are laid out in a supine position with flexed legs. Adjacent to many Chemurchek burial features along the eastern side are anthropomorphic standing stones, sometimes depicted holding a shepherd’s crook (https://edmond.mpdl.mpg.de/imeji/collection/2ZJSw35ZTTa18jEo). Inside the burials, artifacts such as stone bowls, bone tools, ceramics, and sometimes metal jewelry, have been recovered. Occasionally, non-funerary ritual structures, such as fences containing earthen pits with charcoal and animal remains or large stone fences depicting petroglyphs, are also attributed to this culture (Kovalev, 2014). Recent analysis of proteins in human dental calculus from these features confirmed the utilization of ruminant dairy products and the presence of domestic animals in the Chemurchek economy (Wilkin et al., 2020a), although available radiocarbon chronology appears to preclude a meaningful exploitation of domestic horses (Taylor et al., 2019). We analyzed individuals from three Chemurchek sites in this study: Yagshiin Khuduu (IAG/YAG), Khundii Gobi (KUM), and Khuurai Gobi 2 (KUR). Yagshiin Khuduu is located in the southern Mongolian Altai, while Khundii Gobi and Khuurai Gobi 2 are located in the northern Mongolian Altai. Whereas Yagshiin Khuduu represents a typical Chemurchek burial within a stone cist, the two northern Chemurchek mortuary sites consist of burials within rectangular mounds bounded by upright stones and may belong to a “mixed type” incorporating local traditions from eastern Kazakhstan and the Russian Altai.

*Unclassified.* In addition to these two main types, we also analyzed one individual from a site with an uncertain burial type: Denj (GUR).

Comparative genomic data are available for several contemporaneous sites in neighboring regions, including: (1) Botai, a horse hunter-herder site dating to ca. 3500 BCE in northern Kazakhstan (Damgaard et al., 2018a); (2) multiple sites of Afanasievo ruminant pastoralists dating to ca. 3000-2500 BCE in the Kazakh and Russian Altai-Sayan region (Allentoft et al., 2015; Narasimhan et al., 2019); (3) Dali, a site in southeastern Kazakhstan whose lowest layers contain a woman dating to ca. 2650 BCE but lacking burial context (Narasimhan et al., 2019); (4) Gonur Tepe, a representative Bactria-Margiana Archaeological Complex (BMAC) site in Turkmenistan dating to 2300-1600 BCE (Narasimhan et al., 2019); and (5) three Lake Baikal sites, Ust’-Ida I, Shamanka II and Kurma Xi, associated with the Glazkovo mortuary tradition and dating to ca. 2200-1800 BCE (Damgaard et al., 2018a; 2018b).

##### Middle/Late Bronze Age (ca. 1900-900 BCE)

The Middle/Late Bronze Age (MLBA) in Mongolia is characterized by the sudden and widespread appearance of monumental mortuary architecture across Mongolia. Primarily taking the form of stone mounds, but also including stone stelae and other features, these Middle and Late Bronze Age structures remain among the most conspicuous features on the landscape even today. Middle and Late Bronze Age burial mound typology is complex and there is scholarly debate and disagreement on how to precisely define and delineate different mortuary types. In this study, we focused on several main burial forms: Mönkhkhairkhan, Baitag, Deer Stone-Khirigsuur Complex (DSKC), Ulaanzuukh, and Tevsh (Shape). We provide a general overview of these burial types, but acknowledge that not all scholars will agree with all details.

*Mönkhkhairkhan (1850-1350 BCE).* Dating to after the Chemurchek period, ca. 1850-1350 cal. BCE (Taylor et al., 2019), Mönkhkhairkhan burials are found across northern and western areas of Mongolia and in Tuva, spanning a geographic area approximately 1000 km from west to east and 500 km from north to south. Mönkhkhairkhan burials are characterized by a crouched/flexed burial position, and graves are completely filled in with stones after burial, as seen at the site Ulaan Goviin Uzuur 2 (https://edmond.mpdl.mpg.de/imeji/collection/2ZJSw35ZTTa18jEo). Overlaying the burials are external stone structures consisting of flat round or rectangular platforms. Such barrows are typically 3-5 m in diameter, but occasionally reach up to 40 m in diameter. Ritual structures may include stone circles and stelae. No pottery or animal bones have been reported from within Mönkhkhairkhan burials, and little is known of its economy. Burial goods include tin bronze knives and awls, tin bronze two-trumpet shaped rings, bone spoons, bone arrowheads, and ornaments made of bone, shell, and stone (Clark, 2015; Eregzen, 2016). Knives and rings have analogs both in western Siberia and among the Oijia, Lower Xiajiadian, Siba, and Erlitou cultures of China. Similarities between the grave goods and funerary practices of this culture with those at sites to the west of Lake Baikal (Cis-Baikal) have been previously noted (Erdenebaatar, 2016; Kovalev, 2017; Kovalev and Erdenebaatar, 2009). We analyzed individuals from two Mönkhkhairkhan sites in this study: Khukh Khoshuunii Boom (KHU) and Ulaan Goviin Uzuur 2 (UAA).

*Baitag (1050-900 BCE).* Restricted to southwestern Mongolia, Baitag burials consist of non-mounded, small stone rings constructed from a single layer of small flat stone slabs, as seen for example at the site of Uyench (Khovd province, western Mongolia; not analyzed in this study, https://edmond.mpdl.mpg.de/imeji/collection/2ZJSw35ZTTa18jEo) (Kovalev and Erdenebaatar, 2009). A central burial pit oriented west-east contains a single individual oriented in a supine position with knees up. Unlike DSKC burials but similar to preceding Altai groups, such as the Mönkhkhairkhan and Chemurchek, the Baitag burials contain various small grave goods, including bronze jewelry. These artifacts share similarities with those included in Karasuk culture graves from the Minusinsk Basin, as well as in burials in Xinjiang and Gansu (Sibu culture) in northwestern China (Kovalev and Erdenebaatar, 2009). In this study, we analyzed one Baitag individual (ULI004) from the site of Uliastai River (ULI), middle terrace.

*Deer Stone-Khirigsuur Complex (DSKC) (1350-900 BCE).* This culture comprises three different monumental features - *khirigsuurs*, deer stones, and Sagsai-style graves - and is tightly associated with the emergence of horsemanship in the Mongolian Steppe during the late second millennium BCE. In general, DSKC sites are concentrated in the western, northern, and central parts of Mongolia, with only a small number of sites further east (Honeychurch, 2015). *Khirigsuurs* in central Mongolia consist of large stone mounds, surrounded by an outer fence that is either circular or four-cornered in shape, as seen for example at the site of Egiin Gol (Bulgan province, northern Mongolia; not analyzed in this study, https://edmond.mpdl.mpg.de/imeji/collection/2ZJSw35ZTTa18jEo). In the Mongolian Altai, some *khirigsuurs* display stone lines between the central cairn and outer fence, producing a shape that resembles a spoked wheel. Although their exclusive function as burials is a subject of contention (Wright, 2012), *khirigsuurs* often contain a supine human body (Littleton et al., 2012) and do not typically yield other kinds of artifacts. Deer stones are anthropomorphic standing stones found either independently or co-occurring with *khirigsuurs*. Deriving their name from the common motif of stylized deer, carvings on these stelae also depict belts, weapons, and tools - and occasionally even a human face. Many of the weapons depicted on deer stones are of recognizably Karasuk style, bearing a strong resemblance to bronzes found in tombs in the Minusinsk Basin more than 500 km to the northwest (Honeychurch, 2015), and the presence of deer stones in nearby Tuva further support the possibility of long-distance interaction between the Karasuk and the DSKC (Honeychurch, 2015). At many Mongolian *khirigsuurs* and deer stones (particularly in central Mongolia), smaller stone mounds containing the head, jaw, neck, and hooves of individual horses are found surrounding the eastern perimeter of the monument (https://edmond.mpdl.mpg.de/imeji/collection/2ZJSw35ZTTa18jEo). These horse mounds can range in number from a handful into the hundreds or thousands. Osteological study of DSKC horses reveal their use in transport and likely riding, as well as their sophisticated management as herd animals (Taylor et al., 2015; 2018). Another kind of satellite feature found at DSKC sites, open stone circles, often yield partial remains of sheep, goat, or cattle. Analogies in the composition and architecture of deer stone and *khirigsuur* sites with horse sacrifices has led some to interpret deer stones as cenotaphs for people not buried in funerary structures (Kovalev et al., 2014; 2016).

Sagsai-style graves (Taylor et al., 2019; Törbat, 2016) are often associated with the DSKC culture; however, this grave type is not associated with horse sacrifice. Sagsai burials consist of round or rectangular stone platforms without an outer fence, but with large boulders demarcating the edge of the cairn. Beneath the center of the platform, individual burials are positioned within stone cists or narrow pits covered by stone slabs. Individuals are typically arranged in a supine position without burial goods. Alternative names that have been used to describe this burial style include Munguntaiga, Mongun-Taiga, and even *khirigsuur*. A similar burial style known as a “slope burial” due to its common occurrence on the edge of hillslopes is often considered a variant of the Sagsai type. Slope burials consist of a similar stone cairn with four-corner fences and upright corner posts. Sagai-style mounds and slope burials are concentrated in western and northern Mongolia, and representative examples have been excavated in Khövsgöl province (https://edmond.mpdl.mpg.de/imeji/collection/2ZJSw35ZTTa18jEo).

Radiocarbon modeling dates central Mongolian *khirigsuurs* to between ca. 1350-900 cal BCE, deer stones to ca. 1150-750 cal. BCE, and places the emergence of DSKC horse ritual at ca. 1200 cal BCE (Taylor et al., 2019, 2017). Sagsai-style graves fall within this range (1350-1050 BCE), further strengthening the claim for their affiliation to the DSKC culture sphere (Taylor et al., 2019). It should be noted, however, that these dating estimates could be influenced by taphonomic or dietary processes. In particular, young dates for deer stones may be influenced by radiocarbon contamination (Zazzo et al., 2019), and early dates may be influenced by aquatic reservoir effects. Dairy proteins preserved in dental calculus demonstrate a pastoral, ruminant dairy-based economy at *khirigsuur* and Sagsai sites (Jeong et al., 2018; Wilkin et al., 2020a), and one Sagsai site to date has also yielded evidence of horse milking (Wilkin et al., 2020a). Perhaps buoyed by the innovation or adoption of mounted horseback riding and accompanying changes to the pastoral economy, deer stones and various stone cairns with external fences proliferated over an extremely wide geographic range, reaching modern-day Tuva and southern Russia, Kazakhstan, Kyrgyzstan, and northwest China. We analyzed individuals from four DSKC sites in this study: Arbulag Soum (ARS), Berkh Mountain (BER), Uliastai River Lower Terrace (ULI), and Uushigiin Uver (UUS).

*Ulaanzuukh - Tevsh (Shape) (1450-1150 BCE).* Beginning in the mid-second millennium BCE, a number of different burial traditions emerged in the southern and southeastern regions of Mongolia. United by a common prone or face-down burial position, these groups were once considered part of the Slab Grave culture, but are now either classified separately as discrete burial types Ulaanzuukh and Tevsh/Shape (Kovalev and Erdenebaatar, 2009) or are sometimes considered a single cultural unit (Ulaanzuukh-Tevsh/Shape) (Honeychurch, 2015). Ulaanzuukh burials (named after the type site), are found within southeast Mongolia and consist of non-mounded square or rectangular platforms surrounded by a wall of upright slabs or layered stone placed over a central burial pit (Dashtseveg et al., 2014). The site of Adgiin Gol (Sukhbaatar province, eastern Mongolia; not analyzed in this study) provides a representative example of this burial type (https://edmond.mpdl.mpg.de/imeji/collection/2ZJSw35ZTTa18jEo). Tevsh burials, also called Shape burials, are found throughout southern Mongolia and central Inner Mongolia and are similar to Ulaanzuukh burials except that they are hourglass-shaped (https://edmond.mpdl.mpg.de/imeji/collection/2ZJSw35ZTTa18jEo). The walls of Tevsh/Shape burials are typically made of layered stone (masonry), and sometimes with a single edge ringed with upright slabs. Other burial styles in the region, which may represent variant types, include D-shaped and stirrup-shaped burial structures.

Radiocarbon modeling suggests that Ulaanzuukh features date to ca. 1450-1150 BCE, while shape burials could both predate and postdate this mark – although very few have been reliably dated (Taylor et al., 2019). Burials of this culture often contain apparently domestic livestock remains, including sheep, goat, horse, and cattle (Nelson et al., 2009), although the earliest horses from these features date to only ca. 1250 BCE (Taylor et al., 2017). Recent analysis of proteins in human dental calculus has confirmed the utilization of ruminant dairy products and the presence of domestic animals in the Ulaanzuukh economy (Wilkin et al., 2020a). A few bronze knives of Karasuk origin have been found in Ulaanzuukh-Tevsh graves, indicating possible long-distance connections to the Minusinsk basin (Honeychurch, 2015). We analyzed individuals from two Ulaanzuukh sites in this study: Bulgiin Ekh (BUL) and Ulaanzuukh (ULN). We did not analyze individuals from Tevsh/Shape burials in this study.

*Unclassified.* In addition to these main types, we also analyzed individuals from six Late Bronze Age sites containing burials with uncertain or unclassified cultural affiliations: Biluutiin Am (BIL), Khoit Tsenkher (KHI/KHO), Shar Gobi 3 (SBG), Tsaidam Bag (TSB/TSI), Uliastai River lower terrace (ULI), and Uliastai Zastav II (ULZ). For more information on these burials, see Table S1C.

Comparative genomic data are available for several contemporaneous archaeological sites in neighboring regions, including: (1) four Okunevo sites (Verkhni Askiz, Okunev Ulus, Uybat, Syda 5), dating to 2200-2600 BCE (Allentoft et al., 2015; Damgaard et al., 2018a); (2) five Sintashta sites (Bulanovo, Tanabergen II, Stepnoe VII, and Bol’shekaraganskii, Kamennyi Ambar 5 cemetery), dating to ca. 2200-1700 BCE (Allentoft et al., 2015; Narasimhan et al., 2019); (3) four Central Steppe sites near Krasnoyarsk in western Siberia (Krasnoyarsk Krai, Potroshilovo II, Ust-Bir IV, Chumyash-Perekat-1) dating to 1700-1400 BCE (Narasimhan et al., 2019); (4) three Karasuk sites (Arban I, Sabinka II, and Bystrovka), dating to ca. 1400-1300 BCE (Allentoft et al., 2015).

##### Early Iron Age (ca. 900-300 BCE)

The Early Iron Age cultures of Inner Asia arose during a time of new technological advancements, including the development of composite bows and the beginnings of iron metallurgy used for items like arrows and horse-riding equipment (Honeychurch, 2015). These cultures include (1) the widespread Slab Grave culture, prevalent in eastern, southeastern, and central Mongolia as well as East Baikal and parts of northern China, and (2) the Sagly/Uyuk and Pazyryk cultures in the Sayan-Altai and portions of northwestern Mongolia. These latter cultures were part of a broader “Scythian” cultural phenomenon that spread into eastern Kazakhstan and across the Eurasian steppes, and which was related to Saka groups of northern Iran and the Tian Shan mountains. The Saka were an Iranian group broadly associated with the Scythians. Their later (after 200 BCE) military activities in Sogdia, Bactria, and the Tian Shan were recorded by Persian, Greek, and Chinese sources (Beckwith, 2009). Alongside the technological advancements of the Early Iron Age came increased long-distance interactions and the intensification of grain subsistence outside of the central Mongolian Steppe, but not yet by groups like the Slab Grave culture within Mongolia (Ventresca Miller and Makarewicz, 2019).

*Slab Grave (1000-300 BCE).* Beginning around 1000 BCE, a new burial style known as Slab Grave began appearing in eastern Mongolia. Slab graves are so called because of the large stone slabs used to mark the surface of the burial and to contain the rectangular burial space (hence in Mongolian they are called “square burials”) wherein single individuals are interred (Tsybiktarov, 1998). Although occasionally found singly, Slab Grave burials are more typically grouped into small cemeteries (Honeychurch, 2015). Stone slabs are set upright in the ground, and are thus prominent grave markers (https://edmond.mpdl.mpg.de/imeji/collection/2ZJSw35ZTTa18jEo). The burial pits are quite shallow, and human remains are rarely found complete or in good preservation. Over time, the Slab Grave culture expands northward into eastern Baikal and westward into central Mongolia, where it intrudes into former DSKC territory. Some slab graves tear apart the stone structures of *khirigsuurs* to construct the graves, and some even reuse deer stones for standing corner stones or laid-down slabs within the burial pit (Honeychurch, 2015). Unlike earlier Bronze Age burials, grave goods become more common in Slab Grave burials, consisting primarily of bronze beads, buttons, and small ornaments, as well as horse gear, arrowheads, axes, and knives. Stone, ceramic, and bone artifacts are also found in slab graves, and a few burials contained tripod-shaped pottery similar to those from Inner Mongolia and Manchuria or other non-local grave goods such as turquoise and carnelian beads from Central or South Asia (Honeychurch, 2015). Portions of livestock are often set at the edge or just outside of the rectangular burial space. In addition to faunal remains demonstrating the presence of domestic animals in the Slab Grave economy, recent analysis of proteins in human dental calculus has confirmed the utilization of ruminant and horse dairy products (Wilkin et al., 2020a). Although the Slab Grave phenomenon emerges out of the former territory of the Ulaanzuuk culture, archaeological evidence for the relationship between these two groups has been ambiguous. Nevertheless, the similarity of bronze artifacts, especially relating to horse gear and weaponry, found at Slab Grave sites to similar artifacts found in the Altai, Tuva, and Minusinsk regions may indicate a continuation of previously established long-distance relationships between these regions (Honeychurch, 2015). We analyzed individuals from five Slab Grave sites in this study: Bor Bulag (BOR), Morin Tolgoi (MIT), Darsagt (DAR), Shunkhlai Mountain (SHU), and Pesterevo 82 (PTO).

*Sagly/Uyuk (500-200 BCE).* This Early Iron Age culture centered in the Upper Yenisei River area, in modern-day Tuva, with some extensions into northwestern Mongolia (Murphy, 2003; Savinov, 2002). This culture is also referred to as the Sagly-Bazhy culture, and is best known in Mongolia by the thoroughly excavated site of Chandman Mountain (Ulaangom cemetery) included in this study (Novgorodova et al., 1982; Tseveendorj, 1980) (https://edmond.mpdl.mpg.de/imeji/collection/2ZJSw35ZTTa18jEo). Graves were marked by a round pile of stones and are often found in cemeteries of one to two dozen graves. Beneath the stone mounds are large log chambers containing several individuals (often assumed to be kin as they include men, women and children) all laid in partially flexed positions on their sides. Portions of sheep are also often placed in the graves. The Sagly/Uyuk log chambers resemble similar log architecture constructed by the contemporaneous Pazyryk culture in the Russian Altai and surrounding areas, and both the Sagly/Uyuk and Pazryk have been associated with the broader Saka culture (Parzinger, 2006). Similar to Slab Graves, recent analysis of proteins in human dental calculus has confirmed the utilization of ruminant and horse milk among those at Chandman Mountain (Wilkin et al., 2020a). Isotopic studies have also shown that some Uyuk communities, including at Chandman Mountain, had a significant amount of millet in their diet (Murphy et al., 2013; Wilkin et al., 2020b). This links them to agropastoralist cultures of the Western Steppe, where the intensification of millet cultivation occurred during the second millennium BCE (Ventresca Miller and Makarewicz, 2019). We analyzed individuals from one Sagly/Uyuk site in this study: Chandman Mountain (CHN).

*Pazyryk (500-200 BCE).* This culture is known mainly for its type site of Pazyryk, whose large tombs contain numerous exotic imports, including silks from China and textiles from Achaemenid Persia (Rudenko, 1970). Pazryk burials are found mostly within the northern Altai areas of Russia, far eastern Kazakhstan (Samashev, 2011) and northwestern Mongolia (Törbat et al., 2009). Similar to Sagly/Uyuk and other ‘Saka’ style graves, Pazyryk burials are marked by round piles of stones. Beneath these stone piles, however, most Pazyryk graves have smaller wooden chambers with only one or two persons; their size and burial goods vary greatly, though many of them are accompanied by whole horses laid beside the burial chamber (Kubarev and Shul’ga, 2007) (https://edmond.mpdl.mpg.de/imeji/collection/2ZJSw35ZTTa18jEo). No new Pazyryk individuals were included in this study; however, they are important to consider because the northern Altai practice of whole horse burials later appears in scattered central Mongolia cemeteries of the subsequent Xiongnu period. Genome-wide data from Pazyryk individuals have been previously reported from site of Berel in Altai region of Kazakhstan (Unterländer et al., 2017).

Comparative genomic data are available for several contemporaneous sites in neighboring regions, including: (1) the early Sarmatian site Pokrovka in southwestern Russia, dating to ca. 500-100 BCE (Unterländer et al., 2017), a Scythian individual from the Samara region dating to ca. 300 BCE (Mathieson et al., 2015), and nine Sarmatian sites in southwestern Russia (Chebotarev V, Kamyshevahsky X, Nesvetay II, Nesvetay IV and Tengyz), northern Kazakhstan (Bestamak and Naurzum Necropolis), and the southern Ural region (Cherniy Yar and Temyaysovo) (Damgaard et al., 2018b; Krzewińska et al., 2018); (2) the Pazyryk site of Berel in the Altai, dating to ca. 400-200 BCE (Unterländer et al., 2017); (3) the Saka sites of Borli, Karasjok-1, Karasjok-6, Nazar-2, Sjartas (Zjartas), and Taldy-2 in Kazakhstan (Damgaard et al., 2018b), and the sites of Basquiat I, Keden, and Ornek in the Tian Shan (Damgaard et al., 2018b); and (4) the Tagar site of Grishkin Log 1 in the Minusinsk Basin (Damgaard et al., 2018b). Data from three other potentially relevant sites (the Aldy-Bel site Arzhan 2 in Tuva, dating to ca. 700-500 BCE, and the Zevakino-Chilikta sites Ismailovo and Zevakino in eastern Kazakhstan, dating to ca. 900-600 BCE; (Unterländer et al., 2017) were excluded from analysis due to insufficient genetic coverage for comparison.

##### Xiongnu (ca. 200 BCE to 100 CE)

During the late first millennium BCE, a radically new multi-regional political entity formed in Mongolia, known as the Xiongnu empire. The Xiongnu empire is attested not only by historical records but also by ample archaeological remains throughout Inner Asia (Brosseder and Miller, 2011; Honeychurch, 2015). For roughly three centuries the Xiongnu ruled from their core realms in central and eastern Mongolia, expanding into western Mongolia, northern China and eastern Baikal, as well as making inroads into more distant regions in Central Asia. Most graves of the Xiongnu period were shaft pits set beneath thick rings of stones on the surface. These burials represent the vast network of regional and local elites and not the “commoner” people of Xiongnu society, whose burials are far less conspicuous, lying under small piles of stones or in unmarked pits. The graves of the uppermost ruling elites of the empire, on the other hand, were constructed on a far grander scale than that of ring graves.

While ring grave structures are found throughout the entire Xiongnu era, prestige accoutrements (and to some degree burial rituals) changed during the course of the empire. According to these changes, we can discern a general division between Early (200-50 BCE) and Late (50 BCE - 100 CE) Xiongnu periods (Miller, 2014). Overall, Xiongnu graves are marked by a dramatic increase in grave goods and furnishings as compared to previous time periods and cultures in Mongolia. As the Xiongnu expanded their empire, they conquered numerous neighboring groups to their east and west as well as subduing their Han Dynasty neighbors to the south (Di Cosmo, 2002). They continually traded and warred with Han China, defying the Great Wall boundaries, and held significant sway over the Silk Road kingdoms of Central Asia (Hulsewé, 1979). The findings of exotic items from China, Persia and the Mediterranean attest to these far-flung interactions, with Egyptian-style faience beads in graves of local elites and Roman glass bowls in the tombs of the rulers (Miller and Brosseder, 2017; Eregzen, 2011). The end of the Xiongnu period ca. 100 CE is marked by the widespread decline of Xiongnu power and influence following defeats by the Xianbei in northeastern China and the Han Dynasty of China, although isolated groups from the Xiongnu empire continued to exist in northern China until the 5th century CE.

*Early Xiongnu (200-50 BCE).* Prestige items during the Early Xiongnu period are dominated by large bronze belt pieces; however, burial customs within graves of the Early period varied to a great degree between regions. One example of this occurs at Salkhityn Am cemetery, where rituals of ring graves show a high degree of variation, even including offerings of whole horses that are more typical of Altai elites such as those in Pazyryk graves (Ölziibayar et al., 2019) (https://edmond.mpdl.mpg.de/imeji/collection/2ZJSw35ZTTa18jEo). We analyzed individuals from three Early Xiongnu sites in this study: Astyn Gol (AST), Buural Uul (BAU/BRL/BUU), and Salkhityn Am (SKT).

*Late Xiongnu (50 BCE - 100 CE).* Prestige items in the Late Xiongnu period shift to more iron items, often covered with gold foil or even inlaid with precious stones, and increasingly focused on long-distance exotic materials. At the same time, burial customs in ring graves throughout the empire become more regularized. Most elites were buried in wooden coffins in shaft pits with livestock portions and ceramic vessels set beside the coffin (https://edmond.mpdl.mpg.de/imeji/collection/2ZJSw35ZTTa18jEo). During the Late period, the high ruling Xiongnu elites adopted a radically new form of burial structure. These square tombs were marked on the surface by rectangular stone structures with trapezoidal ‘ramp’ entryways, their burial pits were extremely deep, and wooden coffins were decorated and nested within larger wooden chambers (https://edmond.mpdl.mpg.de/imeji/collection/2ZJSw35ZTTa18jEo). We analyzed individuals from 26 Late Xiongnu sites in this study: Atsyn Am (ATS), Baruun Mukhdagiin Am (BAM), Baruun Khovdiin Am (BRU), Burkhan Tolgoi (BTO), Chandman Mountain (CHN), Delgerkhaan Uul (DEL), Khanan Uul (DOL), Duulga Uul (DUU); Emeel Tolgoi (EME), Khudgiin Am (HUD), Ikh Tokhoirol (IKT), Il’movaya Pad (IMA), Jargalantyn Am (also called Jargalantyn Khondii; JAA/JAG), Tarvagatain Am (also called Khoit Tsenkher; KHO), Naimaa Tolgoi (NAI), Sant Uul (SAN), Solbi Uul (SOL), Songino Khairkhan (SON), Takhityn Khotgor (TAK), Tavan Tolgoi (TAV), Tevsh Mountain (TEV), Ulaanzuukh (ULN), Ovgont (UVG), Yuroo II (YUR), Tamiryn Ulaan Khoshuu (also called Burkhan Tolgoi; BUR/TMI/TUH/TUK), and Uguumur Uul (UGU).

Comparative genomic data are available for a few contemporaneous sites in neighboring regions, including: (1) two early Xiongnu individuals from Khövsgöl (Hovsgol) province dating to 50-350 BCE (Damgaard et al., 2018b); (2) a late Xiongnu royal tomb (DA39.SG) in Arkhangai dating to 80-160 CE (Damgaard et al., 2018b).

##### Early Medieval (ca. 100-850 CE)

After the fall of the Xiongnu, Xianbei groups from northeast China pushed into Mongolia, although historical and archaeological evidence for the establishment of large and long-lasting Xianbei polities appears only in northern China, not in Mongolia (Miller, 2016). One individual in this study (TUK001) at the site of Tamiryn Ulaan Khoshuu (Burkhan Tolgoi) dates to the era of Xianbei power in Inner Asia; however, there is no cultural context that could affirm affiliation with the Xianbei or other groups of northeastern China. Instead, recent excavations at this site have yielded artifacts, such as pottery from the Kwarezm oasis cultures near the Aral Sea and coins of the Sassanian Persian empire, that indicate significant interactions with areas in Central Asia and much farther west. In the mid-fourth century, a large polity known as the Rouran purportedly took over all of Mongolia; however, there is little recorded history about the Rouran (Kradin, 2005), and only one grave found so far can be dated to the Rouran era (Li et al., 2018; Nei Menggu zizhiqu wenwu kaogu yanjiusuo and International Institute for the Study of Nomadic Civilizations, 2015). The archaeology of the second to sixth centuries in Mongolia, i.e., the Xianbei and Rouran eras, constitute an extremely new field of research (Odbaatar and Egiimaa, 2018).

The most prominent political entities in the Early Medieval era are the Türk and Uyghur empires, the latter being an immediate dynastic takeover from the former. Numerous burials of the Türk era have been unearthed in Mongolia. By contrast, far fewer Uyghur burials have been identified and excavated to date.

*Türk (550-750 CE).* Göktürkic tribes of the Altai Mountains established a political structure across Eurasia beginning in 552 CE, with an empire that ruled over Mongolia from 581-742 CE (Golden, 1992). A brief period of disunion occurred between 659-682 CE, during which the Chinese Tang dynasty laid claim over Mongolia. One individual from this study (TUM001) was a sacrificial person within the ramp of a Chinese-style tomb in central Mongolia dating (via tomb inscription) to this exact time period. The other Türkic era individuals in this study were excavated from conventional Türkic style graves. Features of the Türk period include numerous stone statues and stone offering boxes across the steppe landscape, while burials are often arranged as small groups of graves or single graves inserted into burial grounds of earlier Bronze to Iron ages. Most elites were interred within wooden coffins as single individuals buried beneath a stone mound, and many were buried with whole horses equipped with riding gear (https://edmond.mpdl.mpg.de/imeji/collection/2ZJSw35ZTTa18jEo). Other burials were in small wooden coffins without whole horses beside them. We analyzed individuals from 5 Türk sites in this study: Nomgonii Khundii (NOM), Shoroon Bumbagar (Türkic mausoleum; TUM), Zaan-Khoshuu (ZAA), Uliastai River Lower Terrace (ULI), and Umuumur uul (UGU).

*Uyghur (750-850 CE).* In the mid-eighth century, Uyghur tribes from the Upper Yenesei region overthrew the Türk rulers and immediately established a Mongolia-based empire, taking over the Orkhon valley as their capital and establishing a dynasty from 744-840 CE (Mackerras, 1972). Most Uyghur period burials excavated to date, including those from the Olon Dov burial ground (OLN) included in this study, lie in the vicinity of the Kharbalgas capital in the Orkhon Valley. Most of the burials excavated were discovered beneath large earthen enclosures that contained ritual structures for venerating the uppermost elites. These conspicuous ritual enclosures occur as single monuments or in small groups, and they are found in several locations throughout the foothills of the nearby the Uyghur capital. These monumental tombs with ramp entries and vaulted brick chambers were likely reserved for the ruling nobility of the Uyghur empire (Odbaatar, 2016) (https://edmond.mpdl.mpg.de/imeji/collection/2ZJSw35ZTTa18jEo). One individual in this study (OLN006) was found in a monumental tomb. A second, more modest category of Uyghur burials consists of stone structures placed on the surface, either square or round in shape, that contain multiple individuals (Erdenebat 2016) (https://edmond.mpdl.mpg.de/imeji/collection/2ZJSw35ZTTa18jEo). Dozens of these burials have been documented at Olondov (Erdenebat et al., 2012), and most of the Uyghur individuals in this study are from such graves. One such grave at Olon Dov, grave 19, contained the remains of multiple individuals, six of whom are included in this study. Other scattered examples of single Uyghur graves have been found in Mongolia, and we analyzed one of these (ZAA001) from the site of Zaan-Khoshuu. Although a few large ‘royal’ complexes have been found elsewhere in central Mongolia, no significant cemeteries outside the capital region have yet been found. We analyzed individuals from two Uyghur sites in this study: Olondov (OLN) and Zaan-Khoshuu (ZAA).

Comparative genomic data are available for contemporaneous sites in neighboring regions, including: (1) Alan sites in North Ossetia-Alania and Alan 51 from the Caucasus (Damgaard et al., 2018b); (2) the Rouran site of Khermen Tal site from Arkhanggai, Mongolia (Li et al., 2018).

##### Late Medieval (ca. 850-1650 CE)

This period in Mongolia is dominated mostly by the power struggles of two empires established by the Khitans (907-1125 CE) and the Mongols (1206-1368 CE). Burials from the Khitan era are virtually unknown in Mongolia, whereas numerous graves from the Mongol era have been documented and unearthed. So-called cave burials are known from both periods (Bemmann and Nomguunsüren, 2012), but their human remains were not included in this study.

*Khitan (ca. 900-1100 CE).* After the collapse of the Uyghur empire in Mongolia in 840 CE, the Khitans of northeast China established the powerful Liao Dynasty in 916 CE. Although based in Manchuria, the Khitans conquered and controlled the steppe of present-day Mongolia through a system of garrisons and long walls, deporting people from other conquered regions, such as northern Korea, to Mongolia (Kradin and Ivliev, 2008). The dissolution of the Khitan empire in 1125 CE led to a power vacuum in Mongolia until the rise of Chinggis Khan in the early 13th century CE. To date, very few Khitan era graves have been found in Mongolia. The site of Ulaan Kherem II (ULA) has yielded one Khitan-era grave (ULA001), and two Khitan-era unmarked graves of a man and woman were also discovered during the excavation of a Xiongnu settlement at Zaan Khoshuu (ZAA) beneath an older collapsed building (Nei Menggu zizhiqu wenwu kaogu yanjiusuo and International Institute for the Study of Nomadic Civilizations, 2015; Ochir et al., 2016). The man, found in a pit within the pit-house, was buried in a simple pit with a quiver and arrows. The woman, found nearby a pit-house, was buried in full dress and placed in a supine position with her head to the northwest inside a wooden coffin, along with pottery of the Khitan era (https://edmond.mpdl.mpg.de/imeji/collection/2ZJSw35ZTTa18jEo). These burials are significantly different in form and structure from other Khitan burials in northern China, where the core of the empire was located. At present, no monumental tombs of high Khitan elites have been found in Mongolia.

*Mongol (ca. 1200-1400 CE).* The home base of the Mongol tribe was in the forest-steppe zone at the Onon and Kerülen (Kherlen) rivers in northeastern Mongolia. From this core region they successfully conquered the Eurasian steppes and most of their sedentary neighbors in the adjacent regions. Historical records indicate that they transferred a large number of defeated people, war captives and slaves all over their growing empire; they also fostered trade, the exchange of knowledge, techniques, and technicians (Allsen, 2015). Mongol burials are typically situated in small groups on flat southern slopes or placed within Bronze and Iron Age cemeteries. They are marked above ground with stones in an irregular, flat, oval or rounded, one-layered setting (https://edmond.mpdl.mpg.de/imeji/collection/2ZJSw35ZTTa18jEo). The pit is normally between 50-150 cm deep, rarely deeper, and very seldom constructed as a niche. Typical Mongol burials contain one person placed in a supine position and sometimes in a wooden coffin, with the head to the north. A very characteristic feature of Mongol burials is the inclusion of a tibia from small livestock, mostly sheep, placed near the head and sometimes in a vessel. There are two ideal burial types concerning grave goods: one equipped with bow, arrow, quiver, horse equipment, and belt with attachments, and a second with scissors, a comb, a mirror, beads and a *bogtag* – a long hat made out of birch bark, covered with silk and decorated with golden ornament. Graves of these standard types are spread all over Mongolia, and at present no regional differences have been reported and no monumental burials are known (Erdenebat, 2009; Lkhagvasüren, 2007). The Mongol burials included in this study are of these types, which consist of the burials of local steppe warriors and elites of the Mongol empire. Individuals from the cosmopolitan capital of Karakorum were not sampled in this study.

Historical records mention a large amount of foreign people who migrated, whether for opportunity or by force, into the core steppe regions of the Mongol empire (Allsen, 2015). Given the intriguing results of extreme genetic diversity among local elite constituents for the Xiongnu era, one might expect a similar or even greater diversity during the Mongol era. However, within the core steppe realms, lower local levels of the Mongol empire appear not to have been as open. The supposed mass of incoming foreigners must be sought in other burial contexts, not those of Mongol tradition.

*Unclassified.* In addition to these main types, we also analyzed individuals from three Late Medieval sites containing burials with uncertain or unclassified cultural affiliations: Shunkhlai Mountain (SHU), Tsaidam Bag (TSB/TSI), Uushigiin Uver (UUS).

Because no comparative genomic data are available for contemporaneous sites, we compared our Late Medieval data to modern Mongolic speaking populations (Buryat, Khamnegan, Kalmyk, Mongol, Daur, Tu, Mongola) (Jeong et al., 2019; Lazaridis et al., 2014; Patterson et al., 2012).

### Method Details

#### Radiocarbon dating of sample materials

A total of 30 new radiocarbon dates were obtained by accelerator mass spectrometry (AMS) of bone and tooth material at the Curt-Engelhorn-Zentrum Archäometrie (CEZA) in Mannheim, Germany (n = 28) and the University of Cologne Centre for Accelerator Mass Spectrometry (CologneAMS) (n = 2). Selection for radiocarbon dating was made for all burials with ambiguous or unusual burial context and for all individuals appearing as genetic outliers for their assigned period. Uncalibrated direct carbon dates were successfully obtained for all bone and tooth samples (Table S4). An additional 74 previously published radiocarbon dates for individuals in this study were also compiled and analyzed, making the total number of directly dated individuals in this study to 98 (104 total dates). Dates were calibrated using OxCal v.4.3.2 (Ramsey, 2017) with the r:5 IntCal13 atmospheric curve (Reimer et al., 2013).

Of the 104 total radiocarbon dates analyzed in this study, 25 conflicted with archaeological period designations reported in excavation field notes or previous publications (Table S4). Four burials of uncertain cultural context were successfully assigned to the Middle/Late Bronze Age (BIL001, MIT001) and Late Medieval periods (UUS002, ZAA003). One burial originally assigned to the Late Medieval period was reassigned to the pre-Bronze Age following radiocarbon dating (ERM001), and one burial originally assigned to the Middle/Late Bronze Age was similarly reassigned to the Early Bronze Age (IAG001). This suggests that early burials may be underreported in the literature because they are mistaken for later graves. Likewise three burials originally classified as Late Medieval were found to be hundreds or thousands of years older, dating to the Early Medieval (TSB001) and Middle/Late Bronze Age (ULZ001, TSI001) periods. Although some highly differentiated burial forms can be characteristic of specific locations and time periods, simple burial mounds also exist for all periods and - lacking distinctive features - they can be difficult if not impossible to date without radiometric assistance.

In addition to early burials being mistaken for later ones, late burials were also misassigned to earlier periods. For example, three burials originally assigned to the Middle/Late Bronze Age were determined to date to the Early Iron Age (DAR001), Xiongnu (ULN004), and Late Medieval (SHU001) periods, and two Early Iron Age (CHN010, CHN014), six Xiongnu (TUK001, UGU001, DUU002, BRL001, BAU001, DEE001), and two Early Medieval (ULA001, ZAA005) graves were likewise reassigned to later periods following radiocarbon dating. Part of the difficulty in correctly assigning archaeological period to later burials relates to the frequent reuse of earlier graves and cemeteries by populations from later periods. The site reports of several Xiongnu excavations noted burial intrusions, displaced burials, and other indications of burial disturbance and reuse. However, evidence of burial reuse may also be subtle and easily overlooked. As such, we recommend great care in making cultural or temporal assignments at multi-period cemeteries or for any burials showing evidence of disturbance.

#### Sampling for ancient DNA recovery and sequencing

Sampling was performed on a total of 169 teeth and 75 petrosal bones from fragmented crania originating from 225 individuals (Table S1C). For 14 individuals, both a tooth and a petrosal bone were sampled (Table S2B). For three individuals, two teeth were sampled, and for one individual, two teeth and one petrosal bone were sampled (Table S2B). For Mongolian material, whole teeth and petrosal bone (except ERM) were collected at the physical anthropology collections of the National University of Mongolia and the Institute for Archaeology and Ethnology under the guidance and supervision of M. Erdene and S. Ulziibayar. Petrosal and tooth material from ERM were provided by J. Bemmann. For Russian material, whole teeth alongside petrosal bone or bone were collected from the Institute for Mongolian, Buddhist, and Tibetan Research as well as the Buryat Scientific Center, Russian Academy of Sciences (RAS). After collection, the selected human skeletal material was transferred to the Max Planck Institute for the Science of Human History (MPI-SHH) for genetic analysis.

#### Laboratory procedures for genetic data generation

Genomic DNA extraction and Illumina double-stranded DNA (dsDNA) sequencing library preparation were performed for all samples in a dedicated ancient DNA clean room facility at the MPI-SHH, following published protocols (Dabney et al., 2013) with slight modifications (Mann et al., 2018). We applied a partial treatment of the Uracil-DNA-glycosylase (UDG) enzyme to confine DNA damage to the ends of ancient DNA molecules (Rohland et al., 2015). Such “UDG-half” libraries allow us to minimize errors in the aligned genetic sequence data while also maintaining terminal DNA misincorporation patterns needed for DNA damage-based authentication. Library preparation included double indexing by adding unique 8-mer index sequences at both P5 and P7 Illumina adapters. After shallow shotgun sequencing for screening, we enriched libraries of 195 individuals with ≥ 0.1% reads mapped on the human reference genome (hs37d5; GRCh37 with decoy sequences) for approximately 1.24 million informative nuclear SNPs (“1240K”) by performing an in-solution capture using oligonucleotide probes matching for the target sites (Mathieson et al., 2015). In addition, eight samples (see Tables S2A and S2B) were also selected and built into single-stranded DNA (ssDNA) sequencing libraries for comparison. Single-end 75 base pair (bp) or paired-end 50 bp sequences were generated for all shotgun and captured libraries on the Illumina HiSeq 4000 platform following manufacturer protocols. Output reads were demultiplexed by allowing one mismatch in each of the two 8-mer indices.

### Quantification and Statistical Analysis

#### Sequence data processing

Short read sequencing data were processed by an automated workflow using the EAGER v1.92.55 program (Peltzer et al., 2016). Specifically, in EAGER, Illumina adaptor sequences were trimmed from sequencing data and overlapping sequence pairs were merged using AdapterRemoval v2.2.0 (Schubert et al., 2016). Adaptor-trimmed and merged reads with 30 or more bases were then aligned to the human reference genome with decoy sequences (hs37d5) using BWA aln/samse v0.7.12 (Li and Durbin, 2009). A non-default parameter “-n 0.01” was applied. PCR duplicates were removed using dedup v0.12.2 (Peltzer et al., 2016). Based on the patterns of DNA misincorporation, we masked the first and last two bases of each read for UDG-half libraries and 10 bases for non-UDG single-stranded libraries, using the trimbam function in bamUtils v1.0.13 (Jun et al., 2015), to remove deamination-based 5′ C>T and 3′ G>A misincorporations. Then, we generated pileup data using samtools mpileup module (Li and Durbin, 2009), using bases with Phred-scale quality score ≥ 30 (“-Q30”) on reads with Phred-scale mapping quality score ≥ 30 (“-q30”) from the original and the end-masked BAM files. Finally, we randomly chose one base from pileup for SNPs in the 1240K capture panel for downstream population genetic analysis using the pileupCaller program v1.2.2 (https://github.com/stschiff/sequenceTools). For C/T and G/A SNPs, we used end-masked BAM files, and for the others we used the original unmasked BAM files. For the eight ssDNA libraries, we used end-masked BAM files for C/T SNPs, and the original BAM files for the others.

In cases where more than one sample was genetically analyzed per individual, we compared the amount of human DNA between samples. For pairs of petrous bone and teeth, human DNA was higher in the petrous bone in 8 of 13 individuals, and higher in the teeth of 5 of 13 individuals (Table S2B). In addition, intra-individual sample variation was high, as evidenced by the high variance observed between paired tooth samples (Table S2B). Finally, in a comparison of dsDNA and ssDNA libraries, ssDNA libraries yielded a higher endogenous content in 7 of 8 library pairs. All data from paired samples were merged prior to further analysis.

Of the 225 new individuals analyzed, 18 failed to yield sufficient human DNA (< 0.1%) on shotgun screening (Table S2A) and a further 6 individuals failed to yield at least 10,000 SNPs after DNA capture (Table S2A). These 24 individuals were excluded from downstream population genetic analysis.

#### Data quality authentication

To confirm that our sequence data consist of endogenous genomic DNA from ancient individuals with minimal contamination, we collected multiple data quality statistics. First, we tabulated 5′ C>T and 3′ G>A misincorporation rate (Table S2A) as a function of position on the read using mapDamage v2.0.6 (Jónsson et al., 2013). Such misincorporation patterns, enriched at the ends due to cytosine deamination in degraded DNA, are considered as a signature of the presence of ancient DNA in large quantities (Sawyer et al., 2012). Second, we estimated mitochondrial DNA contamination for all individuals using the Schmutzi program (Renaud et al., 2015). Specifically, we mapped adaptor-removed reads to the revised Cambridge Reference Sequence of the human mitochondrial genome (rCRS; NC_012920.1), with an extension of 500 bp at the end to preserve reads passing through the origin. We then wrapped the alignment to the circular reference genome using circularmapper v1.1 (Peltzer et al., 2016). The contDeam and schmutzi modules of the Schmutzi program were successively run with the world-wide allele frequency database from 197 individuals, resulting in estimated mitochondrial DNA contamination rates for each individual (Table S2A). Last, for males, we also estimated the nuclear contamination rate (Table S2A) based on X chromosome data using the contamination module in ANGSD v0.910 (Korneliussen et al., 2014). For this analysis, an increased mismatch rate in known SNPs compared to that in the flanking bases is interpreted as the evidence of contamination because males only have a single copy of the X chromosome and thus their X chromosome sequence should not contain polymorphisms. We report the Method of Moments estimates using the “method 1 and new likelihood estimate,” but all the other estimates provide qualitatively similar results.

Ten individuals were estimated to have > 5% DNA contamination (mitochondrial or X) or uncertain genetic sex (Table S2A); these individuals were excluded from downstream population genetic analysis.

#### Genetic sex typing

We calculated the genetic sequence coverage on the autosomes and on each sex chromosome in order to obtain the ratio between the sex chromosome coverage and the autosome coverage. For 1240K capture data, we observe females to have an approximately even ratio of X to autosomal coverage (X-ratio of ∼0.8) and a Y-ratio of 0, and males to have approximately half the coverage on X and Y as autosomes (∼0.4). Genetic sex could be determined for a total of 224 individuals, of which 100 were female and 124 were male (Table S2C).

#### Uniparental haplogroup assignment

We called mitochondrial consensus sequence from the Schmutzi output using the log2fasta program in the Schmutzi package, with quality threshold of 10. We then assigned each consensus sequence into a haplogroup (Table S2C) using the HaploGrep 2 v2.1.19 (Weissensteiner et al., 2016). For the Y haplogroup assignment, we took 13,508 Y chromosome SNPs listed in the ISOGG database and made a majority haploid genotype call for each male using pileupCaller (with “-m MajorityCalling” option). We assigned each individual into a haplogroup (Table S2C) using a patched version of the yHaplo program (Poznik, 2016) downloaded from https://github.com/alexhbnr/yhaplo. This version takes into account high missing rate of aDNA data to prevent the program from stopping its root-to-tip haplogroup search prematurely at an internal branch due to missing SNP and therefore assigning a wrong haplogroup. We used “–ancStopThresh 10” following the developer’s recommendation. Haplogroup assignments are shown in Figures S2A and S2B.

#### Estimation of genetic relatedness

To evaluate the relatedness within our dataset, we calculated pairwise mismatch rate of haploid genotypes on automosomes across all individuals. The pairwise mismatch rate for each pair of individuals, is defined as the number of sites where two individuals have different alleles sampled divided by the total number of sites that both individuals have data. The pairwise mismatch rate between unrelated individuals is set as the baseline and the coefficient of relationship is inversely linear to the baseline pairwise mismatch rate. More detailed description can be found in the Supplemental Materials of (Jeong et al., 2018).

A total of 15 first or second degree genetic relationships were observed across the dataset (Table S2D), of which 10 date to the Xiongnu era. Additionally, in one case, a tooth and petrosal bone thought to belong to one individual (AT-871) were later discovered to belong to two different individuals (OLN001.A and OLN001.B). In another case, two teeth (AT-728 and AT-729) thought to belong to different individuals were found to originate from the same individual (TUK001/TAV008).

#### Data filtering and compilation for population genetic analysis

To analyze our dataset in the context of known ancient and modern genetic diversity, we merged it with previous published modern genomic data from i) 225 worldwide populations genotyped on the Human Origins array (Jeong et al., 2019; Lazaridis et al., 2014), ii) 300 high-coverage genomes in the Simons Genome Diversity Project (“SGDP”) (Mallick et al., 2016), and iii) currently available ancient genomic data across Eurasian continent (Allentoft et al., 2015; Damgaard et al., 2018a; 2018b; Fu et al., 2014; 2016; Haak et al., 2015; Haber et al., 2017; Harney et al., 2018; Jeong et al., 2016; 2018; Jones et al., 2015; Kılınç et al., 2016; Lazaridis et al., 2016, 2017; Mathieson et al., 2015; 2018; McColl et al., 2018; Narasimhan et al., 2019; Raghavan et al., 2014; 2015; Rasmussen et al., 2010; 2014; 2015; Sikora et al., 2019; Unterländer et al., 2017; Yang et al., 2017). We obtained 1,233,013 SNP sites (1,150,639 of which on autosomes) across our dataset when intersecting with the SGDP dataset, and 597,573 sites (593,124 of which on autosomes) when intersecting with the Human Origins array.

#### Analysis of population structure and relationships

We performed principal component analysis (PCA) on the merged dataset with the Human Origins data using the smartpca v16000 in the Eigensoft v7.2.1 package (Patterson et al., 2006). Modern individuals were used for calculating PCs (Figure S3A), and ancient individuals were projected onto the pre-calculated components using “lsqproject: YES” option (Figure 2; Figure S3B). To characterize population structure further, we also calculated *f3* and *f4* statistics using qp3Pop v435 and qpDstat v755 in the admixtools v5.1 package (Patterson et al., 2012). We added “*f4mode: YES*” option to the parameter file for calculating *f4* statistics.

#### Admixture modeling using qpAdm

For modeling admixture and estimating ancestry proportions, we applied qpWave v410 and qpAdm v810 in the the admixtools v5.1 package (Patterson et al., 2012) on the merged dataset with the SGDP data to maximize resolution. To model the target as a mixture of the other source populations, qpAdm utilizes the linearity of *f4* statistics, i.e., one can find a linear combination of the sources that is symmetrically related to the target in terms of their relationship to all outgroups in the analysis. qpAdm optimizes the admixture coefficients to match the observed f4 statistics matrix, and reports a *p-value* for the null hypothesis that the target derives their ancestry from the chosen sources that are differently related to the outgroups (i.e., when p < 0.05, the null hypothesis is rejected so that the target is different from the admixture of chosen sources given the current set of outgroups). The chosen outgroups in qpAdm needs to be differentially related to the sources such that a certain major ancestry is “anchored” in the test, which is rather heuristic. We used qpWave to test the resolution of a set of outgroups for distinguishing major ancestries among Eurasians, as well as the genetic cladility between populations given a set of outgroups. We used a set of eight outgroup populations in our study: Central African hunter-gatherers Mbuti.DG (n = 5), indigenous Andamanese islanders Onge.DG (n = 2), Taiwanese Aborigines Ami.DG (n = 2), Native Americans Mixe.DG (n = 3), early Holocene Levantine hunter-gatherers Natufian (n = 6) (Lazaridis et al., 2016), early Neolithic Iranians Iran_N (n = 8) (Lazaridis et al., 2016; Narasimhan et al., 2019), early Neolithic farmers from western Anatolia Anatolia_N (n = 23) (Mathieson et al., 2015), and a Pleistocene European hunter-gatherer from northern Italy Villabruna (n = 1) (Fu et al., 2016).

To evaluate potential sex bias (Figure S2C), we applied qpAdm to both the autosomes (default setting) and the X chromosome (adding “chrom:23” to the parameter file) for comparing the difference in the estimated ancestry proportions. For a certain ancestry, we calculated sex-bias Z score using the proportion difference between P_A_ and P_X_ divided by their standard errors ($Z\;=\;\left(P_{A}-P_{X}/\sqrt{\sigma_{A}^{2}+\;}\sigma_{X}^{2}\right)$, where $\sigma_{A}$ and $\sigma_{X}$ are the corresponding jackknife standard errors, as previously performed in (Mathieson et al., 2018). Therefore a positive Z score suggests autosomes harbor a certain ancestry more than X chromosomes do, indicating male-driven admixture. A negative Z score, in contrast, suggests female-driven admixture. The qpAdm estimates from both autosomes and the X chromosome are available in Table S5K.

#### Dating admixture events via DATES

We used DATES v753 (Narasimhan et al., 2019) to estimate the time of admixture events in ancient individuals (Figure S6B), and convert the estimated admixture date in generation into years assuming 29 years per generation (Patterson et al., 2012). We show the admixture dates in years before present (Figure S6A) by adding the age of each ancient population (i.e., mean value of the midpoint of the 95% confidence interval of available calibrated 14C dates in each population). The standard error of DATES estimates come from the weighted block jackknife, an option in DATES parameter file. In the parameter file for running DATES, we used “binsize: 0.001,” “maxdis: 1,” “runmode: 1,” “mincount: 1,” “lovalfit: 0.45” in every run, same to the example file at https://github.com/priyamoorjani/DATES/blob/master/example/par.dates.

#### Phenotypic SNP analyses

We examined 49 SNPs in 17 genes (Table S2E) known to be associated with phenotypic traits or with positive selection in Eurasia (Jeong et al., 2018). Given the low coverage of ancient DNA data, we focused on five of these genes and calculated the likelihood of allele frequency for SNPs in each ancient population based on the counts of reads covering on the SNP following a published strategy (Mathieson et al., 2015). In the allele frequency calculation, we classified all ancient individuals before Middle/Late Bronze Age into a single group, and kept three genetic groups during MLBA (Khövsgöl_LBA, Altai_MLBA, Ulaanzuukh), two genetic groups during Iron Age (Chandman_IA, SlabGrave), one group for Xiongnu, one group for Early Medieval and one group for Late Medieval. We calculated allele frequency at five loci (Table S2E) that are associated with lactase persistence (LCT/*MCM6*), skin pigmentation (*OCA2*, *SLC24A5*), alcohol metabolism (*ADH1B*), and epithelial phenotypes including shovel-shaped incisor (*EDAR*) (Figure 5).

#### Genetic clustering of ancient individuals into analysis units

To further characterize the dynamic changes of the Eastern Steppe gene pools using group-based analyses, we quantitatively examined genetic differences among the analyzed individuals in combination with their temporal, archeological, and geographic information. We first obtained an approximate map of population structure by observing the position of ancient individuals on the PCA calculated from 2,077 present-day Eurasian individuals. PC1 separates geographically eastern and the western populations, PC2 captures the internal variations in eastern Eurasians, and PC3 captures variations in western Eurasians, thus allowing us to characterize an overall pattern of genetic changes through time and helping us to formulate explicit hypotheses regarding the genetic relationships between groups and individuals. Second, we computed outgroup-*f3* and symmetric-*f4* statistics to (1) quantify genetic similarity between individuals/groups falling together on PCA and (2) explore populations whose ancestry through admixture may have contributed to the differences observed between pairs of groups. Third, we identified representative ancient populations to serve as proxies for five distinct ancestries that we then further investigate (Table S3B). We changed the specific ancestry proxy for our test groups based on the temporal and archeological records accordingly. Using these ancestry proxies, we performed a formal admixture modeling using qpWave/qpAdm, which tests the difference between the target and a combination of the proxies (i.e., an admixture model) with regard to their genetic affinity to outgroups. We applied the same admixture models for test groups belonging to the same time/culture/geography category to compare them in a straightforward manner (Figures 3 and 4). In the following paragraphs, we describe each of the genetics-based analysis groups reported in our dataset, as well as the principles we applied to model their genetic ancestry using qpAdm.

##### Pre-Bronze Age

⋅ New genetic groups: eastMongolia_preBA(1), centralMongolia_preBA(1), and Fofonovo_EN(4)

⋅ Published genetic groups: DevilsCave_N(6), and Baikal_EN(9)

Our dataset adds three Ancient Northeast Asian (ANA)-related genetic groups before the start of the Bronze Age in eastern Eurasia. During this period, we observe the wide distribution of this ANA ancestry from Lake Baikal to the Russian Far East, spanning more than 2,000 km. As Baikal_EN has been modeled to have ∼10% Ancient North Eurasian (ANE) ancestry, we also investigated the possible genetic contribution from ANE in our pre-Bronze Age Mongolian and Baikal groups using Botai, AG3, MA1 and West_Siberia_N separately as ancestry proxies. We find ANE-related ancestry appears in centralMongolia_preBA and Fofonovo_EN only to a minor extent; ANE ancestry is not present in eastMongolia_preBA, which is instead characterized by only ANA-related ancestry (Table S5A).

##### Early Bronze Age

⋅ New genetic groups: Afanaseivo_Mongolia(2), Chemurchek_southAltai(2), Chemurchek_northAltai(2)

⋅ Published genetic groups: Afanaseivo(23), Okunevo_EMBA(19), and Baikal_EBA(5)

Our dataset adds three main genetic groups during the Early Bronze Age: Afanasievo_Mongolia, Chemurchek_southAltai and Chemurchek_northAltai. We group two individuals from Shatar Chuluu site (SHT001, SHT002) into Afanasievo_Mongolia as both are archaeologically classified into the Afanasievo cultural context and genetically indistinguishable from Afanasievo individuals from the Russian Altai-Sayan region (Figure S5A, S5C; Table S5B).

We group two individuals from Yagshiin Huduu site (IAG001, YAG001) into Chemurchek_southAltai as both are archaeologically classified to the Chemurchek cultural context and cluster together on PCA, providing the first genomic investigation of the Chemurchek culture. We observed that Chemurchek_southAltai has the highest genetic affinity to ANE-related groups (e.g., Botai) and secondary affinity to Iranian-related groups (Figures S5A and S5C). We tested Afanasievo as a potential ancestral source given the geographic overlap and similar burial posture between the Afanasievo and Chemurchek cultures (Taylor et al., 2019), however the 2-way model with Afanasievo as one of the two sources fails (Table S5B). The model also fails when using Okunovo (a neighboring group contemporaneous with Chemurchek that succeeds the Afanasievo culture) either as Afanasievo + Okunevo or Okunevo + Iranian (Table S5B). To further investigate the Iranian-related ancestry among the Chemurchek_southAltai, we tested four published groups from the BMAC genetic cluster (Gonur1_BA, Bustan_BA, Dzharkutan1_BA, and Sappali_Tepe_BA), four Chalcolithic/Bronze Age Iranian groups (Hajji_Firuz_C, Tepe_Hissar_C, Seh_Gabi_C, and Shahr_I_Sokhta_BA1), Eneolithic Turkmenistan and Tajikstan groups (Paikhai_EN, Sarazm_EN, and Tepe_Anau_EN) and Mesolithic Caucasus Hunter-Gatherer (CHG). Interestingly, 2-way models consisting of Botai + BMAC genetic cluster groups adequately model Chemurchek_southAltai with ∼40% ancestry proportion from the latter, while preceding Eneolithic Turkmenistan/Tajikistan groups do not (Table S5B). Spatiotemporally more distant groups, such as Chalcolithic Iranian groups or CHG, also adequately model Chemuchek_southAltai with similar ancestry proportions (Table S5B). The 3-way model consisting of Botai + BMAC + Afanasievo returns a positive contribution from Afanasievo but it is not significantly different from zero (11 ± 7%; Table S5B). Thus, despite some cultural similarities between steppe groups (Afanasievo, Okunevo) and Chemurchek, we observe a negligible level of genetic influence from the steppe populations among the Chemurchek_southAltai individuals analyzed here.

We find that Chemurchek_southAltai has a close genetic affinity to Dali_EBA (Figure S5A), an individual dating to ca. 2650 BCE with poor burial context from southeastern Kazakhstan who has admixed ANE-Iranian ancestry (see Narasimhan et al., 2019). Applying the same 2-way admixture models using Dali_EBA for comparison, we found that Dali_EBA also requires an additional Iranian-related ancestry but in a smaller proportion and that the models with Afanasievo as a source do not fit, replicating the findings in Chemurchek_southAltai.

The Chemurchek_northAltai genetic cluster, consisting of two female Chemurchek individuals (KUM001, KUR001) from the northern Altai, shows high genetic affinity to ANA groups, with a small proportion of ancestry consistent with Chemurchek_southAltai (Figures S5A, S5C; Table S5B). Like other Chemurchek or Chemurchek-like burials in the northern Altai, their mortuary architecture lacks some features that are classically associated with burials further south.

##### Middle and Late Bronze Age

⋅ New genetic groups: Altai_MLBA(7), Ulaanzuukh_SlabGrave(11/16), UAA001(1), KHI001(1), UUS001(1), KHU001(1) and TSI001(1)

⋅ Published genetic groups: Khövsgöl_LBA(17), ARS017(1), ARS026(1), Sintashta_MLBA(37), Krasnoyarsk_MLBA(18)

Our dataset adds two main genetic groups in the Eastern Steppe during the MLBA - Altai_MLBA and Ulaanzuukh_SlabGrave - to the previously published Khövsgöl_LBA from northern Mongolia (Jeong et al., 2018). Our new data substantially expand the geographic scope of genetically characterized MLBA populations in Mongolia, and reveal an overall picture of the population structure of the MLBA Eastern Steppe. The Altai_MLBA group contains seven individuals from the Altai-Sayan region (BER002, BIL001, ULZ001, ARS026, SBG001, ULI001, ULI003), who are admixed between the Western Steppe gene pool associated with Srubnaya/Sintashta/Andronovo cultures (“steppe_MLBA”) and the one associated with Khövsgöl_LBA/Baikal_EBA. Although the ancestry proportion estimates within this group vary along a cline, the Altai_MLBA represents the formation of a gene pool incorporating a substantial genetic influx from Western Steppe herders. Thus we classified them into one genetic group despite their archaeological and cultural differences (DSKC and unclassified burial types). This also explains the genetic profile of one outlier from Khövsgöl_LBA (ARS026), who now genetically falls within the Altai_MLBA group. Of note, one member of this group, ULZ001, is found not in the Altai, but in far eastern Mongolia.

The other genetic cluster, Ulaanzuukh_SlabGrave, contains 11 individuals with Ulaanzuukh burial type (BUL001, BUL002, ULN001, ULN002, ULN003, ULN005, ULN006, ULN007, ULN009, ULN010, ULN015) and 5 individuals with Slab Grave burials (see below), from eastern Mongolia. They all are classified into one single genetic group given their strong genetic homogeneity with ANA (Table S5C) and the geographic links between the two. This clustering of Ulaanzuukh and Slab Grave confirms previous archaeological hypotheses that the Slab Grave culture likely emerged out of the Ulaanzuukh gene pool. This genetic cluster also explains another Khövsgöl_LBA outlier, ARS017, who now genetically falls within the Ulaanzuukh_SlabGrave group, as well as a single individual with unknown burial type from central Mongolia, TSI001, who also falls into this cluster. Of note, one male Mönkhkhairkhan individual (KHU001) also has a large proportion of ancestry from Ulaanzuukh_SlabGrave in addition to his main genetic component from Baikal_EBA (Table S5C). Together, the individuals ARS017, TSI001, and KHU001 suggest contact with the Ulaanzuukh_SlabGrave group in northern, central Mongolia, even though these individuals were buried according to local burial customs. Overall, this Ulaanzuukh_SlabGrave genetic cluster is a continuation of the ANA easternMongolia_preBA gene pool (represented by SOU001) of 3,000 years earlier.

We also identified three outliers which do not fall into any of the three genetic clusters described above. UAA001 (Mönkhkhairkhan) from the Altai is well-fitted with 3-way admixture model using Afanasievo, Baikal_EBA and Gonur1_BA (Table S5C), despite the fact that they date to ∼1500 years after the Afanasievo culture. KHI001 (unclassified culture) from the Altai, is well-fitted with 3-way admixture model using Sintatash, Baikal_EBA and Gonur1_BA (*p-value* = 0.056; Table S5C), presenting minor genetic component from Gonur1_BA. Alternatively, KHI001 can also be modeled as a 2-way admixture between Afanasievo and Khövsgöl_LBA (*p-value* = 0.117; Table S5C); however, this model has a lower priority than the former model. UUS001 (DSKC) from Khövsgöl province is well-fitted with 3-way model using Sintashta, eastMongolia_preBA and Gonur1_BA (Table S5C). Given the temporal discordance between UUS001 and the eastMongolia_preBA individual (∼3,000 years), it is more likely that the admixing partner for UUS001 was related to the Ulaanzuukh cluster; Ulaanzuukh shares a high degree of ancestry with eastMongolia_preBA and is contemporaneous with the UUS001 individual, and some Ulaanzuukh individuals plot very close to the eastMongolia_preBA individual - SOU001 in PCA.

##### Early Iron Age

⋅ New genetic groups: Chandman_IA(9), Ulaanzuukh_SlabGrave(5/16),

⋅ Published genetic groups: Tagar(8), CentralSaka(6), TianShanSaka(10), Kazakhstan_Berel_IA(2; Pazyryk culture)

Our dataset adds two main genetic groups during EIA: one represented by Ulaanzuukh_SlabGrave and the other represented by the site of Chandman Mountain associated with the Sagly/Uyuk culture (Chandman_IA). In addition to the 11 Ulaanzuukh burials described above, four Slab Grave individuals (BOR001, DAR001, MIT001, SHU001) from eastern Mongolia also presented a homogeneous genetic profile with Ulaanzuuk and thus were merged into the Ulaanzuukh_SlabGrave analysis group (Table S5C). Interestingly, PTO001, a Trans-Baikal individual who is also archaeologically classified as Slab Grave, has a genetic profile that matches other Slab Grave individuals from eastern Mongolia, and we also merged PTO001 into the Ulaanzuukh_SlabGrave genetic cluster. The genetic profile of PTO001 is consistent with an archaeologically described expansion of the Slab Grave culture into the Baikal region during EIA (Losey et al., 2017).

The contemporaneous Chandman_IA from the Altai-Sayan region in western Mongolia has a genetic profile that matches the preceding Altai_MLBA cline. Since all individuals are from a single site and cluster together on PCA, we group them into a single analysis unit (“Chandman_IA”). Here, we use the Andronovo-associated dataset Krasnoyarsk_MLBA as the representative central steppe_MLBA group for admixture modeling because it is geographically closest to our test EIA groups. We first tested a 2-way admixture model of Krasnoyarsk_MLBA + Baikal_EBA, but it failed to adequately model the Chandmand_IA cluster, as did Karsnoyarsk_MLBA + Khövsgöl_LBA. Further changing the steppe_MLBA source from Karsnoyarsk_MLBA to Sintashta_MLBA did not rescue the 2-way admixture model. We then attempted a 3-way admixture model by adding Iranian-related ancestry as the third source, using a BMAC group from the Gonur Tepe site (Gonur1_BA) as a proxy. Using Krasnoyarsk_MLBA as the Steppe proxy, we observed 51.3% of Steppe, 42.2% of Baikal_EBA and 6.5% of Iranian ancestry in Chandman_IA (Table S5D).

Because it is *a priori* quite unlikely due to a long-distance migration from Bactria/Iran specific to Chandman_IA, we next applied the same 3-way models of Krasnoyarsk_MLBA/Sintashta_MLBA+Baikal_EBA+Gonur1_BA to four Iron Age central Asian groups (Tagar from Minusinsk Basin, Central Saka from central Kazakhstan, Kazakhstan_Berel_IA from eastern Kazakhstan, and Tian Shan Saka from Kyrgyzstan) and also to the Final Bronze Age group Karasuk. We observed that Iranian-related ancestry proportions range from ∼7%–28% in the tested Iron Age groups, while not required for Karasuk. In particular, the Tian Shan Saka, geographically closest to the Gonur Tepe site, has the highest amount of estimated Iranian-related ancestry. Because of cultural connections between the Sagly/Uyuk of Chandman_IA and the Saka generally (see section 2.4 above), it is possible that Saka and related groups in Tian Sian, Fergana and Transoxiana/Turan (such as the sampled Tian Shan Saka) are the proximal source of the Iranian ancestry in the Iron Age groups further to the north, such as Chandman_IA. To narrow down the spatiotemporal origin of this Iranian-related ancestry, we tested 3-way models using alternative Iranian-related groups as the proxy in the Tian Shan Saka: (1) three other post-BMAC groups (Bustan_BA, Dzharkutan1_BA, and Sappali_Tepe_BA) that fall into the BMAC genetic cluster with Gonur1_BA (Narasimhan et al., 2019), (2) Shahr_I_Sokhta_BA1 from the southeastern corner of Iran, (3) three Chalcolithic Iranian groups (Hajji_Firuz_C, Tepe_Hissar_C, Seh_Gabi_C), (4) two Iron Age groups from Pakistan (Katelai_IA, Loebanr_IA), (5) Eneolithic groups from Turkmenistan such as Geoksyur_EN, Parkhai_EN and Tepe_Anau_EN, and (6) Sarazm_EN fromwestern Tajikistan. All Iranian-ancestry proxies mentioned above except Hajji_Firuz_C and Seh_Gabi_C from the Zagros provide a well-fitted 3-way model (Table S5E). Therefore, for the Iron Age Eastern Steppe, genetic data alone can only narrow down the source of the Iranian ancestry to a broad region east of the Caspian Sea. Taken in context, though, we propose that this ancestry likely arrived via a local contact around the Transoxiana/Sogdiana region (i.e., the border between Kazakhstan, Uzbekistan and Kyrgyzstan).

For the prehistoric genetic groups described above, we used DATES to estimate the date of admixture between Western ancestry sources (WSH or the Iranian-related groups) and local ancestry sources (i.e., Khovsgol_LBA or Baikal_EBA) (Figure S6). As shown in Figure S6A, the estimated admixture date between Sintashta and Baikal_EBA for the Karasuk and Tagar is consistent with the admixture date observed in Altai_MLBA - at around 3,500 BP. For the Central Saka, Pazyryk (Kazakstan_Berel_IA) and Sagly/Uyuk (Chandman_IA), the admixture date is estimated to be a few centuries later, and the most recent admixture date is estimated for the Saka from Tian Shan. Notably, we find that the estimated admixture dates between Gonur1_BA and Baikal_EBA in the Iron Age groups are roughly consistent with the admixture dates for Sintashta (Figure S6A). However, because we are using a method designed for dating a 2-way admixture on what is best modeled as 3-way admixture in our study, we caution that these admixture dates should be interpreted with care.

##### Xiongnu Empire

⋅ New genetic groups: earlyXiongnu_west(6), earlyXiongnu_rest(6), SKT007(1), lateXiongnu(24), lateXiongnu_sarmatian(13), lateXiongu_han(8), TAK001(1), TUK002(1)

⋅ Published genetic groups: Xiongnu_WE(2), Xiongnu_royal(1, DA39.SG), Han_2000BP(2)

Our dataset reveals a great deal of previously uncharacterized genetic diversity during the Xiongnu period. For individual modeling, we tested every possible combination of five main ancestries: Steppe (Krasnoyarsk_MLBA, Sintashta, Srubnaya, Sarmatian, Chandman_IA), Gonur1_BA, Khövsgöl_LBA, Ulaanzuukh_SlabGrave, and Han. Considering the low resolution of individual modeling, we report selected working models that work for many individuals belonging to the same time period and archaeological context and that reflect qualitative trends observed in PCA. We observed that Iron Age Chandman_IA is a good Steppe ancestry proxy for many Xiongnu individuals, but there are also many who have western Eurasian ancestry in higher proportion than that of Chandman_IA. These individuals with high western Eurasian ancestry proportion show strong affinity to the Iranian-related ancestry that cannot be explained by the earlier Late Bronze Age steppe groups (e.g., Krasnoyarsk_MLBA, Sintashta_MLBA or Srubnaya). Instead, Gonur1_BA or Iron Age Sarmatian fit better with the genetic profile required. Also, a few individuals fall into the eastern Eurasian cline along PC2 and are explained as a combination of the eastern Eurasian gene pools, Ulaanzuukh_SlabGrave and present-day Han Chinese, without contribution from western Eurasian sources (Table S5F). We used high-coverage whole genome sequences of present-day Han Chinese (“Han.DG”; n = 4) as a proxy for the ancestry component that is currently broadly distributed across northern China and distinct from the component represented by Ulaanzuukh_SlabGrave further to the north. This is to achieve statistical power in our admixture modeling given that there are to date very few available ancient genomes that reflect this ancestry component. This is due to the fact that ancient China, Korea, Japan, and Southeast Asia remain mostly unsampled. We fully acknowledge the genetic diversity present within contemporary Han Chinese populations, and do not intend to claim by our admixture modeling a specific connection between the ancient populations within our study and present-day ethno-cultural identities.

For the group-based qpAdm modeling, we split Xiongnu into two categories based on their age - early Xiongnu and late Xiongnu. We further split early Xiongnu into two subgroups, earlyXiongnu_west (SKT010, SKT001, SKT003, SKT009, SKT008, AST001) and earlyXiongnu_rest (JAG001, SKT002, SKT004, SKT005, SKT006, SKT012), based on their individual modeling results, leaving out one individual outlier - SKT007 (Khövsgöl_LBA-like). The two previously published Xiongnu individuals grouped as “Xiongnu_WE” show a similar genetic profile to earlyXiongnu_rest, are dated to the early Xiongnu period, and are from the same valley as the two early Xiongnu sites (SKT and AST) in our dataset (Table S5F). For the late Xiongnu, we summarized their individual modeling results in Table S5G. Based on the individual modeling results, we set up three subgroups within late Xiongnu individuals to highlight key demographic processes and to use them for specific analyses such as sex-biased gene flow. First, we assigned 24 of 47 individuals into the main lateXiongnu group (BTO001, CHN010, DEL001, DOL001, IMA001-IMA008, JAA001, KHO006, KHO00, SAN001, SOL001, TEV002, TEV003, TUK003, UGU004, UGU011, ULN004, UVG001; Table S5F-G); this group is well modeled as a mixture of two main Iron Age clusters, Chandman_IA+Ulaanzuukh_SlabGrave (p = 0.316; 76.6 ± 0.8% from Ulaanzuukh_SlabGrave). Another 13 individuals have more western Eurasian ancestry than Chandman_IA and thus require a different western Eurasian source. Two of them (NAI002, BUR001) are explained by Chandman_IA+Gonur1_BA, a model for earlyXiongnu_west, but the remaining 11 need Sarmatian contribution, including three that are cladal to Sarmatian (BUR003, TM001, UGU010). Taken all 13 individuals as a group (lateXiongnu_sarmatian; BRL002, BUR001-BUR004, DUU001, HUD001, NAI001, NAI002, TMI001, UGU005, UGU006, UGU010), we infer a major contribution from a Sarmatian-related source into this group (75.7 ± 2.8%; Table S5F-G). On the other hand, we grouped eight individuals (ATS001, BAM001, BRU001, EME002, SON001, TUH001, TUH002, YUR001) into the third group lateXiongnu_han based on their affinity to Han Chinese and other East Asian populations that Ulaanzuukh_SlabGrave cannot explain (37.2 ± 10.6% from Han.DG; Table S5F-G). The previously published Xiongnu_royal individual shows substantial Han-related ancestry (Table S5F), similar to our lateXiongnu_han group. Further, the late Xiongnu individual YUR001 is an extreme East Asian outlier, who genetically resembles “Han_2000BP,” two Han empire soldiers recovered from a mass grave near a Han fortress in the southern Gobi (Damgaard et al., 2018b). These two groups, lateXiongnu_sarmatian and lateXiongnu_han, robustly support influxes of new ancestries both from the west and the east that were not previously observed in early Xiongnu or earlier populations. We left two individuals out of grouping, due to their unusual ancestry profiles: TAK001 mostly resembles Khövsgöl_LBA, and TUK002 is modeled as Chandman_IA+Ulaanzuukh_SlabGrave_Gonur1_BA (Table S5G). In contrast to the strong east-west genetic division among Bronze Age Eastern Steppe populations through the end of the Early Iron Age, the Xiongnu period is characterized by an extreme degree of genetic diversity and heterogeneity that does not have any obvious geographic correlation (Figure S7A).

##### Early Medieval

⋅ New genetic groups: TUK001(1), earlyMed_Türk(7), TUM001(1), earlyMed_Uyghur(12), OLN007(1)

Our dataset adds two main genetic groups during the early Medieval period in Mongolia: earlyMed_Türk and earlyMed_Uyghur. For each individual, we tested every possible combination of four main ancestries: Steppe (Sarmatian, Alan), Gonur1_BA, Ulaanzuukh_SlabGrave, and Han. The genetic contribution from Iranian-related ancestry becomes even more prominent in Türkic and Uyghur individuals, as seen from well-fitted models using the Alan, an Iranian pastoral population from the Caucasus (Table S5H). Overall, the Türkic and Uyghur individuals in this study show a high degree of genetic diversity, as seen in their wide scatter across PC1 in Figure 2. TUK001(250-383 CE), the earliest early Medieval individual in our dataset from a Xiongnu site with a post-Xiongnu occupation, has the highest western Eurasian affinity. This individual is distinct from Sarmatians, and likely to be admixed between Sarmatians and populations with BMAC/Iranian-related ancestry (Table S5H). Among the Türkic period individuals, TUM001 is a genetic outlier with mostly East Asian (Han_2000BP-like) ancestry. This individual was buried together with a knife and two dogs within the ramp of a Türkic era mausoleum. The mausoleum’s stone epitaph indicates that it was constructed for a diplomatic emissary of the Pugu tribe who was allegiant to the Chinese Tang Empire. His cremated remains were found within the tomb; TUM001 was likely this emissary’s servant (Ochir et al., 2013).

With respect to Uyghur burials, many consist of collective graves, and it has been suggested that such graves may contain the remains of kin groups (Erdenebat, 2016). We examined one such collective grave (grave 19) at the site of Olon Dov; however, of the six individuals analyzed in grave 19, there were no first degree relatives (parent-offspring pairs or sibling), and only two individuals (OLN002 and OLN003) exhibited a second degree (avuncular, grandparent-grandchild, or half-sibling) relationship. One Uyghur individual (OLN007) had markedly higher proportions of Han-related East Asian ancestry that cannot be explained by Ulaanzuukh_SlabGrave, and therefore grouped separately from the other earlyMed_Uyghur individuals (Table S5H).

##### Late Medieval

⋅ New genetic groups: lateMed_Khitan(3), lateMed_Mongol(61), SHU002(1)

Our dataset adds two main genetic groups during late Medieval in Mongolia: lateMed_Khitan and lateMed_Mongol. We used the same modeling strategy as used for the early Medieval period, and additionally explored the genetic cladality between every individual from the Mongol period and from modern Mongolic-speaking populations via qpWave (Figure S7B; Table S5J). Relatively few Khitan individuals (n = 3) were available for analysis, but all show high ANA-related ancestry (Table S5I). Mongol-era individuals (n = 61) are genetically more diverse and are cladal with modern Mongolic-speaking populations (Figure S7B). SHU002 is a single individual dated to the late Medieval period however without recognizable Mongol-like burial feature. Overall, Mongol period individuals characterized by a remarkable decrease in Western Eurasian ancestry compared to the preceding 1,600 years. They are best modeled as a mixture of ANA-like and East Asian-like ancestry sources, with only minor Western genetic ancestry. In addition, nearly a third of historic Mongol males (12/38) have Y haplogroup C2b, which is also widespread among modern Mongolians (Figure S3; Table S6); C2b is the presumed patrilineage of Genghis Khan (Zerjal et al., 2003).

## Supplemental Information

Table S1. Overview of Sites, Individuals, and Samples, Related to Figure 1

Table S2. Overview of Genetic Data, Related to Figure 5 and STAR Methods

Table S3. Genetic Groups and Reference Populations, Related to Figures 2, 3, and 4

Table S4. Direct AMS Radiocarbon Dates for Individuals in This Study, Related to Figure 1 and STAR Methods

Table S5. Population Modeling, Related to Figures 3, 4, and 5

Document S1. Article Translation in Mongolian: Евразийн Зүүн тал нутаг дахь 6000 жилийн динамик генетик түүх (Translation by Zoljargal Enkh-Amgalan, Enkhee Purev, Erdene Myagmar, Turbat Tsaagan, and Björn Reichhardt)
