# Supplementary material for: A Dynamic 6,000-Year Genetic History of Eurasia’s Eastern Steppe
Source: Cell. 2020 Nov 12;183(4):890–904.e29. doi: 10.1016/j.cell.2020.10.015 (PMC7664836; doi:10.1016/j.cell.2020.10.015)
Supplement: Document S1. Article Translation in Mongolian: Евразийн Зүүн тал нутаг дахь 6000 жилийн динамик генетик түүх (Translation by Zoljargal Enkh-Amgalan, Enkhee Purev, Erdene Myagmar, Turbat Tsaagan, and Björn Reichhardt) [file mmc6.pdf]

Евразийн Зүүн тал нутаг дахь 6000 жилийн динамик генетик түүх

Дотоод Ази, Монголын Эртний ДНХ

Зохиогчид

Чунвон Жүнг [1,2,†,*], Кэ Ванг[1,†], Шевин Вилкин[3], Виллиам Тимоти Трэал Тэйлор[3,4], Брайн К. Миллер[3,5], Ян Бемманн[6], Рафеалла Штал[1], Челсиа Чиовелли[1], Флориан Кнолле[1], Содномын Өлзийбаяр[7], Доржпүрэвийн Хатанбаатар[8], Диймаажавын Эрдэнэбаатар[9], Уламбаярын Эрдэнэбат[10], Аюудайн Очир[11], Ганболдын Анхсанаа[12], Чулуунхүүгийн Ванчигдаш[8], Баттулгын Очир[13], Чулуунбатын Мөнхбаяр[14], Дашзэвэгийн Түмэн[10], Алексей Ковалев[15], Николай Крадин[16,17], Билигто А. Базаров[17], Дэнис Миягашев[17], Прокопий Б. Коновалов[17], Елена Жамбалтарова[18], Алисиа Вентреска Миллер[3,19], Волфганг Хаак[1], Стефан Шиффэлс[1], Иоханнес Краус[1], Николь Бойвин[3], Мягмарын Эрдэнэ[10], Жессика Хэнди[1,20], Кристина Вариннер[1,21,*]

Зохиогчдын харьяалал:

[1]Герман улсын Енна хот дахь Макс-Планкийн Хүн Төрөлхтний Түүхийн Шинжлэх Ухааны Хүрээлэнгийн Археологийн тэнхим, 07745

[2]Бүгд Найрамдах Солонгос Улсын Сөүл хотын Сөүлийн Үндэсний Их Сургуулийн Биологийн Шинжлэх Ухааны Сургууль, 08826

[3]Герман улсын Енна хот дахь Макс-Планкийн Хүн Төрөлхтний Түүхийн Шинжлэх Ухааны Хүрээлэнгийн Археологийн тэнхим, 07745

[4]АНУ-ын Колорадо мужийн Боулдер дахь Колорадогийн Их Сургуулийн Антропологийн тэнхим, 80309

[5]АНУ-ын Мичиган мужийн Анн Арбор дахь Мичиганы их сургуулийн Антропологийн тэнхим, Боловсролын Байгууламжийн Сургууль 3010, Их Сургуулийн Өргөн чөлөө, 610E, 48109

[6]Герман улсын Бонн хот дахь Бонныл Райнише Фридрих-Вилхэлмсийн Их Сургуулийн Археологи, Антропологийн тэнхим, 53113

[7]Монгол улсын Улаанбаатар хот дахь Шинжлэх Ухааны Академийн Археологийн Хүрээлэн, 14200

[8]Монгол Улсын Улаанбаатар хот дахь Шинжлэх Ухаан, Технологийн Их Сургууль

[9]Монгол улсын Улаанбаатар хот дахь Улаанбаатарын Их Сургуулийн Археологийн тэнхим, Баянзүрх дүүрэг, 13343

[10]Монгол улсын Улаанбаатар хот дахь Монгол Улсын их Сургуулийн Антропологи, Археологийн тэнхим, 14201

[11]Монгол улсын Улаанбаатар хот дахь Нүүдлийн Соёл Иргэншил Судлалын Олон Улсын Судлалын Хүрээлэн, 14200

[12]Монгол Улсын Улаанбаатар хот дахь Монгол Улсын Соёлын өвийн Үндэсний Төв, 14200

[13]Монгол улсын Улаанбаатар хот дахь Шинжлэх Ухааны Академийн Түүх, Угсаатан Судлалын Хүрээлэн, 14200

[14]Монгол улсын Ховд аймаг дахь Ховд Их сургууль, 84179

[15]Оросын Холбооны Улсын Москва хот дахь Оросын Шинжлэх Ухааны Академийн Археологийн Хүрээлэн, 119991

[16]Оросын Холбооны Улсын Владивосток хот дахь Оросын Шинжлэх Ухааны Академийн Алс Дорнод дахь салбарын Түүх, Археологи, Угсаатан судлалын Хүрээлэн, 690001

[17]Оросын Холбооны Улсын Улаан-Үүд хот дахь Оросын Шинжлэн Ухааны Академийн Сибирийн салбарын Монгол, Буддизм, Төвд Судлалын Хүрээлэн, 670047

[18]Дээд Боловсрол хариуцсан Улсын Төсөвт Боловсролын Институт “Зүүн Сибирийн Соёл хариуцсан Төрийн Институт”, Нийгэм, Соёлын үйл ажиллагаа, Өв соёл, Аялал Жуулчлалын факультет, Хөгжим, өв соёл судлалын тэнхим, 670031

[19]АНУ-ын Мичиган мужийн Анн Арбор дахь Мичиганы их сургуулийн Антропологийн тэнхим, 48109

[20]Английн Йорк хот дахь Йоркийн Их Сургуулийн BioArCh – Биологи, Археологи, Химийн факультет, Археологийн тэнхим, YO10 5NG

[21]АНУ-ын Массачусетс мужийн Кембридж дэх Харвардын Их Сургуулийн Антропологийн тэнхим, 02138

[†]Дээрх зохиогчид уг судалгааны бүтээлд эн тэнцүү хувь нэмэр оруулсан болно.

*Холбоо барих зохиогч

Холбогдох хаяг
Чунгвон Жүнг
Сөүлийн Их Сургууль, Биологийн Шинжлэх Ухааны Сургууль
Гванак-ро 1, Гванак-гү
08826 Сөүл, БНСУ
+82 2 880 4427
cwjeong@snu.ac.kr

Кристина Вариннер
Макс-Планкийн Хүн Төрөлхтний Түүхийн Шинжлэх Ухааны Хүрээлэн
Калайше гудамж, 10
07745 Енна, ХБНГУ
+49 3641 686 620
warinner@shh.mpg.de

Хураангуй

Зүүн Евразийн тал нутаг нь Хүннү болон Их Монгол улс гэх мэт нүүдлийн мал аж ахуйтай түүхэн эзэнт гүрнүүдийн өлгий нутаг байсан билээ. Гэсэн хэдий ч, энэ бүс нутгийн хүн амын түүх маш бага судлагджээ. Энэхүү судалгаагаар бид 6000 жилийг хамрах 214 антропологийн олдворын өмнө нь судлагдаж байгаагүй, генетикийн-дэлгэрэнгүй шинэ өгөгдлүүдийг шинжлэн үзсэнээр энэ бүс нутгийн хувьсан өөрчлөгдөх генетикийн түүхийг нээн илрүүлж байгаа билээ. Бид судалгаагаараа бэлчээрийн мал аж ахуй нь Монгол нутагт НТӨ 3000 жилийн тэртээ тархан дэлгэрсэн болохыг олж тогтоон, хүрэл зэвсгийн хожуу үе гэхэд Монгол орны хүн ам биологи-газарзүйн хувьд гурван тусдаа бүлэгт хуваагдаж байсныг нь илрүүлсэн ба тэдгээр бүлгүүд нь гарал үүслээсээ үл хамааран, бүгд цагаан идээнд түшиглэсэн бэлчээрийн мал аж ахуйтай байжээ. Хүннү гүрэн тэдгээр аймаг, бүлгүүдийн нэгдэл болоод ойролцоох бүс нутгуудын ард түмний нэгдлүүдээс үүсжээ. Харин, Их Монгол Улс гарал үүслийн хувьд зүүн Евразид илүү хамаарагдах ба өнөөгийн монгол хэлт ард түмэнтэй ижилсэж буй юм. Бид судалгааны үр дүнгээрээ Зүүн бүсийн Тал нутаг дахь генетик, нийгэм-улс төр болон соёлын өөрчлөлтийн дүнд үүссэн нарийн уялдаа холбоог тайлбарлан харуулж байна.

Түлхүү үгс

Хүн амын түүх, эртний ДНХ, нүүдэл, нүүдлийн мал аж ахуй, Зүүн бүсийн тал нутаг, Монгол, Хүннү гүрэн, Монголын эзэнт гүрэн

Удиртгал

Сүүлийн үеийн палеогеномын судалгаагаар Евразийн тал нутаг дахь хүн амын динамик түүх болох Баруун бүсийн Тал нутаг дахь тив-дамнасан нүүдэл нь Европ, Ойрхи Дорнод, Кавказын хүрлийн үеийн хувьсал өөрчлөлтийн үетэй давхцаж байгааг илрүүлжээ (Allentoft et al., 2015; Damgaard et al., 2018a, 2018b; Haak et al., 2015; Mathieson et al., 2015; Wang et al., 2019). Хэдийгээр Баруун бүсийн тал нутаг дахь генетикийн эртний түүхийг ойлгоход ахиц гарсан хэдий ч Зүүн бүсийн тал нутгийн эртний түүхийн хүн амын динамикийн тухай ойлголт бүрхэг хэвээр үлдэж байгаа юм (Damgaard et al., 2018a; Jeong et al., 2018) (Rogers, PhD thesis, Indiana University, 2016). Зүүн бүсийн тал нутаг нь баруун талаараа Алтай- Саяны нуруунаас, зүүн талаараа Зүүн хойд Хятад хүртэл 2500 гаруй км үргэлжлэх өвст тал, ой хээрийн бүс болон говь цөлийг хамарсан өргөн уудам нутаг билээ (Зураг 1; Зураг S1). Энэ бүс газарт өнөөгийн Хятад болон Оросын нутаг дэвсгэрийн зарим хэсгийг хамруулдаг ч ихэнх хэсэг нь өнөөгийн Монгол улсын хил дотор оршдог. Сүүлийн үеийн палеогеномын судалгаануудад зүүн Евразийн ойт хээрийн бүсийн хүн амын генетик бүтэц хүрлийн өмнөх болон хүрлийн түрүү үеүдэд бүрэлдсэн бөгөөд хүчтэй өрнө-дорнын хольц бухий өвөг нь төв Казахстаны Ботайгаас өмнөд Сибирийн Байгал нуур, Оросын Алс Дорнод дахь Чөтгөрийн Гарц агуй хүртэл үргэлжилсэн гэсэн санааг дэвшүүлжээ (Damgaard et al., 2018a; Jeong et al., 2018; Sikora et al., 2019; Siska et al., 2017).

Хүрэл зэвсгийн үед, олон үе шаттайгаар өрнөсөн бэлчээрийн мал аж ахуй нь Зүүн бүсийн тал нутгийн амьдрах хэв маяг, амь зуулгын хэлбэрийг асар ихээр өөрчилсөн байна (Honeychurch, 2015; Kindstedt and Ser-Od, 2019). Саяхны өргөн хүрээг хамарсан палеопротеомик судалгаагаар Монгол оронд НТӨ 2500 оноос өмнө Афанасьев (ойролцоогоор НТӨ 3000 он) болон Хэмцэг (Чемурчек) (НТӨ 2750- 1900)-ийн соёлд хамаарагдах эртний хүмүүс сүү хэрэглэж байсныг баталсан юм (Wilkin et al., 2020a).

Хэдийгээр Дээд Енисей бүс нутгийн Афанасьевийн аймгууд генетикийн хувьд Понти-Каспийн тал нутгийн (ойролцоогоор НТӨ 3300-2200) (Allentoft et al., 2015; Morgunova and Khokhlova, 2013; Narasimhan et al., 2019) Ямная соёлтой холбоотой боловч Хэмцэгийн соёлын гарал маргаантай байгаа юм (Kovalev, 2014). Хүрлийн дунд болон хожуу үед хивэгч амьтны сүүг өргөн хэрэглэх болсон бөгөөд (энд тодорхойлогдсоноор НТӨ 1900-900 он) нутгийн баруун болон умард бүсэд хиргисүүр- буган хөшөөний соёлд (ХБХС), нутгийн зүүн хэсэгт Улаан зуухын соёлд нэвтэрчээ (Jeong et al., 2018; Wilkin et al., 2020a). Буган хөшөө, хиргисүүрийн соёл болон Улаан зуухын ард түмнүүдийн хоорондын холбоо хамаарлын асуудал өнөө хир тодорхой бус байгаа бөгөөд Монголын хүрлийн дунд болон хожуу үеийн Мөнххайрхан, Байтаг гэх мэт бусад оршуулгын дурсгалуудын тухай мэдээлэл ч ховор юм (Хавсралт материалыг үзнэ үү). НТӨ эхний мянганы дунд үе гэхэд өмнөх хүрлийн дунд, хожуу үеийн соёлууд уналтад орж, төмрийн түрүү үеийн соёлууд үүсэн бий болов. Үүнд, Монголын зүүн болон өмнөд бүсийн дөрвөлжин булшны соёл (ойролцоогоор НТӨ 1000-300 он) (тэдгээр дөрвөлжин булшны оршуулгуудаас зарим тохиолдолд ХБХС-ын зүйлсийг ч илрүүлж байв) (Fitzhugh, 2009; Honeychurch, 2015; Tsybiktarov, 2003; Volkov, 2002), мөн баруун хойд бүс Саяны нуруунд Сагил/Уюкын соёл (мөн Сагил-Бажийн соёл, Чандманий соёл ч гэж нэрлэгддэг) (ойролцоогоор НТӨ 500-200 он), (энэ соёл нь Зүүн Казахстаны Сака (ойролцоогоор НТӨ 900-200) болон Алтайн Пазырык (ойролцоогоор НТӨ 500-200) соёлуудтай нягт холбогддог) (Savinov, 2002; Tseveendorj, 2007) зэрэг болно.

НТӨ I мянганы хожуу үеэс эхлэн, Зүүн бүсийн тал нутагт анги давхарга бүхий, төвлөрсөн зохион байгуулалттайХүннү (НТӨ 209-НТ 98), Түрэг (НТ 552-742), Уйгур (НТ 744-840), болон Хятан (НТ 916-1125)–ы эзэнт гүрнүүд сэргэн мандав. Хүннү гүрэн нь умард Хятад, өмнөд Сибирь болон Төв Азийн гүнд хүртэл өргөжин тэлсэн бөгөөд Евразийн хүн ам зүй, геополитикт гүнзгий нөлөөлсөн тал нутгийн анхны төрт улс байв. НТ 13-р зуунд байгуулагдсан Монголын эзэнт гүрэн нь хамгийн сүүлийн бөгөөд дээрх засаглалуудаас хамгийн том газар нутгийг хамарсан, Хятадаас Газрын дундад тэнгис хүртэлх худалдааны замуудыг ч хяналтдаа байлган, өргөн уудам нутаг дэвсгэрийг захирсан том гүрэн байв. Гэсэн хэдий ч өргөн хүрээг хамарсан генетикийн судалгаа дутмаг байгаагаас үүдэн эдгээр эзэнт гүрнүүдийг байгуулсан эрх баригч угсаатнууд болон жирийн ард иргэдийн харилцан хамаарал, гарал үүсэл тодорхойгүй байна (Хавсралт материалыг үзнэ үү).

Түүхийн нэн эрт үеэс нааших Зүүн бүсийн тал нутаг дахь хүн амын динамикийг илрүүлэхийн тулд, бид ойролцоогоор 6000 жилийг (ойролцоогоор НТӨ 4600 – НТ 1400) хамрах, Оросын 3, Монголын 85 дурсгалт газраас илрүүлэн олсон 214 антропологийн олдворын генетикийн дэлгэрэнгүй мэдээллийн санг үүсгэж, анализ хийв (Хүснэгт 1; Хүснэгт S1-S9 болон Зураг. S2-S8) (Нэмэлт мэдээллүүдийг үзнэ үү). Үүн дээр нэмээд, бид саяхан хэвлэгдсэн, Монголын Хойд хэсгээс олдсон хүрлийн үеийн 19 хүний генетикийн мэдээллүүд (Jeong et al., 2018) Орос болон Казахстанд байсан хөрш зэргэлдээ эртний хүн амын мэдээллүүдийг ч (Damgaard et al., 2018a, 2018b; Narasimhan et al., 2019; Sikora et al., 2019; Unterländer et al., 2017) (Хүснэгт S10) мөн оруулсан бөгөөд эдгээр эртний улсуудын генетикийн мэдээллүүдийг дэлхий дахины орчин үеийн хүн амуудтай хамтатган анализ хийсэн (Хүснэгт S11; Зураг. S4). Мөн бид 30 шинэ хурдасгуур масс-спекрометрийн он цаг (Хүснэгт S12), өмнө нь нийтлэгдэж байсан Монголоос олдсон 74 олдворын цацраг-нүүрстөрөгчөөр тогтоосон он цаг (Jeong et al., 2018; Taylor et al., 2019), мөн он цагийг нь шууд тогтоосон олдворуудыг нэмж, нийт 98 хүний (нийт 104 он цаг) он цагийн мэдээллүүдийг энэхүү судалгаагаар боловсруулав (Хавсралт материалыг үзнэ үү).

Үр дүн

Хүрэл зэвсгийн өмнөх үеийн хүн амын бүтэц, бэлчээрийн мал аж ахуйн эхлэл

Энэхүү судалгаагаар, бид он цагийн хувьд НТӨ V болон IV мянганы үед хамаарах 3 дурсгалт газраас олдсон хүрлийн өмнөх үеийн 6 антропологийн олдворыг судаллаа. Үүнд: зүүн Монголоос нэг (SOU001, “eastMongolia_preBA” гэсэн тэмдэглэгээтэй, НТӨ 4686-4495), төв Монголоос нэг (ERM003, “centralMongolia_preBA”, НТӨ 3781-3639) Байгал нуурын зүүн бүс нутгаас олдсон 4 олдвор (“Fofonovo_EN”) зэрэг болно. Эдгээр олдворуудын геномыг өмнө нь хэвлэгдсэн эртний болоод орчин үеийн Евразийн мэдээллийн сантай харьцуулснаар (Зураг 2) (Арга зүйн хэсэг болон хавсралт дахь нэмэлт мэдээллүүдийг үзнэ үү), тэдгээр нь баруун Байгаль (“Baikal_EN”, НТӨ 5200-4200) болон Оросын Алс Дорнодын (“DevilsCave_N”, ойролцоогоор НТӨ 5700) түүхэн тухайн үеийн анчин-түүвэрлэгчидтэй хамгийн ойр холбоотой болохыг олж тогтоосон ба, энэ нь уг генетик профилийн тархалт дахь газарзүйн хоосон орон зайг нөхөж байгаа билээ.

Бид энэхүү генетик профилийг “Эртний Зүүн Хойд Ази” (ANA) хэмээсэн нь түүний газар зүйн тархалтыг Сибирийн Мальта (1950 оноос урагш, ойролцоогоор 24500-24100 жил) ба Афонтын уул (1950 оноос урагш, ойролцоогоор 16900-16500 он)-ын дурсгалт газруудын плейстоцений үеийн анчин-түүвэрлэгчид (Fu et al., 2016; Raghavan et al., 2015) болон Казахстаны Ботайн адуучин ард түмнүүдэд (Damgaard et al., 2018a) тодорхойлогддог өөр нэгэн “Эртний Хойд Еврази” хэмээх өргөн тархсан, Холоцений дунд үеийн генетик профильтой харьцуулан харуулахыг зорьсон болно. Үндсэн бүрэлдэхүүний дүн шинжилгээгээр (PCA), Эртний Зүүн Хойд Ази (ANA) –ийн хүмүүс зүүн хойд Ази дахь өнөөгийн тунгус-нивх ард түмний кластерт дөхөж очиж байгаа нь тэдний генетик профиль өнөөгийн Алс Дорнодын уугуул хүн амын дунд одоог хүртэл байгааг илтгэж байна (Зураг. S3A). Дорнод Монголын хүрлийн өмнөх үеийн хүн ам (eastMongolia_preBA) нь генетикийн хувьд Оросын Алс Дорнод (DevilsCave_N) (Зураг. 3a, 4A S4A; Хүснэгт S5A)–оос ялгарахааргүй бол Байгал нуурын зүүн бүс нутаг (Fofonovo_EN), арай хожуу үеийн Төв Монголын хүрлийн өмнөх үе (centralMongolia_preBA) –ийн хүн амын аль аль нь бага хувиар (12-17%) Эртний Хойд Евразид (ANE) хамаарах (Ботай маягийн) өвгөөс, үлдсэн дийлэнх хувь (83-87%) нь Эртний Хойд Азийн (ANA) өвгөөс гаралтай байна (Зураг. 3A, 4A; Хүснэгт S5A). Байгал нуурын баруун бүсийн түрүү неолитын үеийн Китойн соёлын (Baikal_EN) болон түрүү хүрлийн үеийн Глазковын соёлын (Baikal_EBA) өмнө нь хэвлэгдсэн мэдээллүүдийг (Damgaard et al., 2018a) дахин шинжилж үзэхэд тэдгээр соёлууд ижилхэн угсаа гаралтай бөгөөд түүхийн цаг хугацааны явцад Эртний Хойд Евразийн (ANE) хольц бага багаар (6.4%-с 20.1% хүртэл) нэмэгдсэн болохыг олж тогтоов (Зураг. 3a).

Бэлчээрийн мал аж ахуй нь Саяны нуруу, дээд Енисей мөрнөөр дамжин Монголын баруун хойд бүс нутагт эсвэл Монголын баруун хэсэгт байх Алтайн нуруугаар орж ирсэн Баруун бүсийн тал нутгийн (Афанасьево г.м.) соёлын зүүн тийш чиглэсэн нүүдлийн нөлөөгөөр Монгол нутагт анх орж ирсэн хэмээн голдуу таамагладаг (Janz et al., 2017). Хэдийгээр Афанасьевийн үеийнх хэмээн он цагийг нь тогтоосон булшнуудын дийлэнх нь Алтайн нуруу болон дээд Енисейн хавиас олдох боловч, төв Монголын Хангайн уулсын урд байрлах Шатар чулууны хүрлийн түрүү үеийн дурсгалуудаас Баруун Евразийн митохондрийн хаплогрупп (Rogers et al., 2020) болон хивэгч амьтны сүү хэрэглэж байсан болох протеомик нотолгоо бүхий (Wilkin et al., 2020a) Афанасьевийн маягийн булшнууд мөн олдож байгаа юм. Дээрх дурсгалт газраас илрүүлсэн хоёр хүний дээжид (Afanasievo_Mongolia, НТӨ 3112-2917 cal.) шинжилгээ

хийж үзэхэд, тэдний генетик профиль өмнө хэвлэгдсэн Енисей мөрний сав газрын Афанасьевийн оршин суугчдаас ялгагдахгүй байгаа (Allentoft et al., 2015; Narasimhan et al., 2019) (Зураг 2, Зураг S5C, Хүснэгт S5B) нь хүрлийн түрүү үеийн Баруун бүсийн тал нутгийн мал аж ахуйтан хүмүүс Алтайн нурууг даван зүүн тийш 1500 км цаашлан Монголын төв хэсэг хүртэл нүүдэллэсэн болохыг баталж байгаа юм (Зураг 3a).

Афанасьевчуудын дараа хүрлийн түрүү үед гарч ирсэн соёл бол Хэмцэгийн соёл (НТӨ 2750-1900) юм. Тэд бол оршуулгын байгууламжиндаа хавтан чулуу ашиглаж, хүн дүрст чулуун хөшөөг дагалдуулдаг, мөн хивэгч амьтны сүүг амь зуулгадаа хэрэглэж байсан мал аж ахуйт ард түмэн (Wilkin et al., 2020a) байсан бөгөөд тэднийг мөн Баруун бүсийн тал нутгийн малчдын нүүдэлтэй холбоотой хэмээдэг (Kovalev and Erdenebaatar, 2009). Хэмцэгийн булшнууд Алтай болон Хятадын Шинжаанийн Зүүн гарын сав газраар өргөн тархсан бий (Jia and Betts, 2010; Kovalev, 2014, 2015). Бид Алтайн өврийн Ягшийн хөдөөгийн дурсгалт газраас олдсон Хэмцэгийн соёлын хоёр хүний олдвор болон Алтайн арын Хуурай говь-2 (KUR001), Хөндий говийн (KUM001) дурсгалт газруудаас олдсон хоёр хүний олдворуудыг судаллаа. Монголын Афанасьевийн маягийн булшнуудын (Afanasievo_Mongolia) олдвортой харьцуулбал, Ягшийн хөдөөгийн антропологийн олдворууд Баруун бүсийн тал нутгийн угсаа гарлын шинж өндөр байгаа боловч үндсэн бүрэлдэхүүний дүн шинжилгээгээр (PCA) (Зураг 2) бол Афонтын уул (AfontovaGora3 -AG3) Баруун Сибирь (West_Siberia_N) болон Ботай гэх мэт Эртний Хойд Евразийн оршин суугчидтай генетикийн хувьд хүчтэй хамааралтай байгаа юм. (Зураг. 3a; Зураг. S5A, C). Ягшийн хөдөөгийн Хэмцэгийн антропологийн олдворууд (Chemurchek_southAltai) генетикийн хувьд Зүүн Казахстаны түүхэн тухайн үеийн хүмүүс болох Дали (Dali_EBA)-тай (Fig. 3a) адил болохыг бид олж тогтоолоо (Narasimhan et al., 2019). Ягшийн хөдөө болон хүрлийн түрүү үеийн Дали (Dali_EBA)–ийн олдворуудын генетик профиль нь хоёр хэмжээст холимог загварын аргаар (two-way admixture models) тооцвол Ботай (60-78%) болон эртний Иранаас гаралтай Зүүн өмнөд Узбекистан дахь Бактри-Маргианы археологийн цогцолборын хүрлийн түрүү үеийн гол дурсгал болох Гонур тепегийн (Gonur1_BA) бүлгүүдтэй (22-40%) нилээд таарч байна (Зураг. 3A; Хүснэгт S5B). Уг генетик профиль дэх Афанасьевийн гаралтай багахан хувийг үгүйсгэж болохгүй ч, Иран гаралтай угсааг бүх загваруудад тохируулан үзэх шаардлагатай ба *DATES* системийг ашиглан загварчлахад энэхүү генетикийн хольцолдоо 12±6 (~336±168 жил; Зураг. S6) үеийн өмнө тохиолдсон байх таамаглал гарч байна (Narasimhan et al., 2019). Гэхдээ энэхүү загварчлалд ашиглагдсан бүх төлөөлөх эх үүсвэр хүн ам нь хүрлийн түрүү үеийн Алтайгаас он цаг болон орон зайн хувьд нэлээн хол учир Хэмцэгчүүдтэй ойролцоо хүн амыг яг нарийн тодорхойлох боломжгүй байна. Алтайн арын Хэмцэгийн (“Chemurchek_northAltai”) антропологийн 2 олдворын генийн дийлэнх хувь (~80%) Эртний Хойд Азиас гаралтай ба үлдсэн хувь нь Алтайн өврийн Хэмцэгийн оршин суугчдынхтай төсөөтэй байна (Зураг. 3A, 4A; Хүснэгт S5B). Үүнээс үзвэл, газар зүйн байрлалаас хамааран Хэмцэгийн хүмүүс генетикийн хувьд олон янз байсан ажээ.

Бага хэмжээний геномын шинжилгээн дээр үндэслэсэн хэдий ч, бид Афанасьевын болон Хэмцэгийн соёлын аль аль нь түүхэн дараагийн үе болох хүрлийн дунд болон хожуу үед генетикийн тогтвортой ул мөрийг үлдээгээгүй хэмээн үзэж байна. Энэ нь Европын хүрлийн түрүү үеийн тал нутгийн малчдын нүүдэл уугуул нутгийн хүн амыг ихээхэн өөрчилж, генетикийн хувьд удаан үргэлжилсэн хүчтэй нөлөө үлдээснээс эрс өөр байна. (Allentoft et al., 2015; Haak et al., 2015; Mathieson et al., 2018). Зүүн бүсийн тал нутаг дахь хүрлийн түрүү үеийн малчдын генетикийн түр зуурын нөлөөтэй харьцуулахад тэдний үлдээсэн соёл, аж ахуйн хэлбэр маш хүчтэй, тогтвортой нөлөө үзүүлж хүрлийн түрүү үеийн мал ахуйтнуудын анхлан нутагшуулсан хүнээ

оршуулахдаа дараас байгууламж үйлдэх, цагаан идээнд түшиглэсэн мал аж ахуй эрхлэх зэрэг соёлын онцлог чанарууд өнөөг хүртэл хадгалагдан иржээ.

Хүрэл зэвсгийн үеийн гурван талт генетик бүтцийн үүсэл

Өмнө нь бид хүрлийн түрүү үеийн Байгал нуурын баруун бүсийн (Baikal_EBA) анчин-түүвэрлэгчид болон умард Монголын хүрлийн хожуу үеийн (Khövsgöl_LBA) мал аж ахуйтан ард түмнүүд адилхан генетик профильтой тухай мэдээлсэн билээ (Jeong et al., 2018). Энэхүү генетик профиль нь Эртний Хойд Азийн бүрэлдэхүүнийг ихээр, харин Эртний Хойд Евразийн бүрэлдэхүүнийг бага хэмжээгээр агуулсан бөгөөд мөн энэ судалгааны судлагдахуун болж буй Байгал нуурын зүүн бүс (Fofonovo_EN), төв Монголын хүрлийн өмнөх үеийн (centralMongolia_preBA) бүлгүүд бас ийм генетик профильтой байгаа нь (Зураг. 3А-В, 4А) уг генетик профиль бараг гурван мянганы турш тухайн бүс нутагт оршин тогтносон болохыг илтгэж байна. Умард Монголд төвлөрсөн энэ генетик профиль хүрлийн үеийн бусад бүлгүүдээс нэлээд ялгаатай байв. Бид хүрлийн сүүл үед Монголд газар зүйн бүсээр тодорхойлогдсон бие биеэс ялгаатай гурван өөр генийн сан байсныг илрүүлсний нэг нь Хөвсгөлийн хүрлийн сүүл үеийн хүн ам (Khövsgöl_LBA) (Зураг. 3В, 4А) болох бөгөөд харин нөгөө хоёр хүрлийн дунд, хожуу үеийн Алтай (Altai_MLBA) ба Улаан зуух, Дөрвөлжин булшны соёлууд (Ulaanzuukh_SlabGrave)-ын талаар доор тайлбарлалаа.

Хөгжингүй болон хожуу хүрлийн үед (НТӨ 1900-900 он) цаг уурын өөрчлөлтийн улмаас бэлчээр нутаг өргөжин тэлснээр шинэ бэлчээрийн мал аж ахуй эрхлэгчид уулын бүсээс гарч Зүүн бүсийн тал нутгаар тархан суурьших болсон байна (Kindstedt and Ser-Od, 2019). Энэ үед өнөөгийн айраг исгэх, архи нэрэх уламжлалын (Bat-Oyun et al., 2015) эхлэл тавигдаж гүүний сүүг уг бүс нутагт анх хэрэглэх болсон (НТӨ 1200 он; Wilkin et al., 2020a) бөгөөд тал нутгийн алслагдмал газарт хүрч очих бололцоог үлэмж хэмжээгээр нэмэгдүүлсэн морийг унаж эдлэх, ашиг тусыг хүртэх явдал асар хурдтай эрчимжжээ. Алтай-Саяны бүс нутагт ХБХСоёлын болон бусад тодорхой ангилалд багтаагүй хөгжингүй ба хожуу хүрлийн үеийн оршуулгын төрлүүд (Altai_MLBA, n=7)-тэй холбогдох сүү, цагаан идээнд түшиглэсэн бэлчээрийн мал аж ахуйтнууд Хөвсгөлийн хүрлийн хожуу үеийн хүн ам (Khövsgöl_LBA) ба Синташта удамтай Баруун бүсийн тал нутгийн нүүдэлчид хоёрын холимог генетик тогтолцоог харуулж байна (Зураг 3В, S4B). Ерөнхийдөө, тэдгээр нь үндсэн бүрэлдэхүүний дүн шинжилгээний (PCA) зураглал дээр Баруун бүсийн тал хээрийн хүн ам ба Хүрлийн түрүү үеийн Байгал нуур (Baikal_EBA)/Хүрлийн хожуу үеийн Хөвсгөл (Khövsgöl_LBA) гэсэн кластеруудийн хооронд “Altai_MLBA” хэмээх бүлгийг үүсгэж байгаа (Зураг 2) бөгөөд Баруун бүсийн өвгийн хэмжээнээс хамааран Үндсэн бүрэлдэхүүн 1 (PC1) дээр ялгаатайгаар байрлаж байна (Хүснэгт S5C).

Энэ нь өмнөх үеийн Афанасьев ба Хэмцэгийн хүн амд байсан Баруун бүсийн өвгийн удмынхнаас ялгаатай болovч харин Европын Байлдааны сүхний соёлт (Corded-Ware culture) хүн ам болон Синташта гэх мэт сүүл үеийн Андроновын бүлгүүдтэй ойрын холбоотой Синташта маягийн өвгийн (өмнөх судалгаануудад хүрлийн дунд, хожуу үеийн тал нутаг “steppe_MLBA” хэмээн дурдагдсан) Зүүн бүсийн тал нутаг дахь анхны илрэл болж байгаа юм (Allentoft et al., 2015). Ховд аймгаас олдсон ХБХС (BER002)

болон тодорхой ангилалд ороогүй хүрлийн дунд, хожуу үеийн бүлгүүдэд (SBG001, тус бүрдээ) холбогдох антропологийн олдворуудын генетик профиль хоорондоо төстэй байгаа бөгөөд үүнийг хүрлийн хожуу үеийн Хөвсгөл болон Синташтагийн эн тэнцүү холимог хэмээн загварчлах нь хамгийн оновчтой болохоор байна (Зураг. 3B; Хүснэгт S5C). Энэхүү генетик профиль нь өмнөх судалгаанд хүрлийн хожуу үеийн Хөвсгөлийн кластераас хэлбийсэн, умард Монголын генетикийн нэг салбар хэмээн тодорхойлогдсон профильтой таарч байна (ARS026; (Jeong et al., 2018)). ХБХС болон (ULI001) ангилагдаагүй хүрлийн дунд, хожуу үеийн бүлэгт (BIL001, ULI003, ULZ001) хамаарах, Алтайгаас олдсон нэмэлт дөрвөн антропологийн олдворууд энэ холимог генетикийн загварт янз бүрийн хольцын харьцаатайгаар тохирч байна (Хүснэгт S5C). Нийтэд нь авч үзвэл, хүрлийн дунд, хожуу үеийн Алтайн хүн ам нь (the Altai_MLBA cline) Синташта/Андроновод хамаарах Баруун бүсийн тал нутгийн хүн ам болон Хүрлийн хожуу үеийн Хөвсгөлөөр төлөөлүүлсэн уугуул оршин суугчид гэсэн хоёр хүн амаас эх үүсвэртэй үргэлжилж буй холимог генетик бүрдлийг харуулж байна. Уг холимог генетикийн бүрдэл нь энэ судалгаанд шинжилсэн олдвороос ердөө 10±2 үе (~ 290 жил)-ийн өмнө тохиолдсон хэмээн тооцоолол гарсан нь олон гарал үүсэлтэй удамшлын харьцаатайгаа таарч байгаа юм (Зураг S6). Синташтагийн соёл (НТӨ 2200-1700 онд) нь морь хөллөсөн дугуйт тэрэг зэрэг тээврийн шинэ технологитой холбогддог (Anthony, 2010) учир Зүүн бүсийн тал нутагт энэхүү угсаа гарч ирсэн явдал нь өндөр үр ашиг бүхий, хөдөлгөөнт тээврийн хэрэгсэл Евразийн тал хээрийн олон үндэстнүүдийг хооронд нь холбоход чухал үүрэг гүйцэтгэсэн болохыг харуулж байна (Honeychurch, 2015). Манай мэдээллийн санд байгаа хүрлийн дунд, хожуу үеийн антропологийн гурван олдворын генетик профилийг Хүрлийн дунд, хожуу үеийн Алтайн бүлгээр тайлбарлаж болохооргүй байна. Эдгээр гурван олдвор - Алтайн хоёр (UAA001 and KHI001), Хөвсгөлийн нэг UUS001 олдворуудыг гуравдагч эх үүсвэр хүн ам болох Gonur1_BA-ийн бага зэргийн хольцтой хэмээн загварчлах нь илүү тохиромжтой байна (Хүснэгт S5C). Бүхэлд нь авч үзвэл, Алтай болон умард Монголын хүрлийн хөгжингүй болон хожуу үеийн оршуулгын томоохон уламжлалуудын (Мөнххайрхан, ХБХС болон ангилагдаагүй дурсгалууд) хооронд соёлын ялгаа байсан боловч генетикийн хувьд ялгаатай бүлгүүд байгаагүй ажээ.

Хүрлийн дунд, хожуу үеийн Алтайн гетероген хүн амын Алтай-Саяны бүс нутагт төмрийн түрүү үед үлдээсэн үр удмыг бид Баруун хойд Монголын Чандмань уулын Сагил-Уюкийн (“Chandman_IA”, ойролцоогоор НТӨ 400-200 он) дурсгалт газар хийсэн малтлагаас илрүүлсэн. Чандманий 9 антропологийн олдвор үндсэн бүрэлдэхүүний дүн шинжилгээгээр хүрлийн хожуу үеийн Хөвсгөлийн кластераас тусдаа, өмнөх хүрлийн хөгжингүй, хожуу үеийн Алтайн бүлгийн төгсгөлд нягт бөөгнөрөл бүхий кластер үүсгэж байна (Зураг. 2). Төмрийн түрүү үед, Сагил-Уюк (мөн Сагил-Бажийн соёл хэмээгддэг) соёлын ард түмэн нь өнөөгийн Тува буюу Дээд Енисейн бүс нутгаар төвлөрөн бэлчээрийн мал аж ахуй, хар будааны тариалан хослуулан амьдардаг байжээ. Тэд Алтайн Пазырыкууд, зүүн Казакстаны Сака нарын хамт Баруун бүсийн тал нутаг, Таримын сав газар болон Дээд Енисей хүртэл тархсан Скифийн соёлын нэгэн хэсэг болж байжээ (Parzinger, 2006).

Төмрийн түрүү үеийн Скифийн хүн ам нь Хүрлийн дунд, хожуу үеийн Алтайнхаас системтэйгээр хазайж байгаа тул гуравдагч өвгийг тодруулах шаардлага гарч байна (Зураг 3C, 4A, S4C). Төв Азийн (Кавказ/Ираны өндөрлөг газар/Трансоксиана мужууд), түүний дотор Бактри-Маргианы археологийн цогцолбор (BMAC) (Narasimhan et al., 2019) –той холбогдох уг өвгийн шинж нь Төв Сака (Damgaard et al., 2018b), Тяньшаний Сака, Тагар (Damgaard et al., 2018b), Чандмань гэх мэт төмрийн үеийн бүлгүүдэд тодорхой илэрдэг бол харин эртний ХБХС-ын болон Карасук бүлгүүдэд илрээгүй

болно (Хүснэгт S5C-E). Энэхүү генетикийн гурав дахь бүрэлдэхүүн хэсэг нь эдгээр төмрийн үеийн бүлгүүдийн өвгийн 6-24%- ийг эзэлж байгаа бөгөөд төмрийн үеийн Чандманий генийн хольцолдоо ойролцоогоор ~ 18 ± 4 үеийн өмнө буюу НТӨ 750 оны үед тохиолдсон хэмээн тооцоолж байна. Энэ нь НТӨ 1600 оны үед Бактри-Маргианы археологийн цогцолбортой холбогдох соёл мөхсөнөөс хойш, харин НТӨ 550 оны үед Перс Ахеменидын эзэнт гүрэн үүсэхээс бага зэрэг өмнө тохиолдсон гэсэн үг юм (Зураг. S6).

Ирантай холбогдох энэхүү генетикийн урсгал нь хүрлийн хожуу үеэс төмрийн түрүү үед шилжих шилжилтийн үед Трансоксиана (Туран) ба Фергана бүс нутгийн газар тариалан, мал аж ахуй эрхлэгч ард түмнүүдтэй ихээр харилцаж, холилдсоноос үүдэн бий болсон хэмээн үзэж байна. НТӨ II мянганы сүүл, I мянганы эхэн үед морь унах соёл өргөн дэлгэрч (Drews, 2004), улмаар адууг тээвэрт хэрэглэх нь улам бүр боловсронгуй болж өсөн нэмэгдсэн нь хүн амын харилцааг нэмэгдүүлж, Ирантай холбогдох угсаа гарал иймхүү хээр талд тархах нөхцөл болсон бололтой. Түүнчлэн, тэдгээр өвгүүд хүрэл зэвсгийн үеэс эхлэн Дотоод Азийн уулсын хонгил дагуух эрчимт хөдөлгөөн, Шинжаанаар дамжин Алтайд орж ирэх (Frachetti, 2012) өөр сувгууд байсан байж болохыг ч бидний судалгаа үгүйсгэхгүй юм.

Монголын дорнод ба өмнөд хэсгүүдэд Алтай, умард Монголын хүрлийн дунд болон хожуу үе, төмрийн түрүү үеийн соёлуудаас ялгаатай оршуулгын өөр уламжлалууд болох хүрлийн хожуу үеийн Улаан зуух (НТӨ 1450-1150), төмрийн түрүү үеийн дөрвөлжин булшны дурсгалууд (НТӨ 1000-300) олддог (Honeychurch, 2015). Зүүн бүсийн тал нутгийн тухайн үеийн бусад хүн амуудаас ялгаатай нь эдгээр дурсгалуудаас олдсон антропологийн олдворуудад Эртний хойд Еврази (ANE) болон Баруун бүсийн тал нутгийн нүүдэлчдийн (WSH) хольцгүй цэвэр Зүүн хойд Азийн (ANA-д хамаарах) генетик профиль ажиглагдаж байна. (Зураг 2, 3C, 4). Улаан зуух, болон дөрвөлжин булшны соёлт ард түмэн хоёулаа бэлчээрийн мал аж ахуй эрхэлдэг байсан ба төмрийн түрүү үеийн дөрвөлжин булшны соёлтой ард түмэн гүүний сүүг хүнсэндээ ашигладаг байжээ (Wilkin et al., 2020a). Улаан зуух ба дөрвөлжин булшны соёлын хүмүүс генетикийн хувьд ялгаагүй шинж чанартай (Зураг 2, Хүснэгт S5C) байгаа нь дөрвөлжин булшны соёл Улаан зуухын соёлоос гаралтай хэмээх археологийн таамаглалтай нийцэж байна (Honeychurch, 2015; Khatanbaatar, 2019). Уг хоёр соёл хоёулаа хүрлийн өмнөх үеийн дорнод Монголоос олдсон, ойролцоогоор НТӨ 4600 оны үеийн антропологийн олдворын дээжээс (eastMongolia_preBA) ялгарахааргүй байгаа нь дорнод Монголын энэхүү нэн эртний генийн сан маш урт удаан (4,000 жилээс их)хугацаанд тогтвортой оршин байсан болохыг илтгэж байна (Хүснэгт S5C). Удаах шинжилгээгээр бид, Улаан зуух болон дөрвөлжин булшны соёлуудыг нэг генетик бүлэгт (“Ulaanzuukh_SlabGrave”) нэгтгэн судлав. Улаан зуух, дөрвөлжин булшны генетик кластер нь магадгүй өмнө дурдагдсан Хөвсгөл аймгийн ХБХС-ын зүүнш хазайсан бүлэг (ARS017) (Jeong et al., 2018) болоод Төв Монголоос олдсон соёлын хувьд тодорхой ангилагдаагүй, хүрлийн хожуу үеэс төмрийн түрүү үед шилжих үеийнх гэж тодорхойлогдсон антропологийн нэг олдворын (TSI001) эх үүсвэр байж болох талтай байна (Зураг 2, 3B-C; Хүснэгт S5C). Түүнээс гадна, баруун хойд Монголын Мөнххайрханы антропологийн KHU001 олдворт хүрлийн эхэн үеийн Байгалын өвгөөс гадна Улаан зуух, дөрвөлжин булшны гаралтай өвгийн удамшил анзаарахгүй орхиж боломгүй их хувьтай байв (Зураг S4B; Хүснэгт S5C). Дээрх гурван антропологийн олдворууд нь хүрлийн хожуу үеийн дорнод болон баруун хойд Монголын хоорондын ховор тохиолдлын холын харилцааны баталгаа болж байна. Бид Алтайд Улаан зуух, дөрвөлжин булшны өвгийн ул мөр олж илрүүлээгүй бөгөөд хүрлийн дунд, хожуу үеийн Монголын дорнод болон өмнөд хэсгээс гадуурх Улаан зуух, дөрвөлжин булшны генетик профилийн давтамж маш бага

байлаа. Төмрийн түрүү үед дөрвөлжин булшны соёл хойд зүг рүү өргөжин тэлэх замдаа, зарим тохиолдолд өмнөх соёл болох ХБХС-ыг тасалдуулан, зайлуулж байсан (Fitzhugh, 2009; Honeychurch, 2015; Tsybiktarov, 2003; Volkov, 2002) бөгөөд эцэст нь хамгийн хойд цэг нь Байгал нуурын зүүн хэсэгт хүрчээ. Үүнийг уг судалгаан дахь дөрвөлжин булшны PTO001 олдворын генетик профилоос харж болно (Зураг 3C). Манай судалгааны үр дүн хүрлийн үеийн Зүүн бүсийн тал нутгийн хүн амд төмрийн түрүү үеийн төгсгөл хүртэл зүүн ба баруун бүсийн генетикийн ялгарал байсныг илрүүллээ. Цаашид Монголын төв болон өмнөд бүс нутгийн дээжний судалгаа эдгээр удамшлын төлөв байдлын орон зайн тархалтыг болон бидний одоогийн олж илрүүлээд буй үр дүнгүүдийг улам гүнзгийрүүлж, сайжруулахад туслах болно.

Хүннү гүрэн, тал нутгийн анхны төрт улсын үүсэл

Зүүн бүсийн тал хээрийн эртний ард түмнээс үүсэн бий болсон томоохон хэмжээний төрт улсууд НТӨ нэгдүгээр мянганы сүүл үеэс хөгжин цэцэглэж эхэлжээ. Хүннү нь талын нүүдэлчин ард түмний байгуулсан, анхны түүхэн сурвалжид тэмдэглэсэн эзэнт гүрэн байсан бөгөөд үүнийг Зүүн бүсийн тал нутаг дахь нийгэм-улс төрийн хувьд түүхэн үйл явдал болсон хэмээн үздэг (Brosseder and Miller, 2011; Honeychurch, 2015). Хүннү нар НТӨ III зуунаас НТ I зуун хүртэл Зүүн ба Төв Азийн өргөн уудам нутаг дэвсгэрт өөрсдийн улс төрийн ноёрхлыг тогтоож байв. Хүннү гүрнийг байгуулсан ард түмний соёл, хэл шинжлэл, удам угсааны бүрэлдэхүүн мөн тэдний Зүүн бүсийн тал нутаг дахь дараа дараагийн бусад нүүдэлчид, орчин үеийн хүн амын бүлгүүдтэй харилцах харилцаа зэрэг нь эрдэм шинжилгээний нэн чухал сэдэв байсаар ирсэн. Энэ судалгаагаар бид НТӨ 200 - НТ 100 он хүртэлх Монголын нутгаас илэрсэн Хүннүгийн үеийн 60 антропологийн олдворын генетикийн дэлгэрэнгүй мэдээллийг танилцуулж байна. Тэдгээр олдворуудын ихэнх нь Хүннүгийн сүүл үед (НТӨ 50-иад оноос хойш) хамаарах боловч 13 олдвор НТӨ 100 оноос өмнөх цаг үед холбогдож байв. Тэдгээрийн 12 олдвор нь Салхитын Амны (SKT) болон Ацын голын (AST) Хүннүгийн үеийн дурсгалаас (AST) олдсон бол нэг антропологийн олдвор нь Зүүн Монголд байх Хүннүгийн түрүү үеийн Жаргалантын Ам (JAG) –ын малтлагаас олдсон юм.

Бидний ажигласнаар, Хүннү гүрэн анх бүрэлдэхэд хүн амын ялгаатай хоёр үйл явц оролцжээ. Нэгдүгээрт, антропологийн эрт үеийн олдворуудын тал нь (n=6) түүний өмнөх үеийн Алтай–Саяны бүс орчмын Сагил-Уюкын соёлын Chandman_IA-тэй ижил генетикийн (early Xiongnu west) кластерийг үүсгэж байна (Зураг. 2). Тэд төмрийн үеийн Чандманий хүн амаас 92%-ийн удамшил авсан бол үлдсэн хэсэг нь Иран гаралтай өвгөөс үүдэлтэй ба бид үүнийг Бактри-Маргианы археологийн цогцолбороор төлөөлүүлэн загварчлав (Зураг. 3D, 4D; Хүснэгт S5F). Үүнээс дүгнэвэл, төмрийн түрүү үед Чандмань, Сагил-Уюкын соёлд холбогдох антропологийн олдворуудаас илэрсэн энэхүү бага хэмжээний Ирантай холбогдох генийн тархалт НТӨ I мянганы хоёр дахь хагаст ч мөн үргэлжлэн баруун болон хойд Монголоор тархсан болохыг илтгэж байна. Хоёрдугаарт, 6 антропологийн олдвор (“early Xiongnu_rest”) Хүннүгийн эхэн үе (early Xiongnu_west) болон Улаан зуух-дөрвөлжин булш (Ulaanzuukh_SlabGrave) хоёрын дунджийг илэрхийлж байна. Тэдний 4 нь янз бүрийн хэмжээнд Хүннүгийн эхэн үе (39-75%) болон Улаан зуух-дөрвөлжин булшны (25-61%) өвгийг тээж байгаа бол 2 нь (SKT004, JAG001) Улаан зуух-дөрвөлжин булшны кластераас ялгагдахааргүй байв (Зураг. 3D; Хүснэгт S5F-G). Уг генетик бүлэг нь баруун зүгийн Хүннүгийн түрүү үе (earlyXiongnu_west) болон Улаан зуух-дөрвөлжин булшны (Ulaanzuukh_SlabGrave) генийн санг холбож, Зүүн бүсийн тал нутаг дахь хамгийн их ялгаа, зөрүүтэй хоёр угсаа болох баруун тийшээ ХБХС, Мөнххайрхан болон Сагил-Уюкийн үр удам, харин зүүн тийшээ Улаан зуух, дөрвөлжин булшны соёлын үр хойч нар нэгдэн нийлсэн болохыг

илэрхийлж байгаа юм. Бүхэлд нь авч үзвэл, өмнөх Сагил-Уюкын соёлоос үргэлжилсэн Иранаас гаралтай бага хэмжээний генийн урсгал болоод Зүүн бүсийн тал нутгийн генийн санг нэгтгэн гэнэт гарч ирсэн, зүүн, баруун бүсийн тал нутгийн холимог ген нь Хүннүгийн сэргэн мандалтай холбоотой хүн ам зүйн үйл явцыг тодорхойлох хоёр гол хүчин зүйл болж байгаа юм.

Хүннүгийн сүүл үеийн антропологийн олдворууд дундаас, бид генетикийн гетероген байдлыг илүү их харж болох (Зураг 2) ба Хүннүгийн эхэн үед өрнөсөн хүн ам зүйн хоёр тусдаа үйл явц нь Хүннүгийн сүүл үе хүртэл үргэлжилсэн болохыг харж болох боловч генийн урсгалын шинэ давлагаа, нарийн төвөгтэй чиглэлүүд нэмэгджээ. Хүннүгийн хожуу үеийн 47 антропологийн олдворын тал нь (n = 26) Хүннүгийн эхэн үед ажиглагдсантай ижил холимог генетикийн бүрдлийн загвараар тайлбарлах боломжтой: Эдгээрийн 22 нь төмрийн түрүү үеийн Чандманийн соёл (Chandman_IA) + Улаан зуух, дөрвөлжин булшны соёлын (Улаанзуух_SlabGrave) холимог, хоёр нь (NAI002, TUK002) Чандманийн соёл (Chandman_IA) + Бактри-Маргианы археологийн соёл эсвэл төмрийн түрүү үеийн Чандманийн соёл (Chandman_IA) + Улаан зуух, дөрвөлжин булшны соёл (Ulaanzuuk_SlabGrave) + Бактри-Маргианы археологийн соёл гэсэн аль алиных нь холимог байдлаар буй болж мөн хоёр олдвор (TUK003, TAK001) Хүннүгийн түрүү үе (earlyXiongnu_west) + Улаан зуух, дөрвөлжин булшны соёл (Ulaanzuukh_SlabGrave) эсвэл Хүннүгийн түрүү үе (earlyXiongnu_west) + Хөвсгөлийн хүрлийн хожуу үеийн (Khovsgol_LBA) гэсэн холимогуудаар загварчлагдаж болохоор байна (Зураг 3D, 4D; Хүснэгт S5G).

Цаашлаад дахин хоёр олдворын (TEV002, BUR001) угсаа гарал нь Хүннүгийн удмын сангаас гаралтай байх магадлалтай боловч тэдгээр загваруудын p-утга нь 0.05 гэсэн байх ёстой хамгийн бага босго хэмжээнээс арай бага байгаа юм (Хүснэгт S5G). Мөн хэдий тийм боловч Үндсэн бүрэлдэхүүн-1 (PC1) –д хамаарах, Баруун Евразийн удам хамгийн их харьцааг эзэлж буй Хүннүгийн хожуу үеийн дараагийн 11 олдворыг Бактри-Маргианы археологийн дурсгалтай холбогдох соёл эсвэл Ираны гаралтай бусад эртний хүн амуудын загварыг ашиглан загварчлах боломжгүй байв. Харин оронд нь, тэд баруун бүсийн болон төв тал хээрийн янз бүрийн байршилд байх эртний сарматчуудын кластерт хамаарч байна (Зураг 2).

Холимог генийн бүрдлээр загварчилснаар хожуу хүннүчүүдийн дунд сарматаас гаралтай генийн сан байсныг батлав: гурван антропологийн олдвор (UGU010, TMI001, BUR003) нь сарматын гентэй ижил, хоёр (DUU001, BUR002) нь сармат ба Бактри-Маргианы археологийн цогцолбортой холбогдох соёлын холимог байсан бол гурван олдвор (UGU005, UGU006, BRL002) нь сармат ба Улаанзуух, дөрвөлжин булшийн соёлуудын холимог генийн бүрдэл байв. Харин дахин гурван олдвор (NAI001, BUR004, HUD001) Сарматян, Бактри-Маргианы археологийн дурсгалтай холбогдох соёлын ба Улаан зуух, дөрвөлжин булш гэсэн холимог загвартай таарч байв (Зураг 3D, S4D; Хүснэгт S5G). Нэмээд, Зүүн Евразийн удамшилтай хамгийн их таарч байсан, Үндсэн бүрэлдэхүүн-1 дээр байрлах 8 антропологийн олдвор нь Улаан зуух, дөрвөлжин булшийн соёл болоод хүрлийн сүүл үеийн Хөвсгөлийн аль алиных нь генетик төлөв байдлаас ялгаатай ба Зүүн Ази, цаашлаад Өмнөд Азийн орчин үеийн хүмүүсийн Үндсэн бүрэлдэхүүн -2 -той таарч байв (Зураг. 2). Тэдгээр 8 олдворын 6 (EME002, ATS001, BAM001, SON001, TUH001, YUR001) нь Улаан зуух, дөрвөлжин булшны соёл болон Хань үндэстний (Хүснэгт S5F-G) холимог генетикийн загвартай таарч, ялангуяа генетикийн төлөв байдлыг нь “Han_2000BP” хэмээн бидний тэмдэглэсэн YUR001 дугаартай нэг олдвор өмнө нь судлагдан нийтлэгдэж байсан хоёр Хань гүрний цэрэгтэй (Damgaard et al., 2018b) ойрын холбоо бүхий генетикийн ижилсэлтэй гэсэн дүгнэлт

гарсан юм (Хүснэгт S5G). Үлдсэн хоёр антропологийн олдвор (BRU001, TUH002) нь ижил төстэй генетикийн төлөв байдалтай боловч сарматийн удамшлыг нэмж оруулан, шалгах шаардлагатай болж байна (Хүснэгт S5G). Хүннүгийн сүүл үеийн ард түмэн Хүннүгийн эхэн үеийн ард түмнээс ялгагдах хоёр нэмэлт хүн ам зүйн шинж чанараар тодорхойлогддог: шинэ Сарматаас гаралтай барууны удамшлын генийн урсгал болон өнөөгийн Хятадын Хань гүрний хүмүүстэй холилдон, харилцаа, холбоогоо эрчимжүүлснээс үүдсэн ялгаа гэсэн үг юм. Хүннүгийн үеийн Эгийн голын оршуулгын дурсгалын тухай өмнөх судалгаагаар баруун болон зүүн Евразийн гаралтай митохондрийн хаплогруп байгааг илрүүлсэн (Keyser-Tracqui et al., 2003) ба энэ нь геномын дэлгэрэнгүй мэдээллийн санд буй бидний баруун, зүүн холимог генетикийн дүгнэлтүүдтэй нийцэж байна. Эдгээр дүн шинжилгээнүүд нь Хүннү нар хөрш зэргэлдээ улсууд болох Төв Азийн Торгоны замын хот улсууд ба Хань гүрний Хятад улсад, мөн Монгол руу орж, гарахыг завдсан нүүдэл, суурьшил зэргийг ч хянаж, улс төрийн нөлөө үзүүлж, ноёрхлоо тогтоож байсан тухай түүхэн сурвалжуудтай сайн таарч байна (Miller, 2014). Ер нь Хүннүгийн үеийг баруун, зүүн Монголын удмын санг нэгтгэж, цаашлаад бүр баруун, зүүн Азийн удмын санг нэгтгэн, уудам цар хүрээг хамарсан, өргөн хүрээний генийн урсгалуудын нэг хэмээн тодорхойлж болно.

Хүннүгийн дараах улсуудын генетикийн гетероген байдлын хэлбэлзэл

Ойролцоогоор НТ 100 онд Хүннү гүрэн задран унасны дараа, Зүүн бүсийн тал нутагт хэдэн зууны турш бие биенээ залгамжлан, улс төрийн хувьд хуваагдмал байдалтай хэд хэдэн улс үүсэж бий болсон нь Сяньби (НТ 100-250 он), Нирун (НТ 300-550 он), Түрэг (НТ 552-742 он, Уйгур (НТ 744-840) зэрэг улсууд юм. Хэдийгээр бидний судалсан Дундад зууны эхэн үеийн дээжүүд тэнцвэртэй бус, тодорхой ангилалд ороогүй ч Сяньби эсвэл Нируны үед (TUK001) холбогдох нь тогтоогдсон нэг антропологийн олдвор, Түрэгийн оршуулгын байгууламжаас илэрсэн 8 олдвор, Уйгурын оршуулгын дурсгалаас олдсон 13 олдвороос бүрдэж байгаа юм. Эдгээр антропологийн олдворууд өмнөх Хүннүгийн үеийнхээс өөр генетикийн төлөв байдалтай байгаа нь тодорхой харагдаж буй бөгөөд тухайн үед Монгол нутагт хүн амын миграцийн шинэ үйл явц явагдсан болохыг илтгэж байна (Зураг 2). TUK001 дугаартай антропологийн олдвор Хүннүгийн түрүү үеийн оршуулгын газраас олдсон ч он цагийн хувьд ямар нэгэн хамааралгүй (ойролцоогоор НТ 250-383), генетикийн хувьд баруун Евразийн хүмүүсийн генийн сантай хамгийн ойр байна. Уг генийн сан Сарматчуудынхаас ялгаатай, Иран/Бактри-Маргианы археологийн соёлын амтай илүү ойр байгаа юм (Зураг 2). Зүүн Евразийн хүн амуудтай хамгийн ойр антропологийн олдворууд дунд хоёр Түрэг, нэг Уйгурын үеийн олдвор (ZAA004, ZAA002, OLN001.B) Улаан зуух, дөрвөлжин булшны соёлын кластертай ижил байв. Мөн Тан улстай нэг цаг үед холбогдох Түрэгийн үеийн язгууртны нэгэн тоногдсон булшнаас илэрсэн өөр нэгэн антропологийн олдвор (TUM001) нь Хан үндэстэнтэй холбогдох удамшлын хувь өндөр байв (78%; Зураг 3E, 4B, S4E; Хүснэгт S5H). Хоёр нохой хамт дагалдуулан оршуулсан байсан тухайн эрэгтэй нь бунхны хаалгыг хамгаалахын тулд золиослогдсон Хятад дагуул зарц байсан байж болох талтай (Ochir et al., 2013). Үлдсэн 17 түрэг, уйгарын үеийн хүмүүс дундаж генетикийн төлөв байдлыг илтгэсэн болно (Зураг 3E).

Уйгурын нийслэл Орду-Баликийн ойролцоо байх Уйгурын үеийн Олон довын оршуулгын дурсгалаас олдсон түрүү дундад зууны үеийн 12 антропологийн олдворууд генетикийн хувьд өндөр хувиар гетероген эх үүсвэртэй болох нь тодорхой харагдав (OLN; Зураг 2) Эдгээр олдворуудын зургаа нь зөвхөн нэг булшнаас (булш 19) олдсон ч, тэдгээрийн зөвхөн хоёр нь л удамшлын холбоотой (OLN002 ба OLN003, хоёрдугаар зэрэг; Хүснэгт S7), бусад нь ойр ураг төрлийн холбоо байхгүй байгаа нь ийм булшны

зохион байгуулалт, тэдгээрийн дотор оршуулсан хүмүүсийн нийгмийн харилцааны талаарх асуулт урган гарч байна.

Уйгурын үеийн ихэнх антропологийн олдворуудын Баруун Евразийн удамшил өндөр хувьтай ч мөн хувьсамтгай чанартай байгаа ба Алан (өнөөгийн Хүннү болон Сарматын үр удам байж болох уламжлалт мал ахуй эрхлэгч нүүдэлчид (Bachrach, 1973)) болон Иранаас гаралтай (Бактри-Маргианы археологийн соёл), үүнээс гадна – Улаан зуух, дөрвөлжин булшны соёлын (Эртний Хойд Азитай холбоотой) угсааныхны холимог хэв шинжтэй түлхүү таарч болохоор байна (Зураг 3E). Энэхүү судалгаанд ашигласан эртний түрэг, уйгур угсааныханд хамаарах олдворуудыг он цагийн холимог загвараар тооцоолоход ойролцоогоор НТ 500 оны үетэй таарч байна: Энэ нь Түрэгийн 8 олдворуудаас 2, харин Уйгарын 12 олдворуудаас 2 тус тус Түрэгийн-Уйгарын өмнөх үед холбогдохоор байна (ZAA001 дугаартай антропологийн олдвор болон Олон Довоос олдсон олдворуудыг үүнд төлөөлөл болгон авч үзэв).

Монголын эзэнт гүрний мандалт

9-р зууны дундуур Уйгурын эзэнт гүрнийг унасны дараа Хятадын зүүн хойд хэсэгт байсан Хятанчууд НТ 916 онд хүчирхэг Ляо улсыг байгуулжээ. Хятанчууд Зүүн бүсийн тал нутгийн томоохон хэсгийг эрхшээлдээ байлгаж, эзэлсэн нутагтаа ард түмнийг дахин зохион байгуулалтад оруулж, нүүлгэн шилжүүлж байсан хэмээн тэмдэглэсэн байдаг (Kradin and Ivliev, 2008). Гэвч Монголын нутаг дэвсгэрт Хятаны үеийн оршуулгын дурсгал маш цөөн олдсон байдаг. Манай судалгаанд Булган аймгаас олдсон Хятаны гурван олдвор хамрагдсан (ZAA003, ZAA005, ULA001) бөгөөд бүгд Зүүн Евразийн генетик профильтой (Зураг 2), 10%-аас бага баруун Евразийн өвгийн хольцтой байв (Зураг 3F, 4B; Хүснэгт S5I). Энэ нь монгол хэлт хятанчуудын зүүн хойд Азиас гарал үүсэлтэйг илэрхийлж байж болох ч Монгол дахь Хятаны хүн амын генетик төлөв байдлыг нарийн тодорхойлохын тулд илүү олон тооны дээж шаардлагатай юм. НТ 1125 онд Хятаны эзэнт гүрэн Зүрчидийн Алтан улсад ялагдаж, харин Алтан улс дараа нь НТ 1234 онд монголчуудад эзлэгдэв.

Монголын эзэнт гүрэн (НТ 1206-1368) хамгийн оргил үедээ Евразийн бараг гуравны хоёрыг хамарч байв. Энэ бол дэлхийн хамгийн том хуурай газрын нутаг дэвсгэрийг эзэлсэн эзэнт гүрэн байсан бөгөөд нийслэл хотод нь дэлхийн өнцөг булан бүрээс олон хэлийн үндэстэн цугласан улс байв. Бид Монголын эзэнт гүрний үеийн жирийн иргэд, орон нутгийн жижиг ноёдын булшнаас олдсон 62 антропологийн олдворыг судалсан ба үүнд хаад, язгууртан болон нийслэл хот Хархорины антропологийн олдворууд багтаагүй билээ. Бидний тогтоосноор, Монголын эзэнт гүрэн янз бүрийн угсаа гаралтай хүмүүсээс бүрдэж байсан боловч Хүннүгийн үетэй харьцуулахад генетикийн олон янз байдал нь бага (Зураг 2) бөгөөд Хүннү болон түүнээс өмнөх баруун, хойд хэсгийн хүрлийн дунд, хожуу үеийнхэнд байсан Эртний Хойд Евразиас гаралтай (төмрийн үеийн Чандманийн соёл ба хүрлийн хожуу үеийн Хөвсгөлийнх шиг) генийн үлдэгдэл бараг байхгүй байна. Монголын эрин үеийн антропологийн олдворууд өмнөх улс гүрнүүдээ бодвол Зүүн Евразийн удамшил илүү өндөртэй байгаа бөгөөд энэ үеэс орчин үеийн монголчуудын генийн сан бүрэлдэж эхэлсэн ажээ. Бидний тогтоосноор, ихэнх түүхэн монголчууд Улаан зуух, дөрвөлжин булш, Хань болон Алан гэсэн удамшлын төлөөлөл бүхий гурван талт холимог генетикийн бүрдлийн загварт таарч байна. Үндсэн Бүрэлдэхүүний дүн шинжилгээн дээрх байрлалаас харахад (2-р зураг), Монголын эрин үеийн антропологийн олдворууд Баруун бүсийн тал нутгийн (Алан эсвэл Сарматын) удамшил 15-18%, Улаан зуух, дөрвөлжин булшных 55-64%, харин Хань гаралтай өвгийн удамшил 21-27% -тай байна (Хүснэгт S5I). Олдвор тус бүрт ижил загвар

ашиглан Монголын үеийн 61 олдворын 56-г нь (магадлалын итгэлцүүрийн 0.05 түвшний *p* утганд тулгуурлан), мөн он цагийн хувьд Монголын эзэнт гүрний эхэн үед хамаарах, тодорхой ангилалд багтаагүй дундад зууны сүүл үеийн нэгэн олдворыг ч (SHU002) дээрх гурван өвгийн эх үүсвэр бүхий загвараар оновчтой тайлбарлаж болохоор байна (Хүснэгт S5J).

НТ 1368 онд Монголын эзэнт гүрэн унаснаас хойш Монголын хүн амын генетикийн төлөв байдалд онцын өөрчлөлт ороогүй байна. Монголын эзэнт гүрний үед бүрэлдсэн генетикийн бүтэц нь өнөөгийн Монгол ба Орос улсад оршин сууж буй монгол хэлт ард түмнүүдийг тодорхойлж байгаа юм. Бид түүхэн монголчууд ба өнөөгийн монгол хэлтэй долоон бүлгийн (Монгол, Халимаг, Буриад, Хамниган, Даур, Ту, Монгола) хоорондын генетикийн ижилсэл, уялдаа холбоог *qpWave*-ийн шинжилгээг гүйцэтгэлээ. Бидэнд байгаа өгөгдлийн хүрээнд түүхэн монголчуудын 34/61 нь орчин үеийн монгол хэлээр ярьдаг дор хаяж нэг хүн амтай удамшлын холбоотой байна (Зураг S7B). Монголын эзэнт гүрэн Зүүн бүсийн тал хээрийн улс төр, генетик төрхийг бүрэлдүүлэхэд гүнзгий нөлөөлсөн нь эзэнт гүрний уналтаас хойш ч удаан үргэлжилж, өнөөг хүртэл Монгол оронд оршсоор байна.

Зүүн бүсийн тал нутаг дахь генетикийн бүрдлийн үйл ажиллагааны болон жендерийн асуудлууд

Зүүн бүсийн тал нутаг дахь генетикийн бүрдлийн үйл ажиллагааны асуудлыг судлахын тулд лактоз задлах чадвар (LCT / MCM6), шүдний морфологи (EDAR), пигмент (OCA2, SLC24A5) болон алкохолын метаболизмын (ADH1B) үйл ажиллагаа эсвэл хувьсал хөгжилтэй хамааралтай 5 дан нуклеотидийн полиморфизм (ген дэх нэг нуклеотидийн өөрчлөлт, SNP)-ийн хүн амын аллель (гомолог хромосомын ижил локуст байрлаж байгаа нэг генийн хоёр хувилбарыг хэлнэ) –ийн давтамжийг тооцоолж үзэв (Зураг 5A). Нэгдүгээрт, бэлчээрийн мал аж ахуйн амьдралын хэв маяг, сүү, цагаан идээг өргөн хэрэглэж байсан нь тогтоогдсон (Jeong et al., 2018; Wilkin et al., 2020a) хэдий ч хүрлийн дунд, хожуу үеийн болон төмрийн түрүү үеийн Зүүн бүсийн тал нутгаас олдсон антропологийн олдворуудад лактаз задлагч энзимийн ямар ч мутаци үүсээгүй байгааг илрүүлэв. Түүхэн дараагийн үеийн олдворуудад өнөөгийн Европт өргөн тархсан (rs4988235) мутаци илэрч байгаа ч маш бага давтамжтай (~ 5%) бөгөөд цаг хугацааны явцад ч энэ давтамж ихсээгүй байна (Зураг 5A). Орчин үеийн монгол малчид бусад цагаан идээнээс гадна зуны саруудад өдөрт 4-10 литр айраг (гүүний исгэсэн сүү, ~ 2.5% лактоз) хэрэглэснээр (Bat-Oyun et al., 2015) өдөр тутам 100-250г лактоз буюу сүүний чихэр хэрэглэдэг болохыг энд онцлох хэрэгтэй юм. Енисейн сав газарт олдсон айраг исгэж буй дүрслэл бүхий хадны сүг зургийг төмрийн эхэн үед хамруулдаг (Dėvlet, 1976) бөгөөд түүхэн сурвалжуудад эртний монголчууд айргийг байнга, ихээр хэрэглэдаг байсан төдийгүй хивэгч малын бусад хатуу, шингэн цагаан идээг арвин ихээр хэрэглэж байсныг тэмдэглэн үлдээсэн нь бий (Bayarsaikhan, 2016; Onon, 2005). Үүнийг мөн эртний уургийн (протеомик) судалгаагаар нотолсон болно (Jeong et al., 2018; Wilkin et al., 2020a). Сүүний чихэр боловсруулах чадамжгүй хирнээ монголчууд хэдэн мянган жилийн турш ийм их хэмжээний цагаан идээ, лактоз (сүүний чихэр)-ийг хэрхэн шингээн боловсруулж ирсэн нь тодорхойгүй байгаа боловч магадгүй энэ нь тэдний гэдэсний микробиомын ердийнхөөс өөр, лактоз задлагч *Бифидобактериар* баялаг бүтэцтэй (Liu et al., 2016) холбоотой байж болох юм.

Бүс нутгийн шалгарлын генетик маркерууд нь геномын өргөн хүрээний өвгийн профилийн өөрчлөлттэй уялдсан аллелийн давтамжийн өөрчлөлтийг харуулж байна (Зураг 5A). Жишээлбэл, эктодисплазин A рецептор – *EDAR* буюу хүний арьсны эдэд онцгой үүрэг бүхий уургийн ген (ectodysplasin A receptor), rs3827760 ба *SLC24A5* (уусдаг субстанс зөөвөрлөгч 24 –р бүлгийн 5-р гишүүн - solute carrier family 24 member 5) дэх rs1426654 гэсэн маркерууд нь Зүүн Ази, Баруун Евразийн хүн амд байх эерэг шалгарлын үед тохиолдох сайтар мэдэгдсэн бай юм (Sabeti et al., 2007). Бидний судалсан хүрлийн дунд, хожуу үеийн болон төмрийн түрүү үеийн хүн ам нь эдгээр хоёр дан нуклеотидийн полиморфизм (нэг генийн бүтэц дэх олон хувилбарт шинж тэмдэг)-ийн аллелийн давтамжийн хооронд хүн амын гүнзгий ялгаатай байдлыг харуулж байна: rs3827760 давтамж нь Зүүн Евразийн удамшлын хувь өндөртэй хүн ам, бүлгүүдэд (Khovsgol_LBA, Ulaanzuukh_SlabGrave) харьцангуй өндөр байсан бол r1426654 нь хүрлийн дунд, хожуу үеийн Алтай, төмрийн үеийн Чандманий соёлд илүү өндөр байна. (Хүснэгт S2E). Зүүн Азийн орнуудын дунд илүү сүүл үед тохиолдсон *ADH1B (альдегид дегидрогеназа 1B* - aldehyde dehidrogenase 1B*)-д байх* rd1229984 маркер ба *OCA2* (*Окулокутан альбинизм II* - oculocutaneous albinism II)-ийн rs1800414 маркеруудад бий болсон хоёр эерэг шалгарал болох хоёр дан нуклеотидийн полиморфизм (SNP) (Donnelly et al., 2012; Li et al., 2011) хүрлийн дунд, хожуу үе болон төмрийн түрүү үеийнхэнд байхгүй эсвэл маш бага давтамжтай байсныг олж тогтоов. Тухайн үед Зүүн Евразийн өвөг нь үндсэндээ Эртний Хойд Азиас хамааралтай байсан ч Хятад болон бусад орнуудтай харилцах холбоо хамаарал ихэссэнээс үүдэн Зүүн Азийн өвгийн хувь нь нэмэгдэх тусам давтамжууд ч мөн ихэсжээ (Хүснэгт S2E).

Эцэст нь бид Зүүн бүсийн тал нутгийн хүн амын генетикийн түүхэн дэх хүйстэй холбогдох асуудлыг авч үзлээ. Генетикийн холимог дахь хүйсийн хандлагатай шинжүүд нь шилжилт хөдөлгөөний жендэрийн байдал, нийгмийн ураг төрөл, гэр бүлийн бүтцийн талаар мэдээлэл цуглуулахад тус дөхөм болох талтай. Бид төмрийн түрүү үеийн Сагил-Уюк болон Түрэгийн үеийн бүлгүүдийн (Z стандарт онооны илүү эерэг утга; Зураг 5B) дунд Баруун бүсийн тал нутгийн эрэгтэй хандлагатай генетикийн хольц тодорхой байгааг ажиглав. Энэ нь мөн Y хромосомын Q1a удмын бууралт, R ба J зэрэг Баруун Евразийн удмын өсөлттэй таарч байгаа юм (Зураг S2A). Хятаны сүүл үе болон Монголын эзэнт гүрний үед Зүүн Азиас гаралтай өвгийн маш тодорхой эрэгтэй давамгайлсан хандлага ажиглагдаж байгааг Y хромосомын O2a удмын давтамжийн өсөлтөөс харж болохоор байна (Зураг S2A). Хүннүгийн үед маш нарийн төвөгтэй, эрэгтэй хандлага бүхий генетикийн бүрдэл ажиглагдаж байгаа ба хүн амын дундах өөр угсаатай бүлгүүд өөр өөр эцгийн генетикийн бүрдлээс үүдэлтэй болохыг харуулж байв (Зураг S2C).

Энэхүү судалгаанд хамрагдсан Хүннү гаралтай антропологийн олдворуудын дунд удамшлын холбоотой 10 хос олдвор илэрсэн нь: Жаргалантын амны булшинд аав-охин хоёрыг (JAG001 ба JAA001), Ильмийн хөндийн булшинд эх-хүү хоёрыг (IMA002 ба IMA005), Тамирын улаан хошууны булшинд ах/эрэгтэй дүү-эгч/эмэгтэй дүүгийн хосыг (TMI001 ба BUR003), Салхитын амны булшинд ах-эрэгтэй дүү хоёрыг (SKT002 ба SKT006) тус тус хамт оршуулсан байв (Хүснэгт S2D). Үлдсэн зургаан хосын гурав нь нэг оршуулгын газар оршуулагдсан дан эмэгтэй хамаатнууд байгаа нь Хүннү нарын дунд эмэгтэйчүүдийн өргөтгөсөн гэр бүл, ураг төрлийн холбоо байсныг харуулж байна. Нэг дурсгалд оршуулагдсан нэгдүгээр үеийн хамаатнуудын тухай Эгийн голын Хүннүгийн оршуулгын дурсгалаас илэрсэн олдворын аутосомын *STR* өгөгдөл дээр суурилсан судалгаанд (Keyser-Tracqui et al., 2003) мөн дурдсан байдаг. Эдгээр ураг төрлийн харилцааг оршуулгын зан үйлийн онцлог шинж чанаруудтай хослуулан авч

үзэх нь өнөөг хүртэл бага судлагдсан Хүннү нарын удам угсаа, ураг төрлийн бүтцийг тодруулахад чухал алхам болох юм.

Хэлцэмж

Зүүн бүсийн тал нутгийн хүн амын түүх бол олон удаа давтагдан явагдаж холилдсон зүүн ба баруун Евразийн олон янзын генийн сангуудаар тодорхойлогдоно. Зүүн бүсийн тал нутгийн хүн ам бүрэлдэх үйл явц нь Шилжилт хөдөлгөөний энгийн нэг давалгаа гэхээс илүүтэй маш нарийн төвөгтэй, хувьсамтгай шинж чанартай байв. 200 гаруй эртний хүний гений мэдээллийн санд тулгуурлан бид НТӨ 4600 оноос Монголын эзэнт гүрний төгсгөл үе хүртэлх хүн амын энэхүү динамик түүхийн анхны генетик нотолгоог танилцуулж буй болно. Холоцены дунд үед Зүүн бүсийн тал нутаг Эртний Хойд Ази болон Эртний Хойд Евразийн анчин түүвэрлэгч хүн амаас бүрдэж, дараа нь хүрлийн үед сүү, цагаан идээнд түшиглэсэн бэлчээрийн мал ахуй руу шилжжээ. Гаршуулсан мал, тэргээр хөлөглөсөн Ямная/Афанасьевын соёлт тал хээрийн малчид нүүдэллэн ирснээр (Kovalev and Erdenebaatar, 2009) энэ бүс нутагт НТӨ 3000 оны үед анх бэлчээрийн мал аж ахуйг нэвтрүүлсэн (Wilkin et al., 2020a) боловч Европ тивд үлдээсэн шигээ генетикийн тод ул мөр үлдээгээгүй нь гайхмаар (Allentoft et al., 2015; Haak et al., 2015; Mathieson et al., 2015).

Хүрлийн дунд, хожуу үе гэхэд угсаа гарлаасаа үл хамааран Зүүн бүсийн тал нутгийн ард түмнүүдийн дунд хивэгч малын сүүнд түшиглэсэн бэлчээрийн мал аж ахуй өргөн дэлгэрч (Wilkin et al., 2020a), энэхүү амьжиргааны хэлбэр цааш улам өргөжин, хүрлийн хожуу үед гүүний сүү ашиглаж эхлэн, Монголын эзэнт гүрний үед ингэ саах арга туршлагатай болсон нь (Wilkin et al., 2020a) өнөөг хүртэл уламжлагдан үлджээ (Bat-Oyun et al., 2015; Kindstedt and Ser-Od, 2019). Баруун зүгээс нүүдэллэн ирж байсан бүлгүүд сүүний чихэр боловсруулдаг лактаза энзимийн генетикийн шинжийг олон удаагийн нүүдлээрээ оруулж ирж байсан хэдий ч 5000 гаруй жилийн түүхэнд энэ бүс нутагт сүү шингээх генетик шинжийн шалгарал явагдсан гэх ямар нэгэн нотолгоо, баримт олдохгүй байгаа нь оньсого хэвээр байна. Энэ нь Азид лактозид дасан зохицох үйл явц өөр замаар явагдсаныг харуулж байгаа ба өнөөг хүртэл үүнийг тайлбарлаж чадахгүй байгаа билээ.

Хүрлийн дунд, хожуу үеэр Зүүн бүсийн тал нутгийн зүүн хэсэгт хүрлийн өмнөх үеийн Эртний Хойд Азийн залгамж, хойд хэсэгт хүрлийн өмнөх үеийн Эртний хойд Ази болон Эртний Хойд Евразийн хоорондох генетикийн хувилбар, баруун хэсгээр Баруун бүсийн тал нутгийн нүүдэлчдийн удамтай, Синташтагаас гаралтай, эзлэх хувь нь нэмэгдсэн шинэ өвгийн гэсэн гурван талт генетикийн бүтэц бүрэлдэн тогтсон юм. Европын Байлдааны сүхний соёлтой удамшлын холбоо бүхий баруун бүсийн ойт хээрийн Синташта соёл (Mathieson et al., 2015) нь хүрлийн металлурги, морин тэрэгний мэргэжилтнүүд байсан (Anthony, 2010) ба Зүүн бүсийн тал нутагт шинэ (ялангуяа морьтой холбоотой) технологи нэвтэрсэн нь уг бүс нутагт тэдний гарч ирсэнтэй холбоотой байж болно. Ялангуяа ХБХС-ын малтлагаас морийг ачлагад хэрэглэж байсан байж болох, тэр ч байтугай мөн уналгад ашиглаж байсан байж болохоор (Taylor et al. 2015) олон баримт нотолгоо олддог ба судалгааны дүн шинжилгээгээр тэдгээр амьтад болон Синташтагийн тэрэгт морьдын хооронд генетикийн холбоо байгааг илрүүлжээ (Fages et al., 2019). Хүрэл зэвсгийн үеийн Зүүн бүсийн тал нутгийн хүн амын дунд баруун-зүүн бүсийн генетикийн хүчтэй ялгарал мянга гаруй жилийн турш үргэлжлэн, төмрийн түрүү үеийн төгсгөл хүртэл хадгалагдсан ч морийг уналгад өргөнөөр хэрэглэж эхэлсэн (Drews, 2004), мөн дөрвөлжин булшны соёл гэх мэт зарим бүлгүүдийн эрчимт хөдөлгөөн энэ хуваагдмал генетикийн бүтцийг эвдэж эхэлжээ (Honeychurch, 2015). Үр

дүнд нь гурван том угсаа нийлэн, холилдсон бөгөөд энэ нь Хүннү гүрэн үүссэн үетэй давхцаж буй юм. Хүннү нар генетикийн хувьд маш олон янз байсан бөгөөд дээр нь шинэ шинэ угсаа гарлын эх үүсвэр болж Хятад, Төв Ази, Баруун бүсийн тал хээр (Сарматтай холбоотой)–ийн удам тэдний удмын санд хурдтай орж ирсэн байна.

Үүний дараах дундад зууны эхэн үеийн генетикийн мэдээлэл тун хомс бөгөөд тогтвортой бус юм. Хүннүгээс Түрэгийн үе хүртэлх 400 жилийн хугацаанд оршиж байсан Сяньби, Нируны цөөн хэдэн дурсгал олдсон байдаг. Бид Уйгур ба Түрэгийн үеийн хүн амууд генетикийн хувьд маш олон янз байсныг илрүүлсэн бөгөөд Уйгурын эзэнт гүрэн унасны дараа дундад зууны сүүл үеэр Зүүн Евразийн өвгийн эзлэх хувь нэмэгдсэн генетикийн эцсийн томоохон шилжилт болсныг бид баримтжуулсан бөгөөд энэ нь Зүүн хойд зүгээс Зүүн бүсийн тал нутаг руу тэлсэн Тунгус (Зүрчид) болон Монгол (Хятан, Монгол) хэлээр ярьдаг бүлгүүдийн тархалтын тухай түүхэн сурвалжуудтай таарч байна (Биран, 2012). Зүүн Азиас гаралтай уг гарал угсаа Дундад зууны хожуу үеийн хүн амд эрэгтэйчүүдээр илүү дамжин орж ирсэн болохыг бид тогтоов. Монголчуудын эрин үеийн төгсгөлд Зүүн бүсийн тал нутгийн генетикийн бүтэц эрс өөрчлөгдөж, нэн эрт үеийн гол онцлог болж байсан Эртний хойд Евразийн гаралтай удамшлаас багахан хэсэг нь хадгалагдан үлджээ. Өнөөдөр Эртний Хойд Евразийн удамшил зөвхөн Сибирийн алслагдмал бүлгүүд болон Америкийн уугуул иргэдийн дунд нэлээн хэмжээгээр хадгалагдан үлдсэн байна (Jeong et al., 2019). Түүхэн монголчуудын генетикийн төлөв байдал нь орчин үеийн монголчуудын дунд одоо хүртэл байгаа нь энэхүү генийн сан сүүлийн ~ 700 жилийн турш харьцангуй тогтвортой хадгалагдаж ирснийг харуулж байгаа юм.

Зүүн бүсийн тал нутагт гарсан генетикийн шилжилтийн гол гол үеүүдийг ийнхүү баримтжуулснаар, цаашид дээрх өөрчлөлтүүд нь соёл, технологийн шинэчлэлтэй холбоотой эсэх, мөн уг технологийн дэвшлүүд улс төрийн төрх байдалд хэрхэн нөлөөлсөн зэргийг судлах боломжтой болж байна. Дээрх судалгааны үр дүнгүүдийг адууны соёл, мал аж ахуйд түүнчлэн малын онцлог шинж чанар, үүлдэрт гарсан өөрчлөлттэй нэгтгэн үзвэл илүү сонирхолтой болох юм. Энэхүү судалгаа нь зүүн Евразийн тал хээрийн анхны өргөн цар хүрээт палеогеномын судалгаа болох бөгөөд тухайн бүс нутгийн гайхалтай хувьсамтгай генетикийн олон янз байдлыг нээн илрүүлж буй юм. Уг судалгаагаар энэ мэт олон ахиц дэвшил гарсан хэдий ч Евразийн нутаг дэвсгэр дээрх хүн амын түүхийг болон эртний дэлхийн түүхэнд тэдний гүйцэтгэсэн үүргийг бүрэн тодруулахын тулд цаашид төв ба зүүн Еврази, ялангуяа Хятадын зүүн хойд хэсэг, Таримын хотгор, Казахын зүүн хэсгийн тал нутгийг хамарсан генетикийн судалгаа хийх шаардлага урган гарч байна.

ТАЛАРХАЛ

Бид Д.Наваан, З.Батсайхан, В.Волков, Д.Цэвээндорж, Л.Эрдэнэболд, М.Хортон, Ш. Уранчимэг, Б.Наран, Н.Сэр-Оджав, П.Коновалов, Х.Лхагвасүрэн, Н.Мамонова, Э.Мижиддорж, Л.Намсрайдан, А.П. Окладников, Х. Пэрлээ, С.Данилов болон Д.И. Бураев нарын энэхүү судалгааны археологийн судалгааны ажилд оруулсан хувь нэмэрт нь талархал илэрхийлье. Мөн Монгол Улсын Их Сургуулийн Антропологи, археологийн тэнхим, Буриадын Шинжлэх Ухааны Төвийн музейд энэхүү төсөлд хамтарч ажилласанд талархал илэрхийлж байна. М.Блездэйлд эртний гар бичмэлийн ноорогт санал, зөвлөгөө өгсөнд, Г. Сумьяад дээж цуглуулах ажилд туслалцаа үзүүлсэнд, С.Нагель, М.Мэйер нар нэг гинжит ДНХ (ssDNA) мэдээллийн сан бэлтгэхэд

туслалцаа үзүүлсэнд мөн С.Накагомэд нарийвчлал багатай генийн дарааллын өгөгдлөөс популяцийн аллелийн давтамжийг тооцоолох скриптийг хуваалцсанд талархаж байна. Мөн П.Морианид өгүүлэл нийтлэгдэхээс өмнө *DATES* програмд нэвтрэх эрх олгосонд баярлалаа. **Зөвшөөрөл**: Энэхүү судалгаанд ашигласан эртний хүний яс, шүдний олдворын дээжүүдийг Монгол Улсын Соёлын яамны *№18* дугаартай зөвшөөрөл, Монгол Улсын Боловсрол, Соёл, Шинжлэх ухаан, Спортын Яам, Монголын Худалдаа, Аж Үйлдвэрийн Танхимын *№ A0122772 MN DE 0 8124, A0109258 MN DE 7 643, A0117901 MN DE 9 4314* дугаартай Гарал үүслийн бичиг, МГЕГ-ын *№12-2091008-20-E00225R* дугаарт мэдүүлгээр шалган баталгаажуулсан болно. **Санхүүжилт:** Энэхүү судалгааг Макс-Планкийн Нийгэмлэг, Монгол Улсын БСШУСЯ-ы ШУТСан (2015/25 М.Э, К.С, Д.Т), Оросын Суурь Судалгааны Сан (Grant #18-59-94020 E.M., K.S., D.T.), ОХУ-ын Боловсрол, Шинжлэх Ухааны яам (NW, BB, DM, N. PK), АНУ-ын Шинжлэх Ухааны Үндэсний Сан (BCS-1523264-аас CW), Германы Судалгааны Хүрээлэн (SFB 1167 № 257731206-аас JB), Солонгосын ЗГ-ын Үндэсний Судалгааны Сан (MSIT) (2020R1C1C1003879 to C.J.), Европын Судалгааны Консулаас Европын Холбооны Мэдлэгийн цар хүрээ 2020 – судалгаа, шинэ нээлтийн хөтөлбөрийн хүрээнд, *771234-PALEORIDER* (W.H.) болон *804884-DAIRYCULTURES* (C.W.) дугаар бүхий тэтгэлэг дор санхүүжүүлсэн болно.

ЗОХИОГЧДЫН ОРУУЛСАН ХУВЬ НЭМЭР

Кристина Вариннер, Чунвон Жүнг, Николь Бойвин нар судалгааны төлөвлөгөөг боловсруулж; Кристина Вариннер; Чунвон Жүнг нар судалгааны ажлыг удирдан; Мягмарын Эрдэнэ, Шевин Вилкин, Жессика Хэнди, Ян Бенманн, Содномын Өлзийбаяр, Волфганг Хаак, Николай Крадин, Билигто А. Базаров, Дэнис Миягашев, Прокопий Б. Коновалов, Елена Жамбалтарова, Алисиа Вентреска Миллер, Николь Бойвин болон Кристина Вариннер нар уг судалгааг судалгааны материал, эх хэрэглэгдэхүүнээр хангав. Брайн К. Миллер, Виллиам Тимоти Трэал Тэйлор, Ян Бенманн, Т.Т, Мягмарын Эрдэнэ нар археологийн дүн шинжилгээг хийж, Рафеалла Штал болон Челсиа Чиовелли нар генетикийн лабораторийн ажлыг гүйцэтгэсэн болно. Кэ Ванг, Чунвон Жүнг, Флориан Кнолле болон Стефан Шиффэлс нар генетикийн лабораторийн дүн шинжилгээг боловсруулж, Брайн К. Миллер, Кэ Ванг, Виллиам Тимоти Трэал Тэйлор, Чунвон Жүнг болон Кристина Вариннер нар археологийн болон генетикийн дүн шинжилгээнүүдийг нэгтгэн боловсруулсан болно. Кэ Ванг, Чунвон Жүнг болон Кристина Вариннер нар уг өгүүллийг Бриан К. Миллер, Виллиам Тимоти Трэал Тэйлор, Ян Бенманн нарын болон бусад зохиогчдын тусламжтайгаар бичив.

ХУВИЙН АШИГ СОНИРХОЛ

Зохиогчид ямар нэгэн хувийн ашиг сонирхол илэрхийлээгүй болно.

Хавсралт зураг

Зураг. 1. Эртний хүн амын бүлгүүд болон он цагийн ерөнхий зураглал. (**a**), Археологийн дурсгалт газруудын байршлыг холбогдох соёл, он цагаар ялган өнгөөр тэмдэглэв: Хөх ягаан өнгөөр Хүрлийн өмнөх үеийг; улаанаар Хүрлийн түрүү үеийг; хөх өнгөөр Хүрлийн дунд, хожуу үеийг; ягаанаар Төмрийн түрүү үеийг; ногоон өнгөөр Хүннүг; Дундад зууны үеийн эхэн үеийг бор өнгөөр; алтлаг өнгөөр Дундад зууны сүүл үеийг тус тус тэмдэглэв (Хавсралт материалаас үзнэ үү). Дурсгалт газрын код болон товчилсон нэршлийг Зураг S1A болон Хүснэгт S1B –ээс үзнэ үү (**b**), Судалгаанд ашигласан Евразийн бүс нутаг (дөрвөлжин хүрээгээр тэмдэглэсэн) болон бусад эртний хүн амын бүлгүүдийн байршил, он цагийг өнгөөр ялгав. Баруун болон Төвийн бүсийн тал нутгийг цайвар бор өнгөөр, харин Зүүн бүсийн тал нутгийг цайвар ногоон өнгөөр үзүүлэв. (**c**) Монголын археологийн соёлууд, тэдгээрийн он цагийн хэлхээс. Шугам дээр давхцаж буй дурсгалуудыг илүү тодруулан ялгахын тулд газар зүйн байрлалыг нь бага зэрэг гажуудуулсан болно.

Зураг. 2. Монголын генетикийн бүтэц он цагаар. Орчин үеийн Еврази (саарал тэмдэгт)-д хамаарах гурван гол түүхэн үед багтах антропологийн олдворуудын (n = 214) Үндсэн бүрэлдэхүүний дүн шинжилгээ (PCA). Үндсэн талбар дээр Үндсэн бүрэлдэхүүн -1 –ийг Үндсэн бүрэлдэхүүн - 2-той харьцуулж; дагалдах талбарт Үндсэн бүрэлдэхүүн-1-ийг Үндсэн бүрэлдэхүүн-3-тай харьцуулан харуулав. Үндсэн бүрэлдэхүүн -1-ийн дагалдах талбарын тэмдэглэгээнүүд нь үндсэн талбаруудын тэмдэглэгээнүүдтэй дүйцэж буй болно; Үндсэн бүрэлдэхүүн -3 нь 0.35% -ийн вариацтай байв. Хүн ам, дээж болон тэнхлэгийн нэршлүүдийг Зураг S3B –аас үзнэ үү, мөн дурсгалт газар болон дээжийн тухай дэлгэрэнгүй мэдээллийг хүснэгт S1B-C, S2A-ээс харна уу.

Зураг. 3. *qpAdm*-ээр тодорхойлогддог цаг хугацааны явцад өөрчлөгдөх Зүүн бүсийн тал нутгийн генетикийн өөрчлөлтүүд. a) Хүрэл зэвсгийн өмнөх үеэс Хүрлийн түрүү үе хүртэл ; (b) Дунд / хожуу хүрэл зэвсгийн үе; (c) Төмрийн түрүү үе; (d) Хүннүгийн үе; e) Дундад зууны эхэн үе; (f) Дундад зууны сүүл үе. Генетикийн холимогт эзлэх удамшлын харьцааг дээжийн хэмжээгээр илэрхийлэгдэх дугуй диаграммаар үзүүлж, удамшлынх нь эх үүсвэрийг доор харуулав (Хавсралт материалыг үзнэ үү). Хэсэг тус бүрийн дээжийн хэмжээг баруун дээд хэсэгт харуулав. Хэсэг (b-c) дээр Baikal_EBA цайвар цэнхэр өнгөөр загварчлагдсан; хэсэг (d-f) дээр, Хөвсгөл_LBA (нил ягаан), Chandman_IA-ийн Сагил / Уюк (ягаан) нь генетикийн шинэ эх үүсвэр болон тэмдэглэгдэв (Зураг 4). Соёлын бүлгүүдийг тод бичвэрээр зааж өгсөн болно. Хэсэг (d-f) дээрх антропологийн олдворууд нь өөрөөр тусгайлан тэмдэглээгүй бол Хожуу Хүннү, Түрэг, Монголын үед хамаарна. Өмнө нь судлагдсан хүн амыг цагаан бичвэрээр тэмдэглэсэн ба бусад нь бүгдээрээ уг судалгаанд судлагдсан болно. Газрын зургийн хилээс гадуурх хүн амыг сумаар зааж үзүүлэв. Давхардсан антропологийн олдворуудын харагдах байдлыг сайжруулахын тулд булшнуудын байрлалыг гажуудуулан байрлуулсан билээ.

Зураг. 4. Шинээр бүртгэгдсэн бүх генетикийн бүлгүүдийн өвгийн генетик өөрчлөлтийг он цагийн дараалаар үзүүлсэн нь. (A) Нэн эртний болон (B) бичмэл түүхийн үеүдийн бүлэгт суурилсан популяцийн генетикийн дүн шинжилгээний загварчлал. Генетик бүлэг тус бүр дэх антропологийн олдворуудын тоог Хүснэгт S3A-д үзүүлэв. Удамшлын харьцааны боловсруулаагүй мэдээлэл болоод стандарт алдааны тооцооллуудыг Хүснэгт S5-д үзүүлэв. Хэвтээ багана нь *qpAdm*-ээр тооцоолсноор ± 1 стандарт алдааг (SE) илэрхийлнэ.

Зураг. 5. Генетикийн холимог бүрдлийн үйл ажиллагааны аллелийн давтамж ба хүйсийн хэв шинж. (a) Таван фенотипийн дан нуклеотидийн полиморфизм (SNP)-ийн аллелийн давтамжийг он цагаар илэрхийлэв. Үр дүнтэй аллелийн хувьд бид магадлалын давтамжийн тооцоолол, эртний бүлэг тус бүрт баганаудад нэг стандарт алдааг заасан утгыг харуулсан. Хүрлийн дунд, хожуу үеэс өмнөх ангилал нь Мөнххайрханы өмнөх бүх эртний бүлгүүдийн нийлбэртэй тохирч байна. Хүннү, дундад зууны эхэн болон сүүл үе нь он цаг тус бүртэй дүйцэх, эртний бүх бүлгүүдийн нийлбэртэй таарч байна. Хэвтээ тасархай шугамууд нь 1000 Геномын төслийн таван хэт хүн амын аллелийн давтамжийн мэдээллийг харуулж буй болно. b) Генетикийн холимог бүрдлийн хүйсийн хандлагын хэв шинжийг он цаг, хүн амаар нь харуулав. Бид аутосом хромосомын түвшинд генетикийн холимог бүрдэлтэй эртний антропологийн олдвор тус бүрийн Z стандарт онооны утгыг тооцоолж үзэв. Z стандарт онооны эерэг утгууд нь аутосомууд дээр Баруун бүсийн тал нутгийн нүүдэлчид, Иран, Хань угсаанаас гаралтай удамшил илүү их байгааг буюу эрэгтэй давамгайлсан генетикийн холимог болохыг харуулж байна. Антропологийн олдвор тус бүрийн Z стандарт онооны утгуудыг Зураг S2C-ээс үзнэ үү.

We thank Zoljargal (Zoloo) Enkh-Amgalan for her translation of this paper. Help with reviewing the translation was provided by Dr. Enkhee Purev (Nagoya University, Laboratory of Bioactive Molecules), Prof. Erdene Myagmar (Department of Anthropology and Archaeology, National University of Mongolia), and Prof. Turbat Tsagaan (Institute of Archaeology, Mongolian Academy of Sciences) and Björn Reichhardt (MPI-SHH, Humboldt-University of Berlin).

Монгол хэлний орчуулга
Уг өгүүллийг Энх-Амгалангийн Золжаргал (Макс-Планкийн Хүн Төрөлхтний Түүхийн Шинжлэх Ухааны Хүрээлэн) Монгол хэлнээ орчуулсан бөгөөд хувь нэмрээ оруулан, хянаж, зааж зөвлөсөн Пүрэвийн Энхээ (Нагояагийн Их Сургуулийн Биологийн идэвхит молекулын лабораторын судлаач, Доктор), Мягмарын Эрдэнэ (Монгол Улсын Их Сургуулийн Антропологи, Археологийн тэнхим, Доктор, Профессор), Цагааны Төрбат (Шинжлэх Ухааны Академийн Археологийн Хүрээлэн, Доктор, профессор) болон Бёорн Райхардт (Макс- Планкийн Хүн Төрөлхтний Түүхийн Шинжлэх Ухааны Хүрээлэн, Хумболдтын Их сургуулийн докторант) нарт талархал илэрхийлэе.
